# A series of structurally related hcp-like silver clusters suggests faceted lateral cluster growth

**DOI:** 10.1039/d6sc06118d

**Published:** 2026-08-06

**Authors:** Mike Alexander Kordan, Claudio Schrenk, Sami Malola, Hannu Häkkinen, Andreas Schnepf

**Affiliations:** a Institut für Anorganische Chemie, Universität Tübingen Auf der Morgenstelle 18 72076 Tübingen Germany andreas.schnepf@uni-tuebingen.de; b Department of Physics, Nanoscience Center, University of Jyväskylä 40014 Jyväskylä Finland; c Department of Chemistry, Nanoscience Center, University of Jyväskylä 40014 Jyväskylä Finland

## Abstract

While nanoparticles and metalloid clusters are increasingly important, their formation and growth remain incompletely understood. Due to the enormous inherent complexity, the elucidation of the respective processes is usually limited to templated reactions, approximate modelling and indirect mechanistic inference. Herein, we report the synthesis of a series of metalloid silver clusters with 128, 132, 146 and 160 silver atoms within their respective silver cores. All compounds are highly unstable towards light and temperature. Nevertheless, we were able to confirm their structure using single-crystal X-ray diffraction. We discovered strong similarities between them, *e.g.* an overall hexagonal-prismatic shape, an unusual hcp-derived arrangement of the silver atoms within their core and the absence of partly occupied lateral facets. The title compounds form from near-identical conditions. Accordingly, a common reaction pathway in which lateral growth occurs in discrete steps seems plausible. As no thiolates were used during the synthesis, only chlorides and phosphines act as stabilizing ligands. The position of the chloride ligands within the molecules might prove to be essential for the faceted growth of the title compounds. As quantum chemical calculations indicate small Kohn–Sham frontier-orbital gaps, the structure and composition might be mostly governed by geometric factors, providing insights into the formation process of faceted nanoparticles on an atomic scale.

## Abstract 2

The synthesis and structural characterization of four structurally similar metalloid silver clusters with 128, 132, 146 and 160 silver atoms give insight into the mechanism of the formation of faceted silver nanoparticles on an atomic scale.

## Graphical abstract

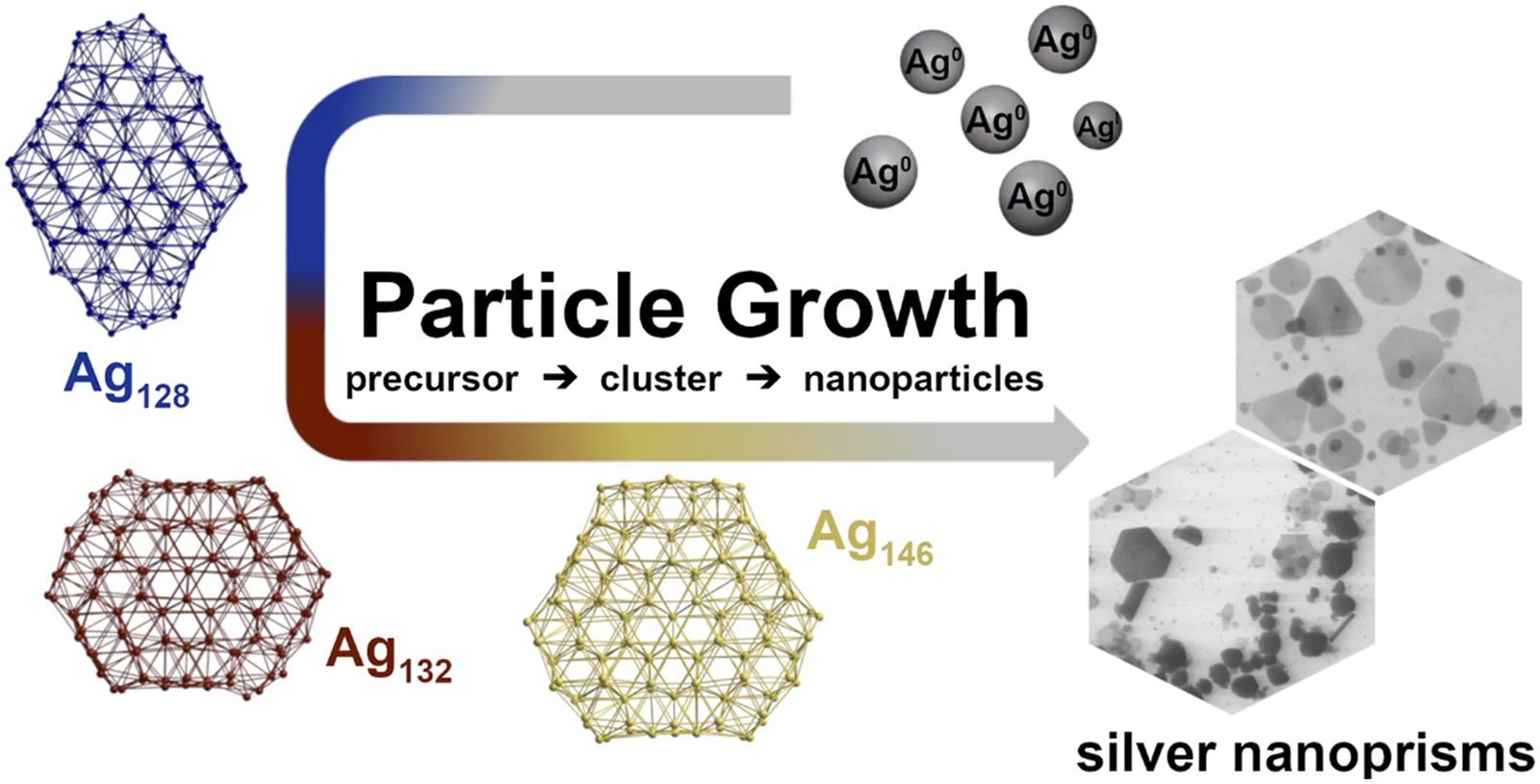

## Introduction

Metal nanoparticles and clusters, especially those of coinage metals, are of growing interest both for basic academic research and applied sciences. They often feature interesting properties so that they are suitable for applications in catalysis,^1^ energy conversion,^2^ sensing^3^ or medicine,^4^ to name only a few. While many experimental and theoretical studies elucidating nanoparticle formation exist, *i.e.* the nucleation process and the formation of larger structures from seeds,^5,6^ the underlying complexity and sheer size of the systems preclude detailed, definitive conclusions. For example, the morphology and properties of nanoparticles can be influenced by a myriad of factors, including kinetics, ligand chemistry, and particle size, many of which interact in complex ways.^7^

In this field, metalloid clusters with the composition M_*n*_R_*m*_ (*n* > *m*, R = ligands, *e.g.* silyls, phosphines, halides, thiolates) are ideal model compounds for more extensive investigations, shedding light on this nanoscale regime.^8^ By decreasing the size of the examined particles from 10–100 nm for nanoparticles to 1–10 nm for metalloid clusters (also often called nanoclusters), the complexity can be decreased by orders of magnitude. Furthermore, nanoparticles exhibit a size distribution. Consequently, they can be viewed as an ensemble containing a distribution of clusters of different size, shape and composition. In contrast, metalloid clusters feature an atom-precise composition and a defined shape, further simplifying experiments and their interpretations.^9^ Due to the profound structure–property relationship, knowledge of the cluster morphology is imperative.^10^

**Fig. 1:**
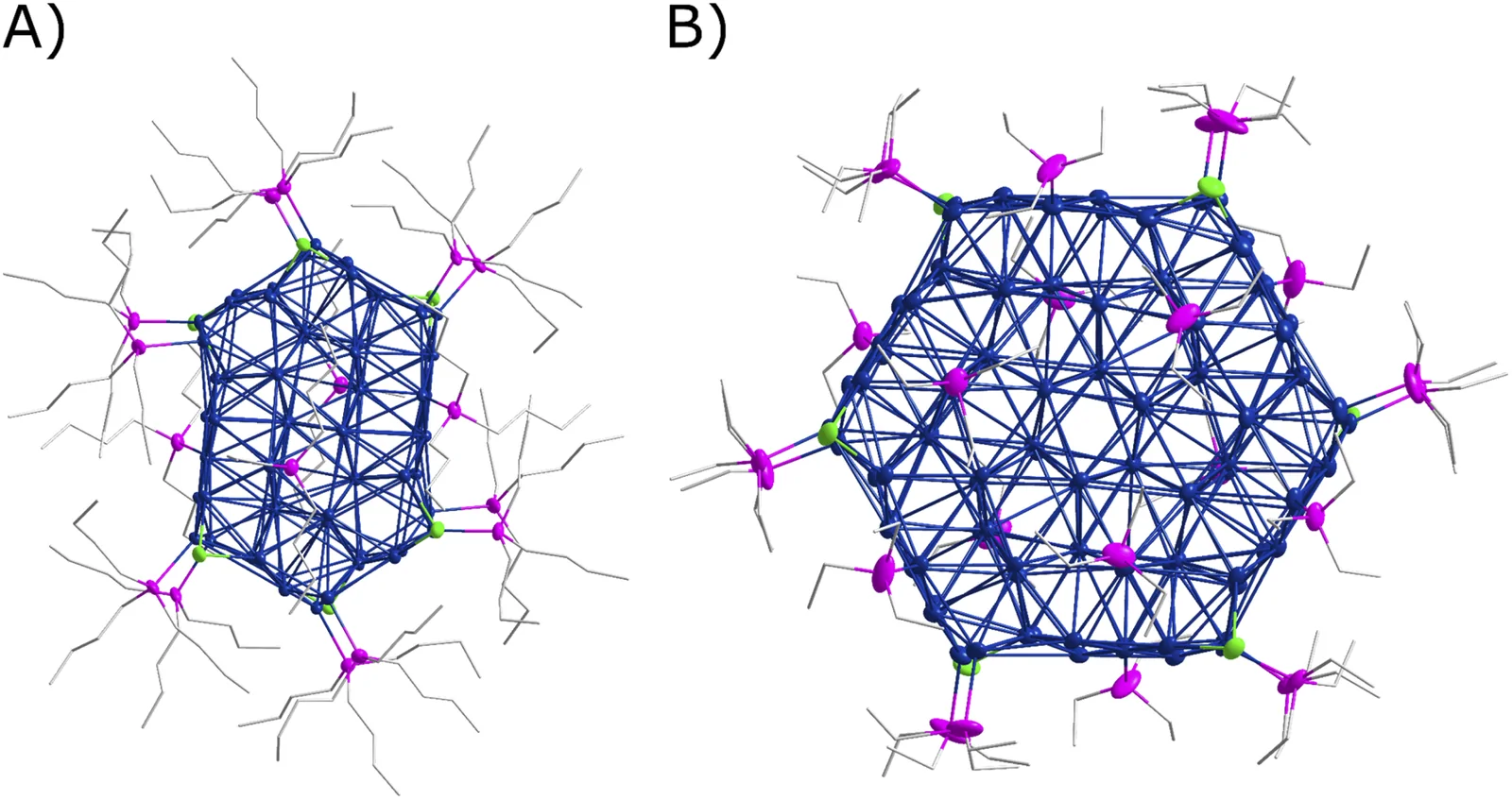
Molecular structures of (A) Ag_64_ and (B) Ag_108_. Ag (blue), P (pink), Cl (green); thermal ellipsoids are set at 50% probability.

As the name suggests, metalloid clusters often feature structures found in the bulk metal.^11^ For silver, which realizes a fcc packing, several cluster compounds with similar features are known, *e.g.* [Ag_63_(SC_6_H_3_-3,4-F_2_)_26_(PBu_3_)_8_]^+^, [Ag_67_(SC_6_H_3_-2,4-(CH_3_)_2_)_32_(PPh_3_)_8_]^3+^ or [Ag_100_(SC_6_H_3_-3,4-F_2_)_28_(PPh_3_)_8_]^−^ among others.^12–15^ Besides those, other structures featuring decahedral and icosahedral cluster kernels are known as well. Remarkably, such non-fcc-like structures are realized within the two largest structurally characterized metalloid silver clusters to date, Ag_374_(SC_6_H_4_-4-^*t*^Bu)_113_Br_2_Cl_2_ (ref. 16) and [Ag_429_Cl_24_(C≡C_6_H_4_-4-CF_3_)_150_][PPh_4_]_5_.^17^ While the decahedral and icosahedral structures are not a typical geometry found in bulk silver, both might be described as fivefold- and twentyfold-twinned fcc structures. While many core motifs are known, the overall morphology of metalloid clusters tends to be more spherical the larger the cluster becomes. Hcp packing, not known for bulk silver, is seldom realized within silver clusters. Besides Ag_19_, Ag_25_,^18^ Ag_50_ (ref. 19) and Ag_64_(PBu_3_)_16_Cl_6_ (Ag_64_),^20^ Ag_108_(PEt_3_)_24_Cl_6_ (Ag_108_)^21^ is the only structurally characterized example exhibiting an extended structure reminiscent of hcp packing (Fig. 1). While it is suggested that slow kinetics and size confinement may favor hcp stacking,^22^ there are too few examples to justify any definitive conclusions.

Consequently, we were interested in the synthesis of more clusters of this elusive compound class. Since we already had success with the adaptation of our reaction protocol for Ag_64_, resulting in the hexagonal-prismatic Ag_108_ cluster, we used yet another phosphine, *i.e.* PPr_3_, for further investigations. To our surprise, the reaction did not yield one single compound but, depending on factors not yet identified, four different clusters Ag_128_(PPr_3_)_28_Cl_6_ (Ag_128_), Ag_132_(PPr_3_)_28_Cl_8_ (Ag_132_), Ag_146_(PPr_3_)_30_Cl_6_ (Ag_146_) and Ag_160_(PPr_3_)_*x*_Cl_*y*_ (Ag_160_) could be identified so far. This behavior emphasizes the inherent complexity of metalloid cluster synthesis, indicating that small deviations in synthesis and work-up can lead to different cluster compounds.

Remarkably, all four compounds (Ag_128_, Ag_132_, Ag_146_ and Ag_160_) feature closely related structures, namely an overall hexagonal-prismatic shape, hcp stacking within the silver core and chlorides bound only to the cluster edges. This suggests that the clusters follow a similar reaction path towards larger clusters, namely faceted growth, a phenomenon already known from nanoparticles.^23^ Further thermolysis experiments show that these clusters can lead to larger faceted nanoparticles, rendering the isolated clusters as atomically resolved structural models, providing for the first time a direct insight into the formation process of faceted nanoparticles with atomic precision.

## Syntheses

As is known from previous investigations, even small changes in the ligand can play a vital role in determining the resulting cluster size and shape.^24,25^ In this regard, ligands with low steric demand often arrange themselves in close proximity to each other, which in turn can favor larger metal cores.^24*c*,26,27^ We were able to observe this trend in our syntheses of Ag_64_ and Ag_108_, where PBu_3_ and PEt_3_, respectively, were used as ligands. To test whether this holds true, we synthesized the precursor (Pr_3_P)AgCl and reduced it with l-selectride (Li[HB(*sec*-Bu)_3_]). The reaction mixtures were maintained at low temperature; depending on the experiment, they were allowed to warm to final temperatures between −30 °C and −5 °C, while crystallization and storage were conducted at −30 °C. Where possible, experiments were conducted in a dark room equipped with a red safelight (630 nm). Instead of one single product for all reactions, we were able to isolate the structurally related compounds Ag_128_, Ag_132_, Ag_146_ and Ag_160_ as dark red crystals. It is noteworthy that for every single reaction, no product mixtures were observed but only a single compound was obtained in crystalline form respectively. This was confirmed by extensive SCXRD experiments: for several reactions, we measured the cell constants for at least ten crystals collected from various points of the glass vessel. Within each batch, no second crystalline phase was detected among the sampled crystals. So far, we cannot predict the resulting product *a priori*. We have conducted several experiments to identify any possible factors that determine the final product. However, the results were inconclusive. For an extended discussion on this topic, see the SI.

### Molecular structure determination *via* single-crystal X-ray diffraction

Many reaction batches yielded products in the form of single crystals unsuitable for SCXRD experiments, whereby regularly even the unit cell dimensions were undeterminable. Recrystallization did not prove to be fruitful due to the low or negligible solubility of the title compounds once crystallized. Many crystallographic problems can be attributed to intrinsic defects such as disordered phosphines. Another factor is the rather small size of some crystals, often exhibiting a needle-like crystal habit with two of the dimensions being well below 100 µm. Despite these limitations, we elucidated the molecular structure of compounds Ag_128_, Ag_132_, and Ag_146_. For compound Ag_160_, the data quality only allows statements regarding the connectivity of the silver atoms within the cluster core. Also, in the case of Ag_146_ the organic propyl groups in the ligand shell could not be fully refined. For further information regarding the crystal data and refinement, see the crystallographic section of the SI.

For the X-ray diffraction experiments, the dark red single crystals of the title compounds were selected in precooled perfluorated oil under a dimmed light microscope. This prevents decomposition during the selection process. Afterwards, crystal mounting took place as quickly as possible. X-ray crystal structure analysis identified the red crystals as Ag_128_(PPr_3_)_28_Cl_6_ (Ag_128_), Ag_132_(PPr_3_)_28_Cl_8_ (Ag_132_), Ag_146_(PPr_3_)_30_Cl_6_ (Ag_146_) and Ag_160_(PPr_3_)_*x*_Cl_*y*_ (Ag_160_).

### The molecular structure of Ag_128_

**Fig. 2:**
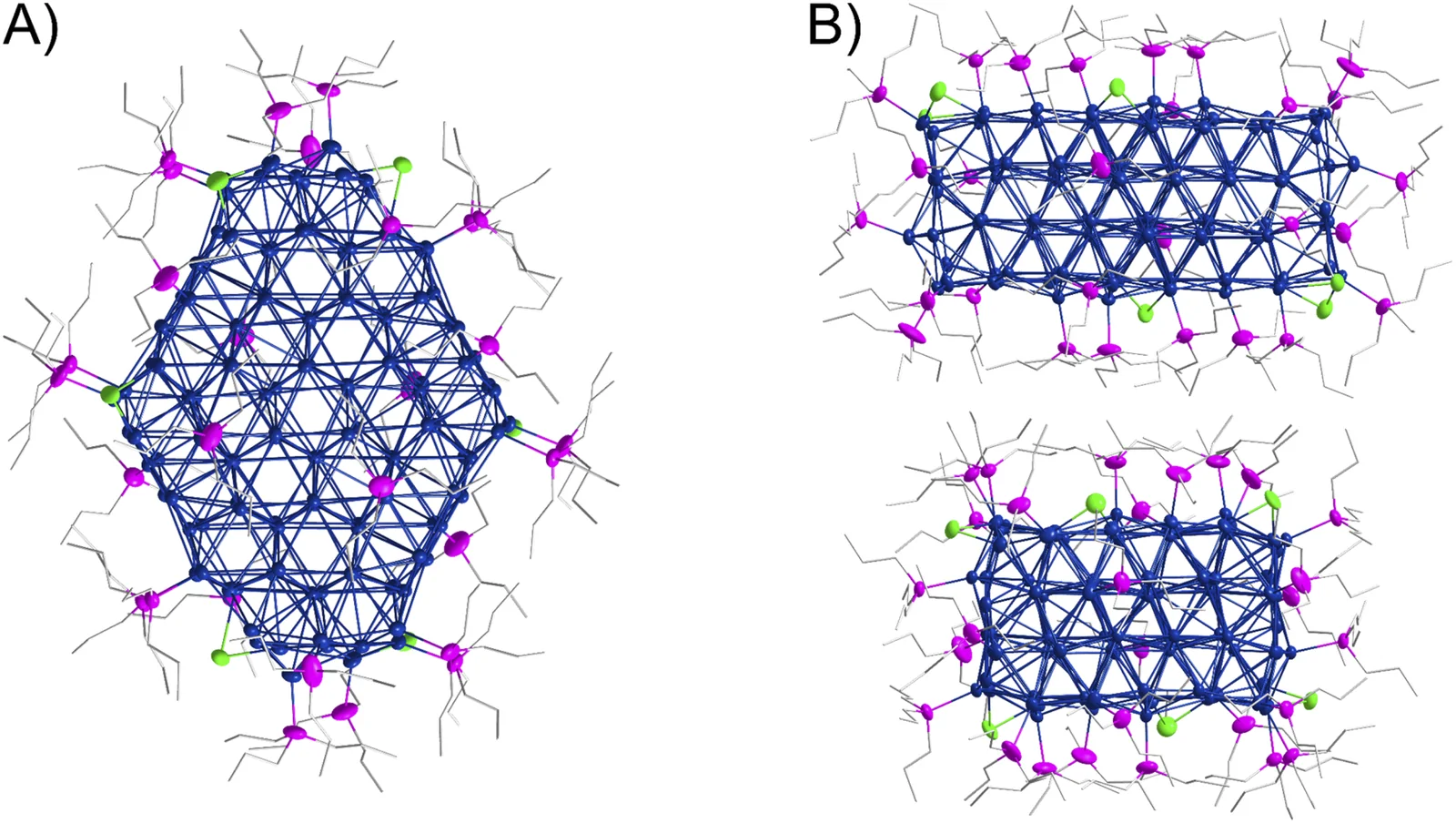
Molecular structure of Ag_128_(PPr_3_)_28_Cl_6_. (A) Top view. (B) Side views. Ag (blue), P (pink), and Cl (green); thermal ellipsoids are set at 50% probability. C atoms (grey) are shown as wireframe model. H atoms are omitted for clarity.

Ag_128_ crystallizes in the triclinic space group *P*1̄ with half a molecule in the asymmetric unit. The silver core adopts the shape of an irregular concave hexagonal prism of *C*_i_ symmetry with the prism sides measuring 0.6 nm, 0.9 nm and 1.1 nm respectively (Fig. 2, see Table S6 and Fig. S5 for more detailed values and a visualization for all clusters' dimensions). The sum of the interior angles within a hexagon equals 720°. Therefore, the angle sum of 719° found within the metal kernel and individual angles of approximately 117–123° are consistent with describing Ag_128_ as a hexagonal prism. The kernel spans an overall length of 1.9 nm and width of 1.6 nm. Meanwhile it only measures 0.77 nm in height, resulting in a rather flat, semi-2D appearance. This is unusual, as systems of similar size tend to adopt a rather spherical shape.^28^ Nonetheless, some metalloid silver clusters feature a rather flat shape as well, for example Ag_93_ reported by Hu *et al.* and Ag_102_ reported by Sun and co-workers.^29^ So far, the title compounds and the recently reported Ag_108_ are the only examples of hexagonal prismatic metalloid clusters. As the number of known compounds and therefore knowledge of their structure–property relationships grows, they might prove to be ideal seeds for the formation of hexagonal silver nanoplates,^21,30^ a compound class of recent interest.^31^

**Fig. 3:**
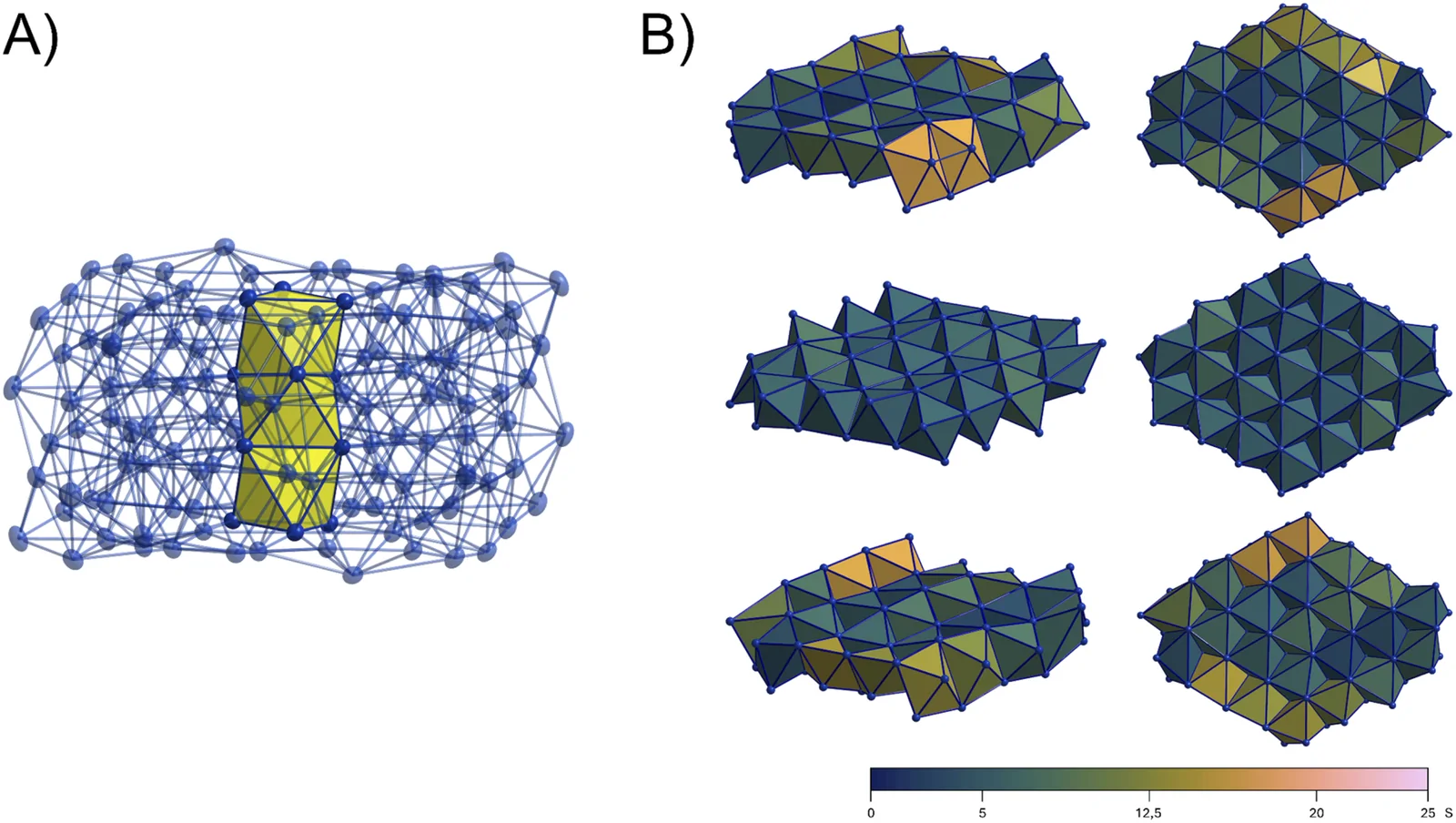
(A) Example of face-sharing octahedra found within Ag_128_. (B) Heatmap of calculated continuous shape measures *S*. A lower *S* suggests a geometry closer to an ideal octahedron.

The overall morphology is not the only property linking Ag_128_ and Ag_108_ though. Face-sharing Ag_6_ octahedra, as described for Ag_108_, can also be found in Ag_128_. Therefore, the metal core structure can be described as distorted hexagonal close-packed (Fig. 3A). To quantify the extent of the distortion, continuous shape measure^32^ calculations were performed using the SHAPE 2.1 program^33^ for every Ag_6_ octahedron present in the cluster kernel (for in-depth analysis, see the SI). While the values agree with literature data for idealized six-vertices polyhedra, especially octahedra, the overall results suggest a rather large deviation from the ideal octahedron in most cases.^34^ Still, the ensemble of octahedra is less distorted in Ag_128_ than in Ag_108_ (Fig. S6). This suggests that increasing cluster size is associated with more metal-like structural regularity. This applies to features such as a regular lattice and not the actual structure, since only fcc packing is known for bulk silver.^35^

As can be seen from the resulting distortion heatmap (Fig. 3B), the areas most distorted are located on the edges of the metal core, with the inner region showing less distorted octahedra.

**Fig. 4:**
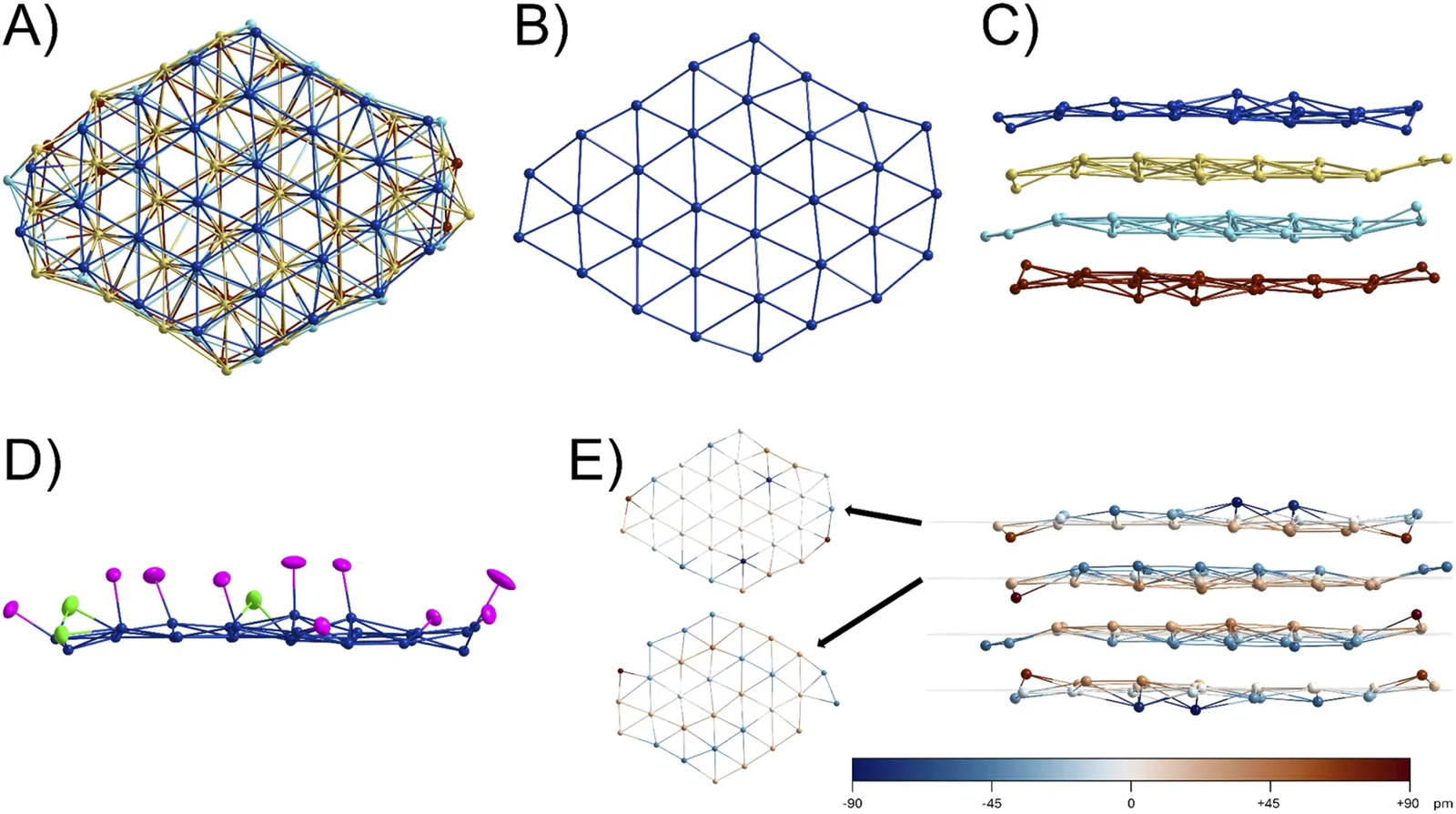
(A) Top view of the Ag_128_ core. The atom positions of the first (blue) and third (cyan) as well as those of the second (yellow) and fourth (red) layers are near-congruent, resulting in an ABAB-like stacking. (B) Top view of an outer Ag_32_ layer. (C) Side view of the individual Ag_32_ layers. (D) Side view of an outer Ag_32_ layer, including P and Cl atoms. (E) Heatmap of the calculated out-of-plane undulation (blue: outwards, red: inwards).

Besides the internal structure, the overall kernel morphology is of interest. Similar to its smaller congener Ag_108_, Ag_128_ can be described by four layers stacked in ABAB fashion (Fig. 4A). Each individual Ag_32_ layer – viewed along the stacking axis – resembles a slightly distorted {0001} facet (Fig. 4B). The Ag–Ag distances vary from 270 to 335 pm (average: 307.7 pm; bulk: 289 pm) and are in good agreement with our earlier findings.

The side view shown in Fig. 4C shows that the individual Ag_32_ layers are strongly undulated, which does not support the idea of a nearly perfect hexagonal close-packed cluster core. Hence, the hexagonal close-packed cluster core is strongly distorted. In the case of the outer layers, Ag atoms are pulled out-of-plane up to 88 pm (Ag_108_: 80 pm). This holds true for the inner layers as well. In general, we attribute the distortion to surface reconstruction, a phenomenon not only known for bulk materials such as silicon or gold^36*a*,*b*^ but also for nanoparticles such as gold nanorods.^36*c*^ The bonding of the phosphine ligands to the coordinatively unsaturated outer silver atoms alleviates this unsaturation in three ways: (1) the phosphine donates electron density to the bound Ag atom, easing its coordinative and electronic deficit. (2) The coordination of the phosphine ligand allows the coordinated silver atom to rise above the remainder, decreasing the surface exposure of the surrounding silver atoms (Fig. 4D). (3) The resulting undulation also lowers the steric crowding caused by the tightly spaced phosphine ligands. As the inner Ag atoms are coordinatively saturated, they do not gain significant energy from distortion, only following the surface atoms. As can be seen in Fig. 4E, the main undulation is located at or near ligand-bearing atoms, whereby the inner layers only feature strong distortion on the edges. Overall, the inner Ag_32_ sheets are less undulated than the outer ones. Consequently, the undulation on the surface also affects the arrangement of atoms deep within the cluster core. For further information on undulation calculations, see the SI.

**Fig. 5:**
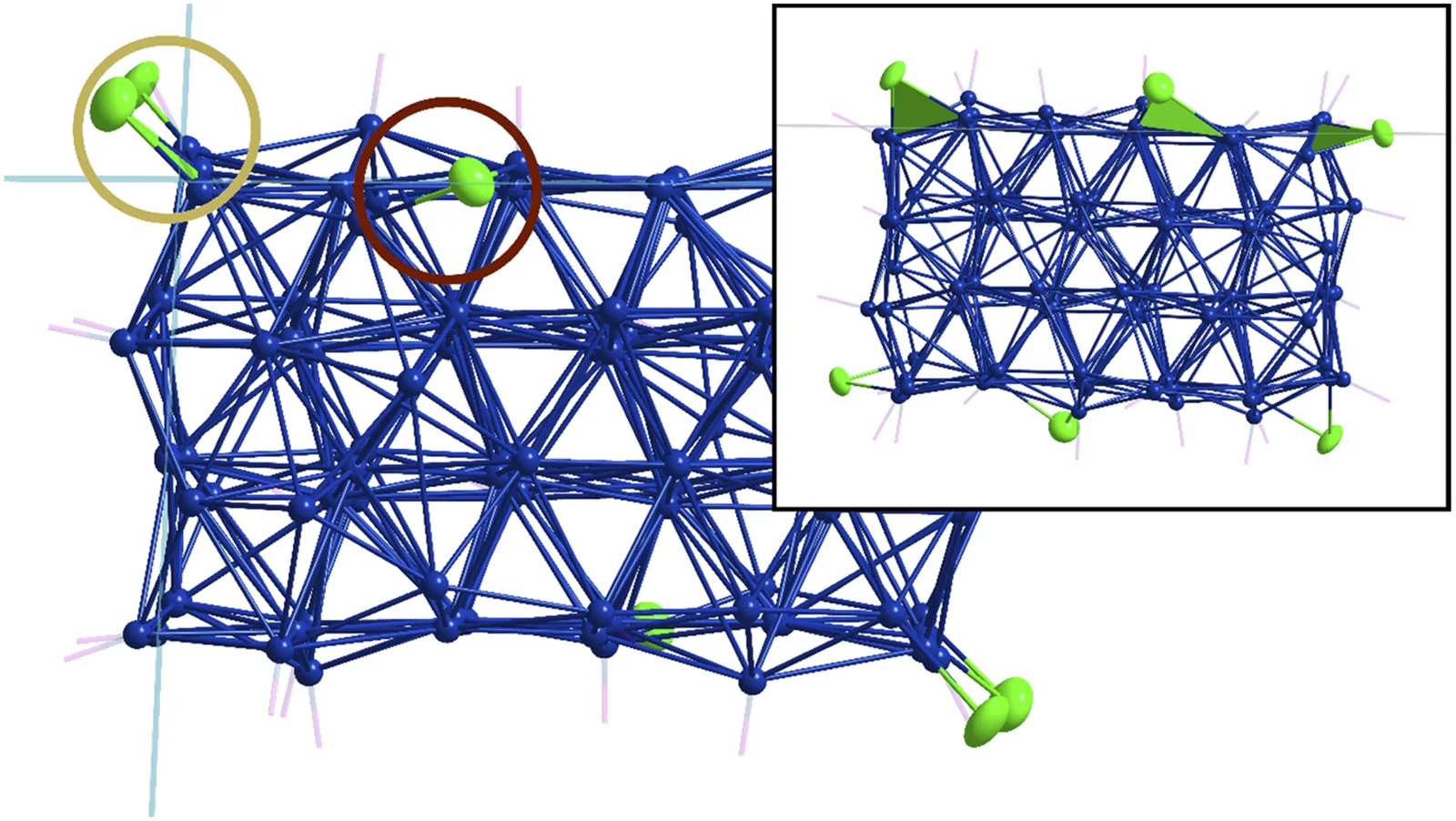
Two planes (cyan) parallel to the corresponding basal and prismatic silver core facets. The planes spanned by the Ag–Cl–Ag units approach the dihedral angle (yellow). One chloride lies within the plane spanned by the outer Ag_32_ layer (red). Inset: view from different angle.

The Ag_128_ kernel is sterically shielded by 28 PPr_3_ ligands (average Ag–P distance: 246.8 pm, Ag_108_: 245.9 pm), which are distributed approximately uniformly over the cluster surface. Coordination is completed by six µ^2^-bridging chlorides located at the edges of the silver core. As the latter is true for Ag_108_ as well, we were interested in how these two systems compare in this regard. The average Ag–Cl bond length in Ag_128_ is 262.9 pm (Ag_108_: 260.6 pm). All chlorides form one longer (average: 273.2 pm) and one shorter (average: 252.6 pm) Ag–Cl contact. The average Ag–Cl–Ag angle is 74.0° (Ag_108_: 70.3°). Averaging a plane over the 32 silver atoms of the outer kernel layer, one can determine the angle between this plane and the one spanned by the Ag–Cl–Ag moieties (Fig. 5). For Ag_108_, this averages to 47.7° with only slight deviations, *i.e.* the chlorides lie near the bisector of the dihedral angle defined by the cluster core facets. For two of the three symmetry-independent chlorides in Ag_128_, this holds true as well (41.9° and 48.3°). In contrast, the third chloride lies almost within the plane spanned by the Ag_32_ sheet, resulting in an angle near 0°. The reason for this difference might be the increased steric demand of PPr_3_ with respect to PEt_3_, as can be seen from the space-filling model (Fig. S11–S13).

### The molecular structure of Ag_132_ and Ag_146_

**Fig. 6:**
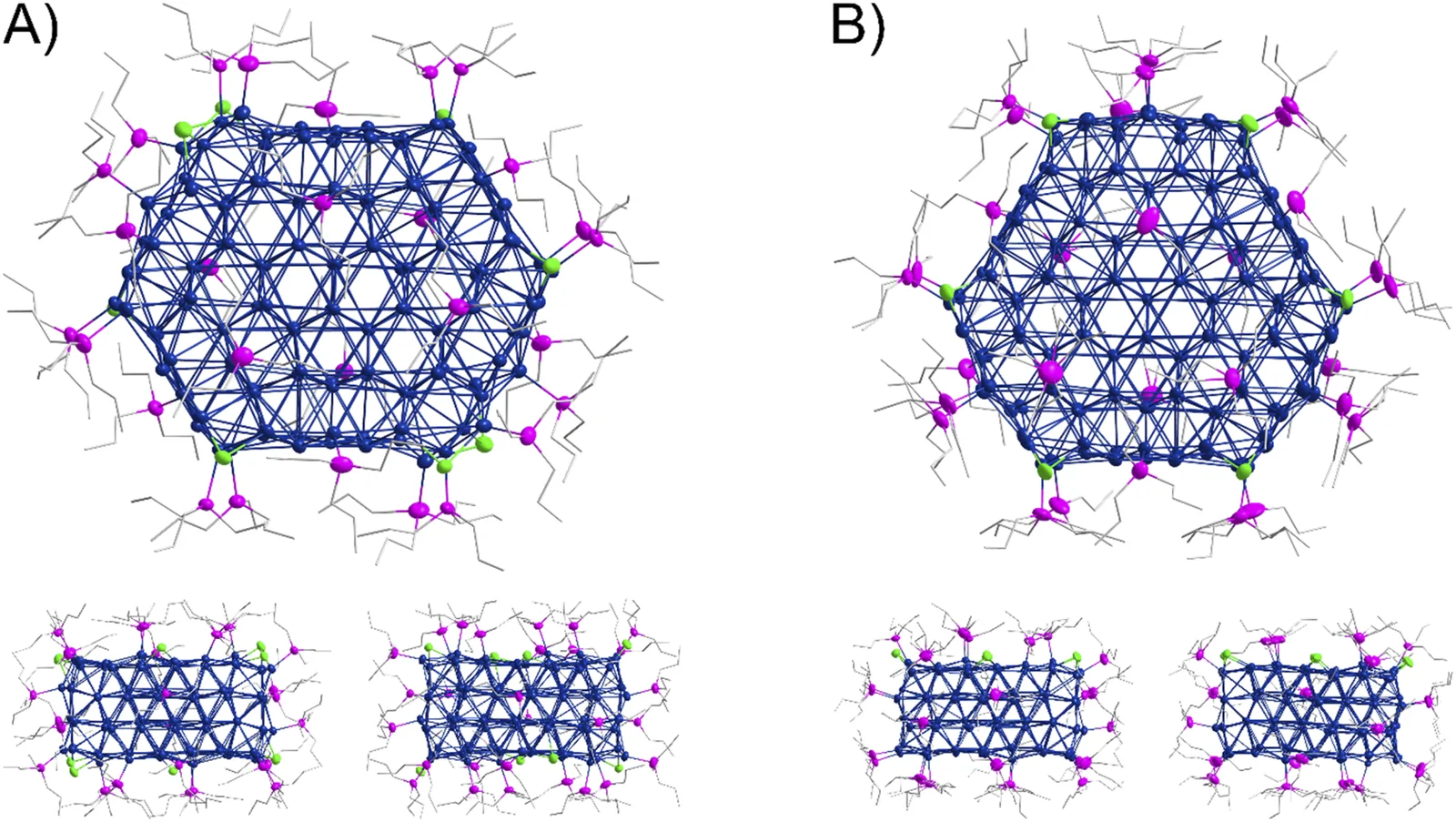
Molecular structures of (A) Ag_132_ and (B) Ag_146_. Ag (blue), P (pink), and Cl (green); thermal ellipsoids are set at 50% probability. C atoms (grey) are shown as wireframe model. H atoms are omitted for clarity.

Since the structure of Ag_160_ obtained by SCXRD is only rudimentary, further analysis is limited to Ag_132_ and Ag_146_ (Fig. 6). Still, the quality of diffraction data worsens with growing cluster size. For Ag_146_, the refined structure is only useful for a connectivity discussion within the cluster and the directly bound P and Cl atoms. A preliminary analysis (*i.e.* connectivity) of Ag_160_ can be found in the SI.

**Fig. 7:**
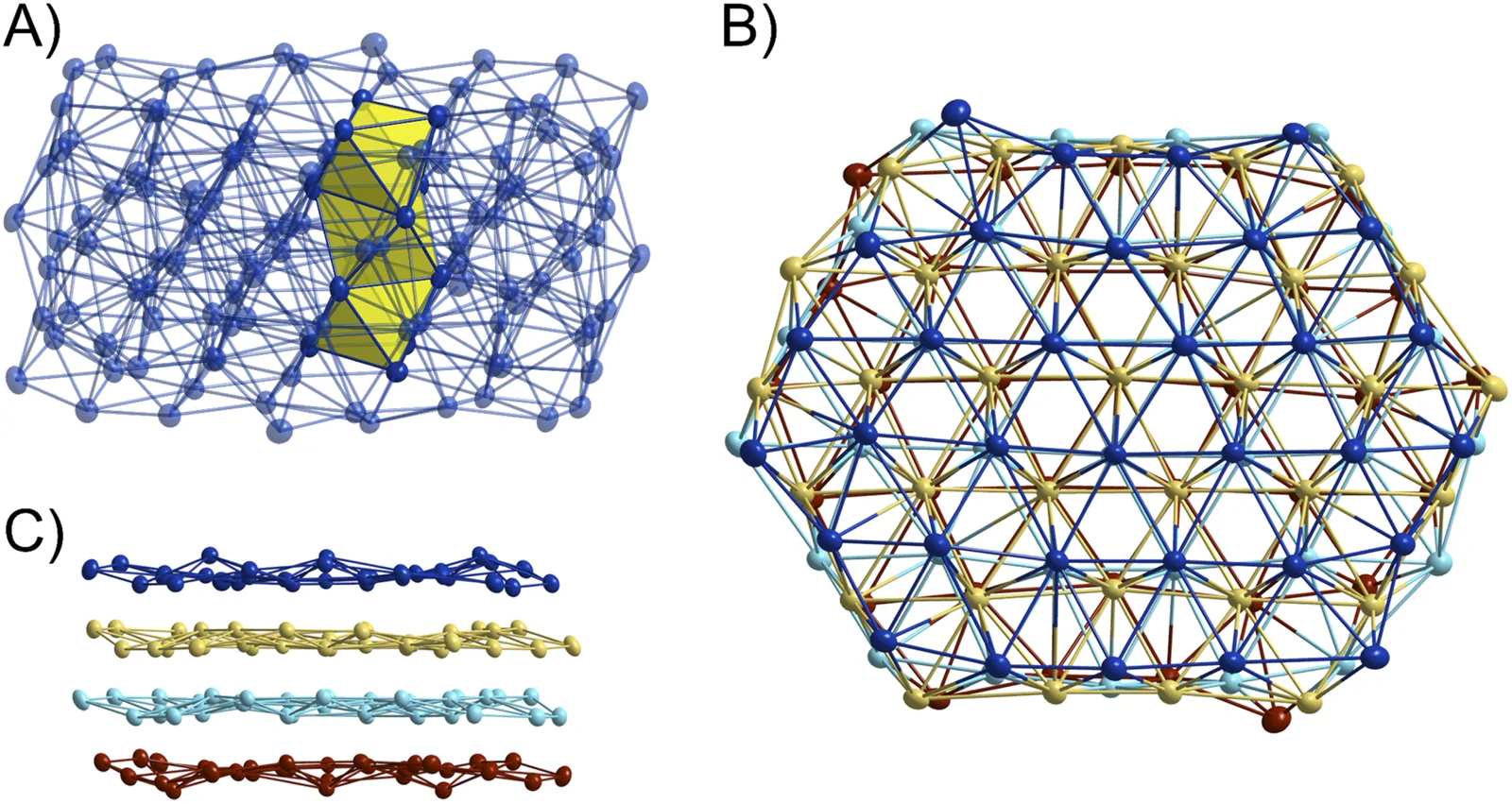
(A) Example of face-sharing octahedra found within Ag_132_. (B) Top and side view of the Ag_132_ core. The atom positions of the first (blue) and third (yellow) as well as those of the second (cyan) and fourth (red) layers are near-congruent, resulting in an ABAB-like stacking. (C) The outer Ag_33_ layers are highly undulated, while for the inner ones undulation is significantly less pronounced.

Ag_132_(PPr_3_)_28_Cl_8_ crystallizes in the triclinic space group *P*1̄ with one molecule in the asymmetric unit. Like its smaller congener, Ag_132_'s core adopts the shape of a flat, irregular, concave hexagonal prism of *C*_i_ symmetry. The core measures 1.9 nm in length, 1.3 nm in width and 0.76 nm in height with edge lengths of the prism of 1.0 nm, 0.9 nm and 0.8 nm respectively. Within the cluster core, a distorted hcp-like structure is found, indicated by face-sharing Ag_6_ octahedra, ABAB stacking and a *c*/*a* ratio of 1.624 (Fig. 7). Continuous shape measure analysis suggests a similar degree of distortion for the Ag_6_ octahedra ensemble, where the biggest outliers again reside on the cluster edges (Fig. S6). Ag_132_ features a layered structure, consisting of four Ag_33_ sheets. The overall mean Ag–Ag distance is 307.0 pm for Ag_132_ and 307.7 pm for Ag_128_. Their intralayer averages are very similar, 307.0 pm and 306.8 pm respectively, whereas the shorter interlayer average in Ag_132_, 306.1 pm *versus* 308.4 pm, is consistent with its slightly smaller total average. Furthermore, the undulation within Ag_132_ is less pronounced: both out-of-plane values as well as mean shape measures are consistently smaller than for Ag_128_.

**Fig. 8:**
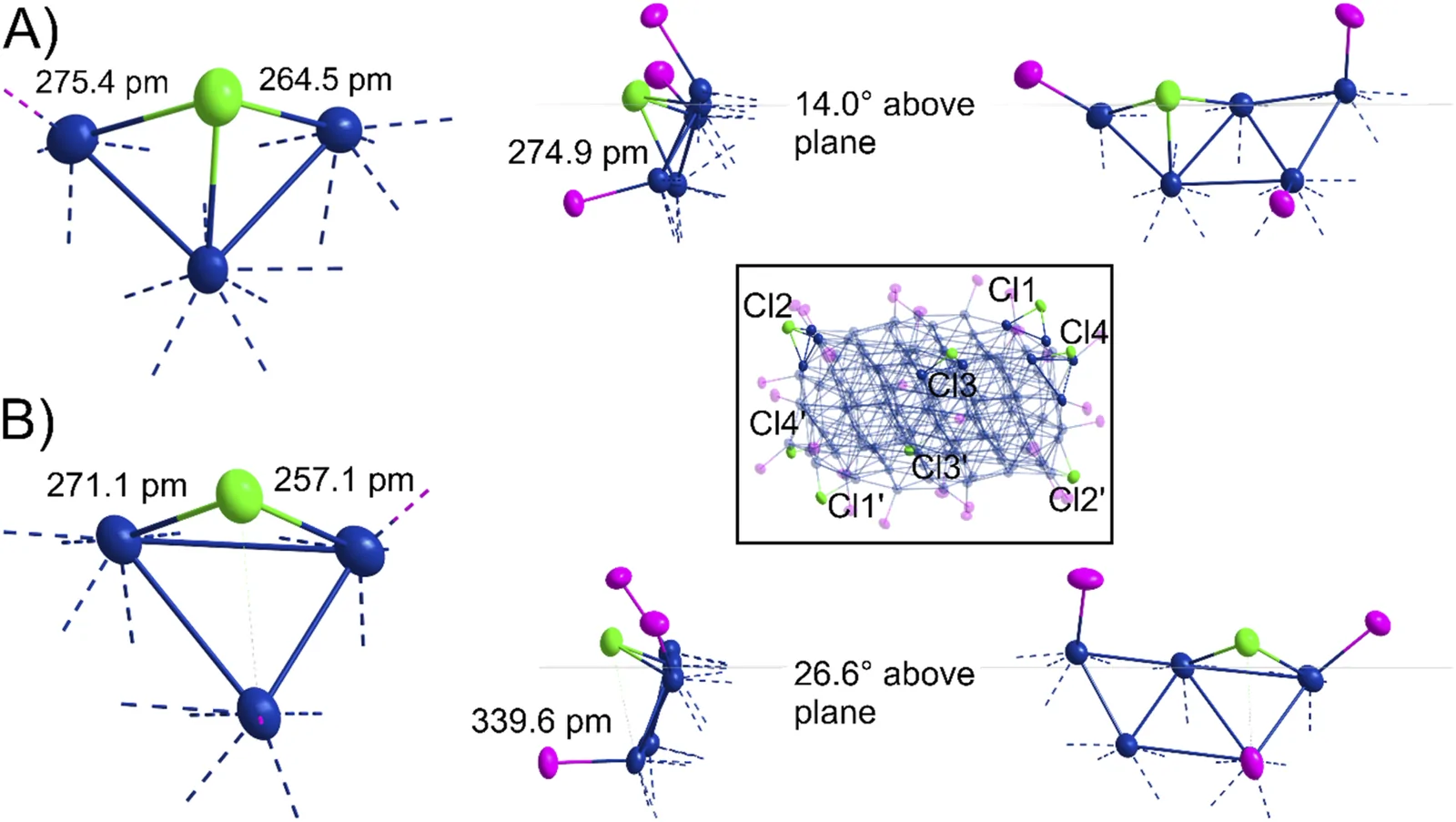
Chlorides Cl1 and Cl3 are located near the dihedral angle spanned by the facets, while the Cl2 and Cl4 Ag–Cl–Ag moieties realize a markedly smaller angle. (A) Close-up of the Cl2–Ag interactions. (B) Close-up of the Cl4–Ag interactions. Inset: overview. Ag (blue), P (pink), and Cl (green).

For the chloride substituents in Ag_132_, a different situation can be found compared to its smaller congeners. Two of the symmetry-independent Ag–Cl–Ag moieties behave as expected (Fig. 8). However, in the case of Cl2, a rather small dihedral angle of 14.0° is found, combined with a Ag–Cl–Ag angle of 96.4°. This behavior might be explained by the additional Ag–Cl contact of 274.9 pm to a silver atom of the inner layer, leading to a µ^3^-coordination of the chloride (Fig. 8A). As expected, the threefold coordination leads to a larger average Ag–Cl distance of 272.2 pm for this chloride. Considering the µ^2^- and µ^3^-coordinations as two opposing extremes, chloride Cl4 can be seen as an intermediate: featuring a dihedral angle of 26.6°, an above-average mean Ag–Cl bond length of 264.1 pm and a large Ag–Cl–Ag angle (86.5°), its situation lies in between the strictly µ^2^- and µ^3^-bridging chlorides. This is corroborated by the Ag(inner)—Cl4 distance of 339.6 pm (Fig. 8B). It is 8 pm smaller than the sum of van der Waals radii (347 pm). While unlikely, this suggests the possibility of a weak interaction.

**Fig. 9:**
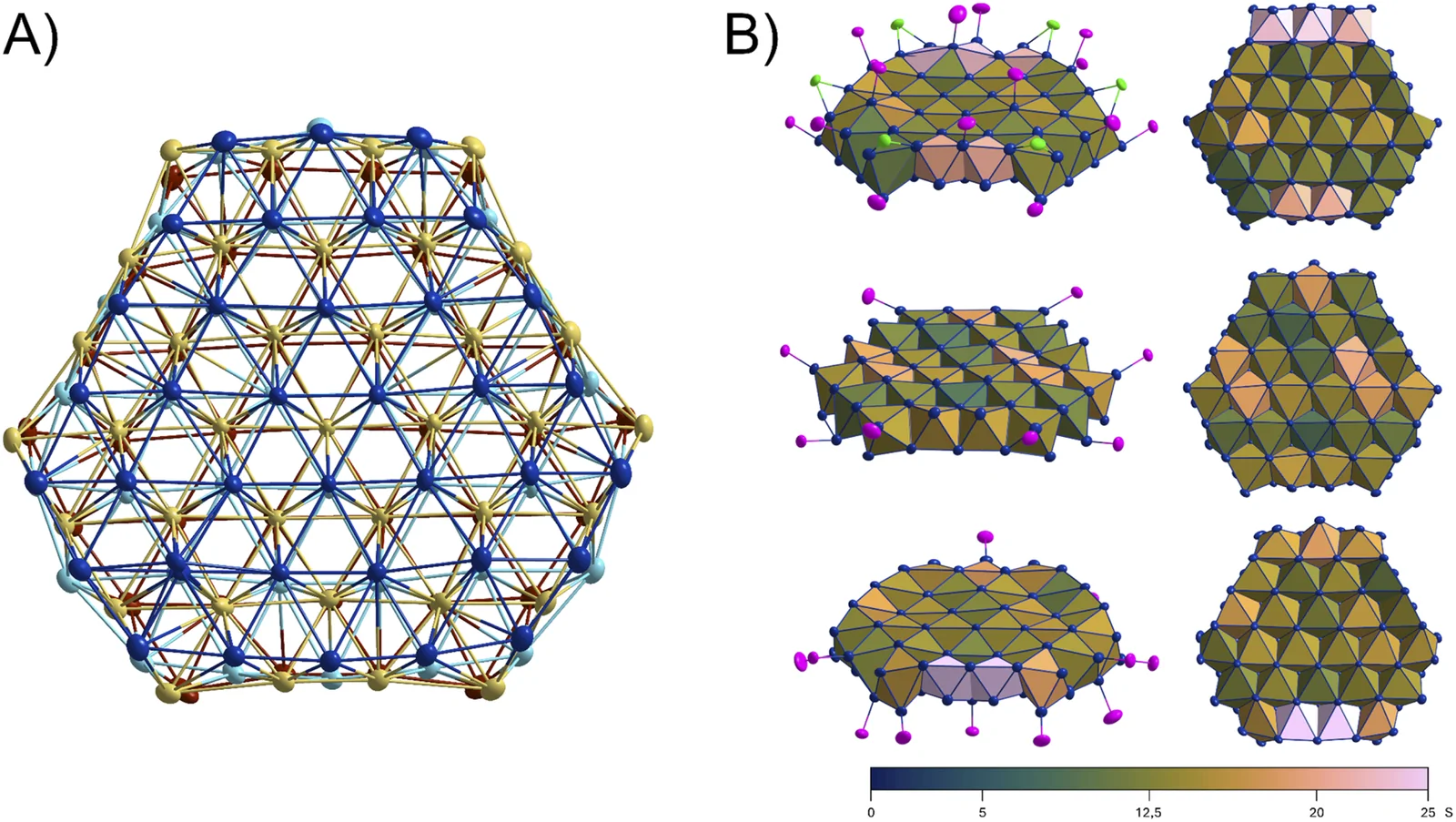
(A) Top view of the Ag_146_ core. The atom positions of the first (blue) and third (yellow) as well as those of the second (cyan) and fourth (red) layers are near-congruent, resulting in an ABAB-like stacking. (B) Heatmap of calculated continuous shape measures *S*. A lower *S* suggests a geometry closer to an ideal octahedron.

Ag_146_(PPr_3_)_30_Cl_6_ (Ag_146_) also crystallizes in the triclinic space group *P*1̄ with two molecules in the asymmetric unit. Structure refinement shows that the metal core structure is mainly consistent with the preceding molecules. The silver core measures 1.6 nm side to side, 1.9 nm edge to edge and around 0.76 nm in height. The hexagonal-prismatic kernel's side lengths alternate between an average of 0.9 nm and 1.0 nm respectively. Like its smaller congeners, Ag_146_ consists of four Ag layers arranged in a distorted hcp-like manner (Fig. 9A). With an average Ag–Ag bond length of 308.8 pm, the cluster follows the trend observed so far. Besides the many similarities with its smaller congeners, this C_1_-symmetric cluster also features structural peculiarities that make Ag_146_ stand out. First, the calculated shape measures are markedly larger than for any other cluster kernel mentioned so far (Fig. 9B). This is noteworthy because this appears to contradict the notion that larger metal kernels lead to more uniform octahedra. However, since the size differences are small, this might just be caused by the asymmetric nature of Ag_146_. Another factor to consider is the decreasing XRD data quality.

Second, in the case of Ag_146_ the six chlorides lie on a single hemisphere of the kernel. However, they are still located at the edges between the large (0001) facet and the neighboring prismatic sides. This also leads to an asymmetric distribution of the phosphines, with three additional phosphines on the chloride-free half (Fig. 6). These differences show that seemingly small changes in composition can lead to stark differences in the arrangement of ligands and substituents.

### Structural relationship between the title compounds, Ag_64_ and Ag_108_

**Fig. 10:**
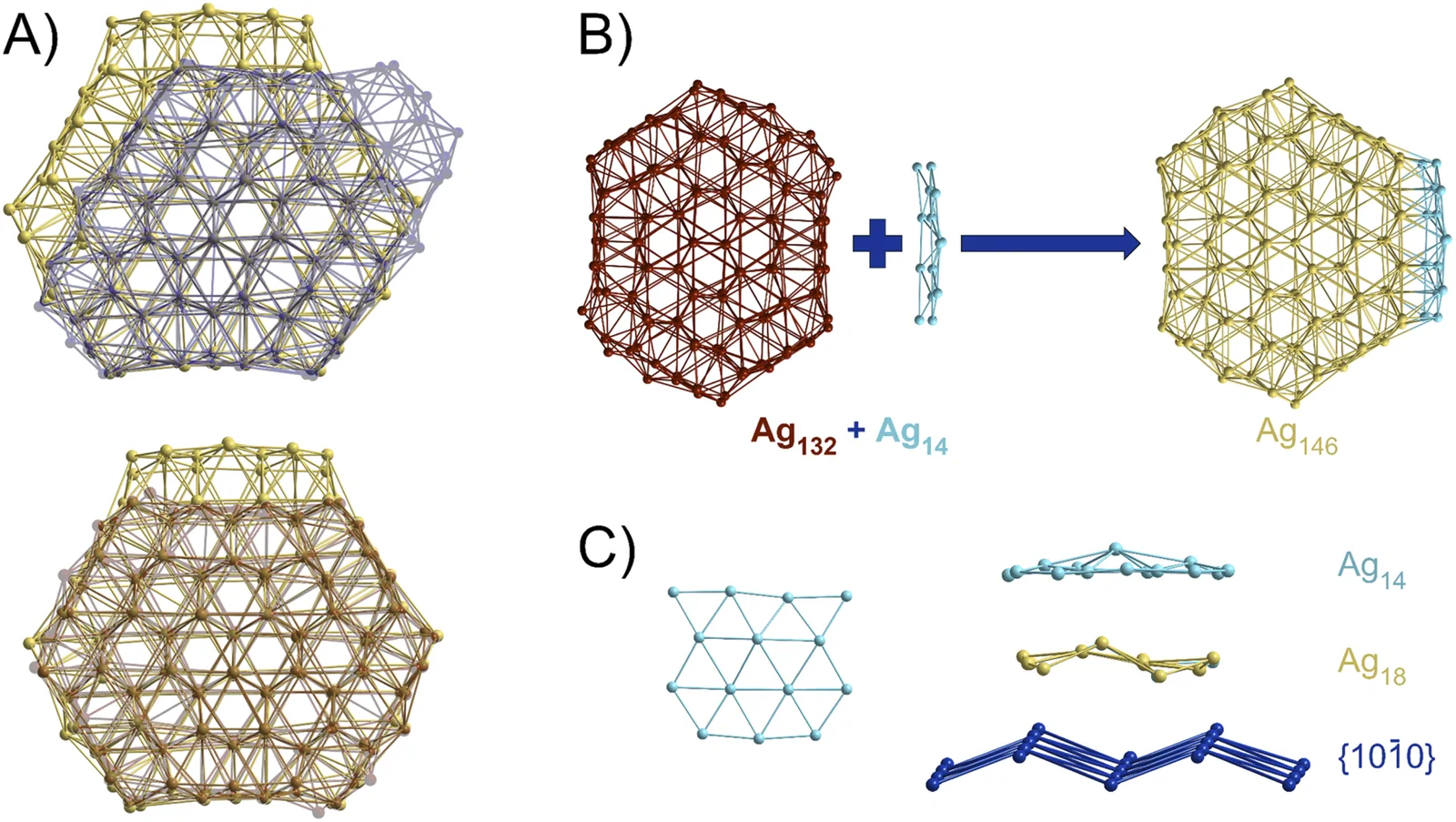
(A) Superimposition of Ag_128_ (blue; top) and Ag_132_ (red; bottom) with Ag_146_ (yellow). (B) The silver cores of Ag_132_ and Ag_146_ differ by a singular Ag_14_ sheet. (C) Frontal view of the Ag_14_ sheet (left). Comparison between Ag_14_ (right, top) and a neighboring Ag_18_ sheet (right, middle). The latter shows a zig-zag surface similar to a {101̄0} facet (right, bottom).

In our 2024 study,^21^ we suggested that Ag_64_ and Ag_108_ might relate to each other *via* a possible growth mechanism. This is even more true for the triad Ag_128_, Ag_132_ and Ag_146_ (and even Ag_160_). As can be seen in Fig. 10A, the silver positions of all three clusters can be neatly superimposed with good agreement, especially for the inner Ag atoms. This is not surprising, since all three silver cores can be described as distorted snippets of a hcp lattice, whereby the degree of metal-like structural regularity mostly correlates with cluster size. When superimposed, several cluster facets coincide with each other. In fact, they do differ by completely filled (prismatic) layers of Ag atoms; *e.g.*Ag_132_ and Ag_146_ differ by one Ag_14_ layer (Fig. 10B). For an animated comparison, see the SI. Interestingly, Ag_108_ does not fit as cleanly into this series (Fig. S15). This might suggest that the Ag_108_ core motif is somehow less related to the title compounds. Yet, the reason might also be as trivial as the fact that different phosphines are present respectively, with different steric bulk and electronics leading to slight structural differences.

Since the title compounds are synthesized from near-identical conditions, the thermodynamic and/or kinetic conditions for their respective formation must lie energetically close together. While it seems possible that the formation of these compounds follows different reaction paths, it is more likely that the compounds are formed *via* a similar pathway. In fact, the structural relationships within the series (Ag_108_), Ag_128_, Ag_132_, Ag_146_ (and Ag_160_) are consistent with discrete stages along a common or partly shared faceted growth pathway. Accordingly, this series provides, to our knowledge, the first experimentally grounded, atomically resolved, step-by-step model of faceted nanocluster growth. This structural model does not imply direct interconversion between the isolated clusters. Faceted growth is a highly discussed topic for nanoparticles yet hindered by their non-atom-precise nature and the “low resolution limit” of HR-TEM experiments. This further elevates the importance of single crystal diffraction experiments.^37^

However, we do not know why hcp-like silver atom arrangements within the cluster core and faceted growth are realized in the present system. DFT calculations suggest that electronics do not play a major role (*vide infra*). Hence, a different explanation is needed. For larger hcp nanoparticles, Wulff construction predicts lowest-energy {0001} planes close together, resulting in hexagonal plates, with low energy {101̄0}-facets as its sides.^38^ This has been experimentally verified for Mg nanoplates.^39^ In our title compounds, some of the sides of the prisms can be seen as {101̄0}-facets as well (Fig. 10C). However, the Wulff construction is not fully applicable to metalloid clusters. Due to their small size in comparison to nanoparticles, one cannot neglect several factors such as edge and vertex energies or reconstruction. The occurrence of nanoplates for fcc metals can be rationalized using a kinetic modified Wulff construction.^40^ Here, instead of the thermodynamic equilibrium of free surface energies, growth kinetics are the shape-determining factor. Norris and co-workers argue that the speed of growth strongly correlates with facet size. According to them, growth on small facets can be orders of magnitude faster due to lower activation barriers for development of new layers, *i.e.* island nucleation. Since the growth of such islands does not create additional, energetically expensive step edges, the remaining facet is quickly filled, leading to a layer-by-layer growth. Yet, their theory on kinetic growth instability assumes island nucleation to be the rate-determining step.^41^

Finally, the role of the ligands must be investigated. It is known that there are substantial differences in reaction rate and capping agent binding capability for different facets, *e.g.* Ag{100} and Ag{111}. By reducing the surface energy or slowing the addition of metal atoms, growth can be inhibited for specific facets. This can be used to synthesize nanoparticles of varying shape, including anisotropic ones such as nanoplates.^7*a*,23,42^ In particular, the influence of halides on the growth of anisotropic nanoparticles has been widely discussed, yet the relevant mechanism (or rather mechanisms) are multifaceted.^43^ For example, it has been shown that the addition of Cl^−^ ions increases the activity for oxidative etching reactions.^44^ Selective binding on certain crystal facets^45^ and changes in precursor speciation, availability, and reduction kinetics^43*b*,46^ are also frequently discussed. As reported elsewhere, chlorides may also play an important role in the stabilization of nanoclusters.^28*b*,47^

As for the title compounds, the phosphines are approximately equally distributed around the cluster surface. While undeniably important for the electronic stabilization and steric shielding, no remarkable anisotropic distribution is apparent. On the other hand, the chloride ligands occupy special positions within the clusters: without exception, all chlorides are located at edges between basal and prismatic facets. This suggests a specific stabilization of the basal-prismatic junctions, as silver-chloride coordination compensates for the low valency of the vertex atoms. The preference for lateral growth over the addition of a fifth basal layer might be based on this effect. Other effects of chloride, including precursor speciation, oxidative etching, and kinetic trapping, cannot be distinguished using the present data. Even though we cannot differentiate between different modes of action, chlorides are plausible contributors to the observed faceted growth.

Another metalloid silver cluster Ag_64_(PPr_3_)_16_Br_6,_ which will be discussed in detail in a forthcoming work, constitutes compelling evidence of this hypothesis: while the reduction of (Pr_3_P)AgCl leads to the isolable products Ag_128_ to Ag_160_, the reduction of (Pr_3_P)AgBr leads to a cluster isostructural to Ag_64_. Since cluster formation and growth are highly complex processes, we deem it plausible that many different species are present within a singular reaction mixture. Still, neither a Ag_64_ analogue has been isolated for the chloride reactions nor higher congeners for the bromide ones. This leads us to the conclusion that chlorides probably are an integral part of lateral growth within our system.

### Preliminary observations from cluster thermolysis

While rational design of nanoparticles^48^ and metalloid clusters^49^ is widely discussed in the literature, high inherent complexity and, in the case of nanoparticles, the lack of atom-precise composition preclude a definitive general approach.^50^ This is also true for the vast field of seed-mediated syntheses.^6,51^ General trends have been elucidated, yet the nature of the seed species often remains unknown.^5*b*,52^

In comparison, evidence for cluster growth without significant rearrangement of the core or exchange of ligands is rather scarce. For example, Zeng *et al.* report on the metalloid clusters Au_28_(TBBT)_20_, Au_36_(TBBT)_24_, Au_44_(TBBT)_28_ and Au_52_(TBBT)_32_ (TBBT = –SC_6_H_4_-4-^*t*^Bu) forming a magic series that results from the anisotropic addition of two interpenetrating cuboctahedra to the preceding cluster.^53^ Yet, Au_28_ and Au_36_ are synthesized *via* ligand-exchange-induced structure transformation (LEIST)^26*a*,54^ from Au_25_(PET)_18_ and Au_38_(PET)_24_ (PET = –SC_2_H_4_Ph) respectively, with the former undergoing drastic structural changes from icosahedral^55^ to cuboctahedral as well. On the other hand, both Au_44_ and Au_52_ can be synthesized from HAuCl_4_*via* near-identical conditions. A prolonged reaction at elevated temperatures with excess of thiol represents the last step of the respective syntheses. These harsh conditions are known to induce size-focusing *via* etching of the metal core.^56^ Meanwhile, Xie and co-workers were able to identify 35 different species involved in the growth of [Au_25_(*p*-MBA)_18_]^−^ into [Au_44_(*p*-MBA)_26_]^2−^ (*p*-MBA = –SC_6_H_4_-4-COOH), elucidating the complexity involved in cluster reactions.^57^ Further analysis was hindered by the lack of crystal structures, though.

While atomic precision definitely helped gain interesting and important insights, the mentioned examples might not reproduce the mechanisms present in the growth of primary clusters. The title compounds Ag_128_, Ag_132_, Ag_146_ and Ag_160_ might help fill this gap due to several reasons: (a) there is no apparent rearrangement within the metal cores, (b) the ligands used do not change, (c) the isolable products are pure and yields are high, (d) the structures of several congeners have been elucidated *via* SCXRD and (e) differ only by fully filled Ag sheets. The structural relationships are consistent with a possible stepwise lateral-growth sequence, although the isolated structures do not establish an actual kinetic pathway or direct interconversion.

**Fig. 11:**
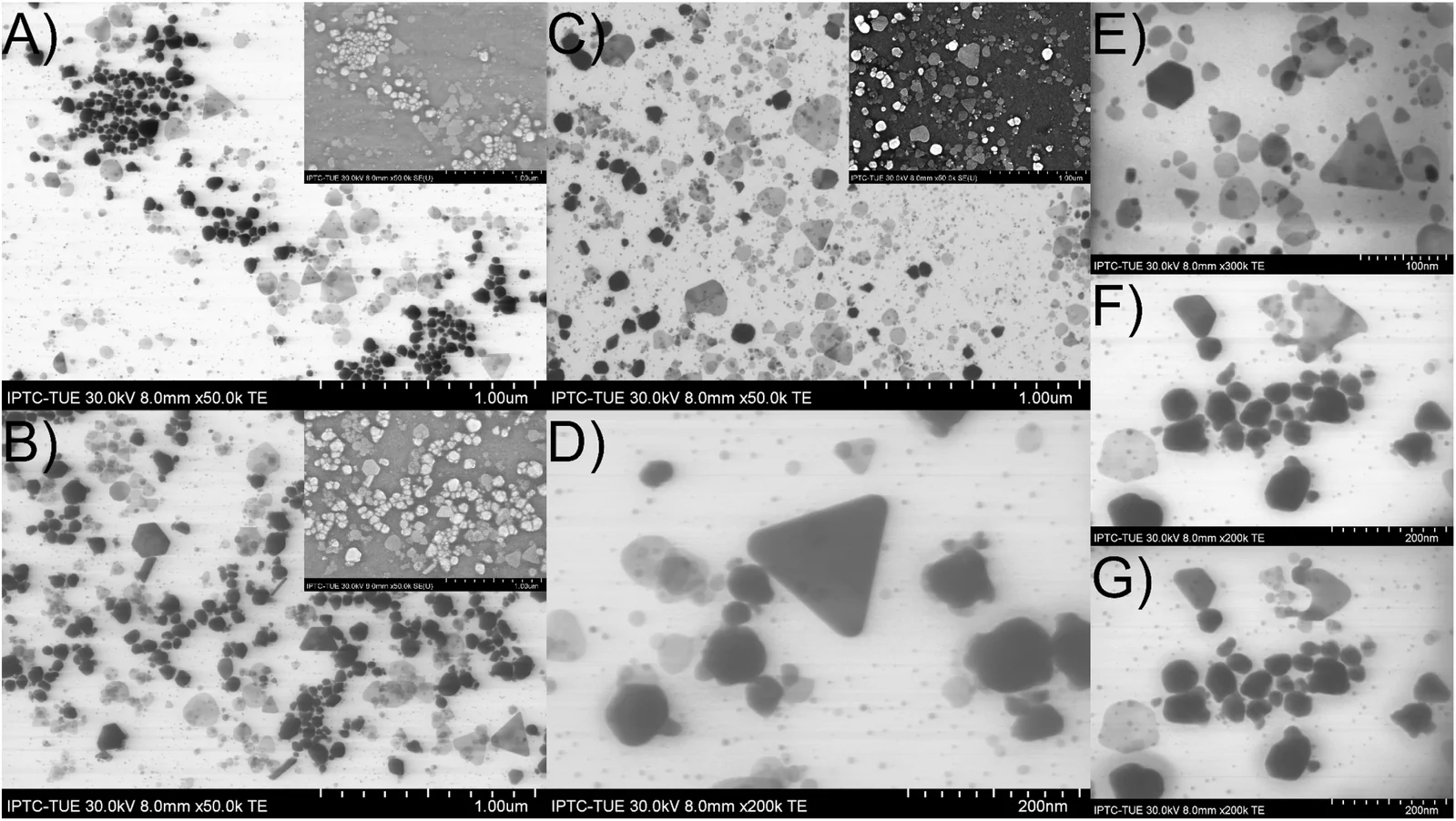
SEM images of decomposed Ag_128_ samples. (A–C) Overview of different samples and areas. Trigonal, truncated-trigonal and hexagonal nanosheets coexist with undefined other decomposition products. Insets: secondary electron images. (D) A rather large trigonal nanoprism shows curvature around its vertices. (E) Hexagonal and trigonal nanosheets coexist, with no correlation between size and shape. (F and G) The electron beam melts some nanosheets. This does not always happen but only to some particles, with no indication for which particle is affected.

In their 2022 work, Song *et al.* proposed the trigonal-prismatic cluster [Au_56_(SC_6_H_4_-4-^*t*^Bu)_24_(P(C_6_H_4_-4-X)_3_)_6_Br_2_]^2+^ (X = CF_3_, Cl, F, H) to be a model seed for the growth of gold nanoprisms.^58^ As far as we are aware, there are no data on the actual usage of Au_56_ in seed-mediated experiments though. Until this point, our hypothesis of cluster growth relied solely on the structural relationship between the title compound structures. To further test this hypothesis, we wanted to use Ag_128_ in the preparation of silver nanoparticles. While this would not constitute a definitive proof of cluster growth, the formation of nanoplates would demonstrate that faceted growth is possible in our system at all. One possible method of nanoparticle synthesis is thermolysis.^59^ In this context, we were able to exploit Ag_128_'s low thermal stability, eliminating the need for additional heating. At temperatures above −30 °C, within a few hours the dark red solutions of Ag_128_ become almost colorless while dark grey precipitate forms. As this is often accompanied by a silver mirror and the formation of insoluble precipitate, we usually discard these reaction mixtures. Our first attempt at analyzing the remaining solids, *i.e.* letting a cluster solution decompose, drying the solids and further analyzing this sample *via* SEM, proved unfruitful. Therefore, we prepared SEM samples by directly drop-casting a cold Ag_128_ solution onto copper grids, either slowly warming up under pseudo-inert or ambient conditions. Indeed, we were able to confirm the formation of nanosheets by SEM (Fig. 11). Trigonal, truncated trigonal and hexagonal nanosheets of varying size and thickness are present within the samples, with no apparent correlation between shape and size. Overall, the largest nanoparticles we found measured around 250 nm. Remarkably, the general shape of the parent cluster, *i.e.* flat (hexagonal) prismatic, can be found within the resulting nanoparticles. While this is no definitive proof for seeded growth of clusters to nanoparticles, this still might help our understanding of how the shape of nanoclusters might influence the resulting nanoparticle shape.

Furthermore, we repeated this experiment several times with different compounds. As expected, both Ag_108_ and Ag_132_ yielded nanoprisms as well, whereby the former sample also consisted of other faceted nanoparticles. For Ag_64_ and the precursor (Pr_3_P)AgCl, we did not find proof of nanoparticle formation. For further results and analysis of the targeted decomposition experiments, see the chapter on SEM and EDX measurements within the SI. As this is a proof-of-concept experiment and the formation/design of nanoparticles lies beyond the scope of this work, no further experiments were conducted regarding procedure optimization.

### DFT calculations

Density functional theory (DFT) calculations were used to study atomic and electronic structures and the stability of the synthesized protected cluster series Ag_108_, Ag_128_, Ag_132_, Ag_146_ and Ag_160_. The starting structures were taken directly from experiments in the case of the three smallest clusters and the partially resolved structures were built to complete model structures in the case of the two largest clusters. In addition to the fully protected clusters, we also analyzed the electronic structure of the bare metal clusters of the same size and symmetry, which were taken from the core structures of each cluster. For pure metal cluster models, the total charges were selected to maintain the same free valence electron count as in the parent protected cluster. Calculations were done using the GPAW DFT code-package^60^ with real-space grids and Perdew–Burke–Ernzerhof exchange–correlation functional.^61^ All structures were first optimized before further analysis.

Structure optimization of the clusters consistently shows a contraction of the cluster core by 2–3% for Ag_108_, Ag_128_, Ag_132_ and Ag_146_, and by 4–5% for Ag_160_ depending on the core dimension. The larger discrepancy in the structural change of Ag_160_ is expected because of the lower quality of the resolved experimental structure and the manually constructed additions to the structure, whereby a tentative ligand-shell model was used. In general, we observe that the undulation of the Ag-layers is also retained in the optimized structures despite the contraction of the core. We can also see that the level of the disorder at the edges due to the ligand binding remains qualitatively the same as seen in the experimental crystal structures.

Electronic structure of the protected metal nanoclusters in the size range of 100–160 atoms has been of great interest in the field to explore the threshold size of transition from molecular to metallic and hence also to plasmonic systems.^62–64^ The cluster series reported in this paper spans the same size range and therefore, analyzing the electronic structure can indicate the degree of metal-like character and whether electronic effects have a role in the stability of the clusters. The HOMO–LUMO energy gaps for the calculated clusters are consistently very small as follows: Ag_108_: 0.12 eV, Ag_128_: 0.03 eV, Ag_132_: 0.03 eV, Ag_146_: 0.10 eV, and Ag_160_: 0.04 eV. This is consistent with emerging metal-like electronic character. Similar low HOMO–LUMO energy gaps (in range 0.03–0.08 eV) were seen for the pure metal cluster models without ligands using the same free valence electron counts Ag_108_: 102 e, Ag_128_: 122 e, Ag_132_: 124 e, Ag_146_: 140 e, Ag_160_: 154 e (orbital plots are found in Fig. S25). The result is reasonable since the only electron withdrawing ligands are the halides which are in minority and therefore the free electron counts are relatively high compared to, for example, similarly sized thiolated metal clusters. Notably, these prism-shaped clusters do not favor any spherical superatom shell structure but have their own adjusted versions of a particle in box-like electronic shell structures.

**Fig. 12:**
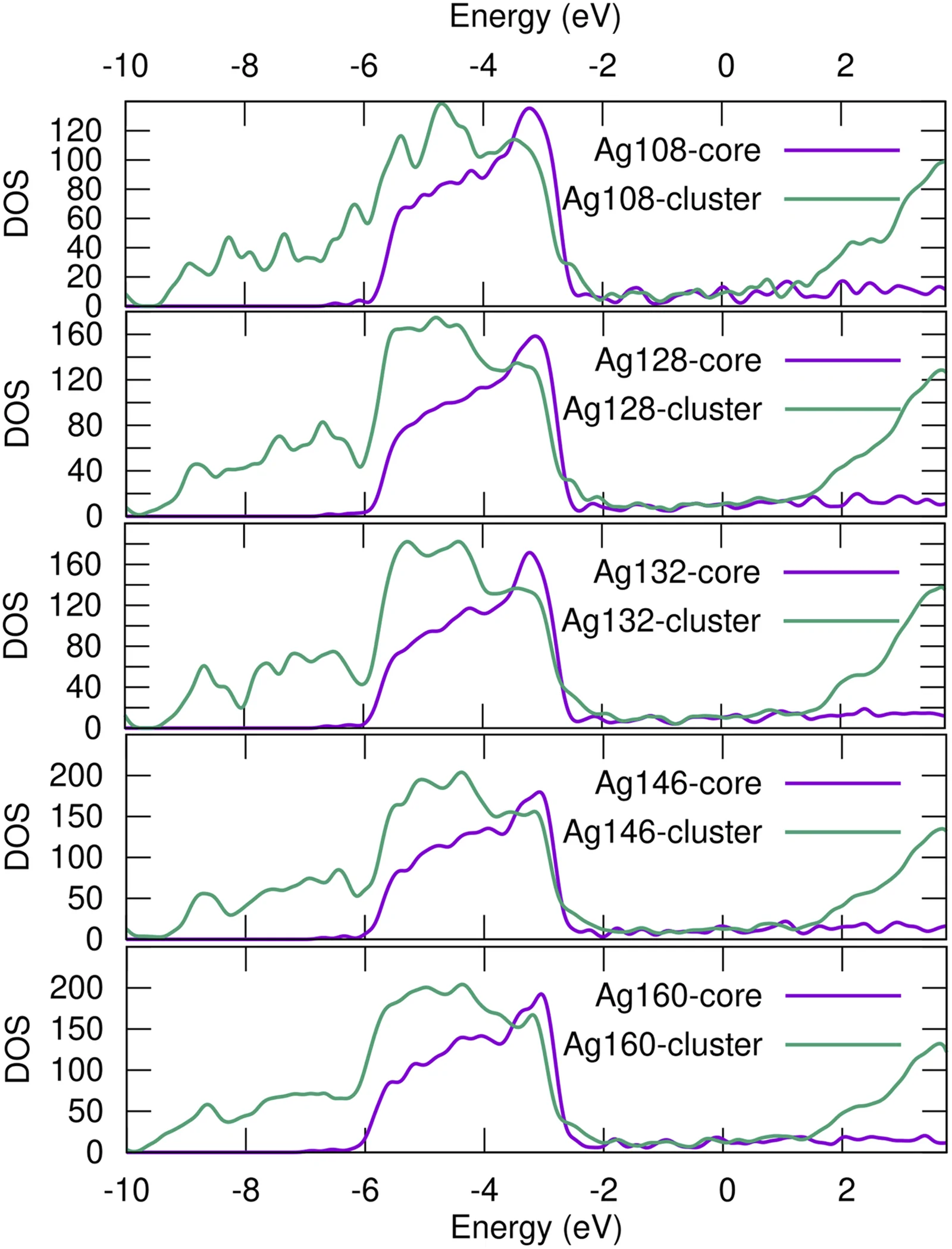
Density of electronic states for the Ag_108_, Ag_128_, Ag_132_, Ag_146_ and Ag_160_ clusters in panels from top to bottom with ligands in green and without ligands in purple. The Fermi level is shifted to 0 eV in all cases.

The electronic density of states (DOS) are shown in Fig. 12 for all the clusters. The shape of the DOS is very similar for all the clusters. The silver d-band is located below −2.5 eV while the band of the unoccupied ligand states can be found above 1.5 eV. The main unoccupied ligand band is missing from the DOS of the pure metal clusters. Most importantly, all five calculated clusters show the same kind of continuous band of states around the Fermi energy at 0 eV for clusters with and without ligands. These are expected to be due to the delocalized states at the metal core formed by the free valence electrons. A general observation is that the states around the Fermi level do not create distinct shells that would be seen as clear gaps in the DOS profile. This confirms that the electronic shell structure is not an important factor for the stability of this cluster series.

## Conclusion

To summarize, we report the synthesis and characterization of four structurally related metalloid silver clusters, Ag_128_, Ag_132_, Ag_146_ and Ag_160_, with distorted hcp-like, hexagonal-prismatic cores. Their structural similarities and similar reaction conditions are consistent with a possible stepwise lateral-growth sequence, although the isolated endpoint structures do not establish a kinetic pathway or direct interconversion.

Chloride ligands occupy basal-prismatic junctions and may contribute to edge stabilization and faceted growth. DFT calculations yield small Kohn–Sham gaps and dense frontier states, consistent with emerging metal-like electronic character. Thermolysis of the title compounds produces Ag-rich trigonal/hexagonal nanoprisms, which hints at seed-mediated growth of the clusters.

Still, this work is mostly descriptive and the actual mechanisms that govern the faceted growth remain unknown. Nevertheless, the presented results indicate that chlorides can be seen as one important factor. We hope that the presented structural results of faceted cluster growth encourage theoretical chemists to further investigate this phenomenon. Elucidation of the reaction mechanism is thereby pivotal for a useful rational design of metalloid clusters and nanoparticles.

## Author contributions

All authors contributed to the manuscript and approved the final version. M. A. Kordan: synthesis, validation, formal analysis, writing – original draft. C. Schrenk: X-ray diffraction analysis. S. Malola: DFT calculations. H. Häkkinen: DFT calculations. A. Schnepf: funding acquisition, project administration, writing – original draft.

## Conflicts of interest

The authors declare no conflict of interest.

## Supplementary Material

SC-OLF-D6SC06118D-s001

SC-OLF-D6SC06118D-s002

SC-OLF-D6SC06118D-s003

## Data Availability

The data supporting the findings of this study are available in the supplementary information (SI) of this article. Supplementary information: experimental procedures, explanation of the undulation and shape measure calculations, crystallographic data, SEM data, and computational data. See DOI: https://doi.org/10.1039/d6sc06118d. CCDC 2562216–2562219 contain the supplementary crystallographic data for this paper.^65*a*–*d*^

## References

[cit1] Sharma R. K., Yadav S., Dutta S., Kale H. B., Warkad I. R., Zbořil R., Varma R. S., Gawande M. B. (2021). Silver nanomaterials: synthesis and (electro/photo) catalytic applications. Chem. Soc. Rev..

[cit2] Cui X., Ruan Q., Zhuo X., Xia X., Hu J., Fu R., Li Y., Wang J., Xu X. (2023). Photothermal Nanomaterials: A Powerful Light-to-Heat Converter. Chem. Rev..

[cit3] Chatterjee S., Lou X.-Y., Liang F., Yang Y.-W. (2022). Surface-functionalized gold and silver nanoparticles for colorimetric and fluorescent sensing of metal ions and biomolecules. Coord. Chem. Rev..

[cit4] Naganthran A., Verasoundarapandian G., Khalid F. E., Masarudin M. J., Zulkharnain A., Nawawi N. M., Karim M., Abdullah C. A. C., Ahmad S. A. (2022). Synthesis, Characterization and Biomedical Application of Silver Nanoparticles. Materials.

[cit5] LaMer V. K., Dinegar R. H. (1950). Theory, Production and Mechanism of Formation of Monodispersed Hydrosols. J. Am. Chem. Soc..

[cit6] Xia Y., Gilroy K. D., Peng H.-C., Xia X. (2017). Seed-Mediated Growth of Colloidal Metal Nanocrystals. Angew. Chem., Int. Ed..

[cit7] Xia Y., Xia X., Peng H.-C. (2015). Shape-Controlled Synthesis of Colloidal Metal Nanocrystals: Thermodynamic versus Kinetic Products. J. Am. Chem. Soc..

[cit8] Purath A., Köppke R., Schnöckel H. (1999). [Al_7_{N(SiMe_3_)_2_}_6_]^−^: A First Step towards Aluminum Metal Formation by Disproportionation. Angew. Chem., Int. Ed..

[cit9] Chakraborty I., Pradeep T. (2017). Atomically Precise Clusters of Noble Metals: Emerging Link between Atoms and Nanoparticles. Chem. Rev..

[cit10] Wilcoxon J. P., Abrams B. L. (2006). Synthesis, structure and properties of metal nanoclusters. Chem. Soc. Rev..

[cit11] Schnepf A., Schnöckel H. (2001). Synthesis and Structure of a Ga_84_R_20_^4−^ Cluster—A Link between Metalloid Clusters and Fullerenes?. Angew. Chem., Int. Ed..

[cit12] Yang H., Yan J., Wang Y., Su H., Gell L., Zhao X., Xu C., Teo B. K., Häkkinen H., Zheng N. (2017). Embryonic Growth of Face-Center-Cubic Silver Nanoclusters Shaped in Nearly Perfect Half-Cubes and Cubes. J. Am. Chem. Soc..

[cit13] Alhilaly M. J., Bootharaju M. S., Joshi C. P., Besong T. M., Emwas A.-H., Juarez-Mosqueda R., Kaappa S., Malola S., Adil K., Shkurenko A., Häkkinen H., Eddaoudi M., Bakr O. M. (2016). [Ag_67_(SPhMe_2_)_32_(PPh_3_)_8_]^3+^: Synthesis, Total Structure, and Optical Properties of a Large Box-Shaped Silver Nanocluster. J. Am. Chem. Soc..

[cit14] Ma X., Bai Y., Song Y., Li Q., Lv Y., Zhang H., Yu H., Zhu M. (2020). Rhombicuboctahedral Ag_100_: Four-Layered Octahedral Silver Nanocluster Adopting the Russian Nesting Doll Model. Angew. Chem., Int. Ed..

[cit15] Yang H., Lei J., Wu B., Wang Y., Zhou M., Xia A., Zheng L., Zheng N. (2013). Crystal structure of a luminescent thiolated Ag nanocluster with an octahedral Ag_6_^4+^ core. Chem. Commun..

[cit16] Yang H., Wang Y., Chen X., Zhao X., Gu L., Huang H., Yan J., Xu C., Li G., Wu J., Edwards A. J., Dittrich B., Tang Z., Wang D., Lehtovaara L., Häkkinen H., Zheng N. (2016). Plasmonic twinned silver nanoparticles with molecular precision. Nat. Commun..

[cit17] Hu F., Yang G., Zheng L.-M., Liang G.-J., Wang Q.-M. (2025). Deciphering icosahedra structural evolution with atomically precise silver nanoclusters. Science.

[cit18] Yuan S.-F., Li P., Tang Q., Wan X.-K., Nan Z.-A., Jiang D.-e., Wang Q.-M. (2017). Alkynyl-protected silver nanoclusters featuring an anticuboctahedral kernel. Nanoscale.

[cit19] Biswas S., Das A. K., Reber A. C., Biswas S., Bhandary S., Kamble V. B., Khanna S. N., Mandal S. (2022). The New Ag–S Cluster [Ag_50_S_13_(S^t^Bu)_20_][CF_3_COO]_4_ with a Unique hcp Ag_14_ Kernel and Ag_36_ Keplerian-Shell-Based Structural Architecture and Its Photoresponsivity. Nano Lett..

[cit20] Diecke M., Schrenk C., Schnepf A. (2020). Synthesis and Characterization of the Highly Unstable Metalloid Cluster Ag_64_(P^n^Bu_3_)_16_Cl_6_. Angew. Chem., Int. Ed..

[cit21] Kordan M. A., Schrenk C., Schnepf A. (2024). Ag_108_(PEt_3_)_24_Cl_6_: A Hexagonal Prismatic Metalloid Cluster. Chem.–Eur. J..

[cit22] Jona F., Marcus P. M. (2004). Metastable phases of silver and gold in hexagonal structure. J. Phys.: Condens. Matter.

[cit23] Tao A. R., Habas S., Yang P. (2008). Shape Control of Colloidal Metal Nanocrystals. Small.

[cit24] Strienz M., Schnepf A. (2024). Au_30_(P^*i*^Pr_2_^*n*^Bu)_12_Cl_6_—An Open Cluster Provides Insight into the Influence of the Sterical Demand of the Phosphine Ligand in the Formation of Metalloid Gold Clusters. Molecules.

[cit25] Yan J., Teo B. K., Zheng N. (2018). Surface Chemistry of Atomically Precise Coinage–Metal Nanoclusters: From Structural Control to Surface Reactivity and Catalysis. Acc. Chem. Res..

[cit26] Zeng C., Chen Y., Das A., Jin R. (2015). Transformation Chemistry of Gold Nanoclusters: From One Stable Size to Another. J. Phys. Chem. Lett..

[cit27] Tolman C. A. (1977). Steric Effects of Phosphorus Ligands in Organometallic Chemistry and Homogeneous Catalysis. Chem. Rev..

[cit28] Zhang W.-J., Liu Z., Song K.-P., Aikens C. M., Zhang S.-S., Wang Z., Tung C.-H., Sun D. (2021). A 34-Electron Superatom Ag_78_ Cluster with Regioselective Ternary Ligands Shells and Its 2D Rhombic Superlattice Assembly. Angew. Chem., Int. Ed..

[cit29] Hu F., He R.-L., Guan Z.-J., Liu C.-Y., Wang Q.-M. (2023). Disc-Like Silver Nanocluster Ag_93_ Built with Bicapped Hexagonal Prismatic Ag_15_ and Ino Decahedral Ag_13_ Units. Angew. Chem., Int. Ed..

[cit30] An J., Tang B., Ning X., Zhou J., Xu S., Zhao B., Xu W., Corredor C., Lombardi J. R. (2007). Photoinduced Shape Evolution: From Triangular to Hexagonal Silver Nanoplates. J. Phys. Chem. C.

[cit31] Scarabelli L., Sun M., Zhuo X., Yoo S., Millstone J. E., Jones M. R., Liz-Marzán L. M. (2023). Plate-Like Colloidal Metal Nanoparticles. Chem. Rev..

[cit32] Pinsky M., Avnir D. (1998). Continuous Symmetry Measures. 5. The Classical Polyhedra. Inorg. Chem..

[cit33] Llunell M. , Casanova D., Cirera J., Alemany P. and Alvarez S., SHAPE. Program for the Stereochemical Analysis of Molecular Fragments by Means of Continuous Shape Measures and Associated Tools, Version 2.1, Universitat de Barcelona, Barcelona, 2013

[cit34] Alvarez S., Avnir D., Llunell M., Pinsky M. (2002). Continuous symmetry maps and shape classification. The case of six-coordinated metal compounds. New J. Chem..

[cit35] Holleman A. F. , Wiberg E. and Wiberg N., Lehrbuch der Anorganischen Chemie, de Gruyter, Berlin, 103rd edn, 2017, pp. 1722–1725

[cit36] Chadi D. J. (1979). Atomic and Electronic Structures of Reconstructed Si(100) Surfaces. Phys. Rev. Lett..

[cit37] Li Y., Jin R. (2020). Seeing Ligands on Nanoclusters and in Their Assemblies by X-ray Crystallography: Atomically Precise Nanochemistry and Beyond. J. Am. Chem. Soc..

[cit38] Wulff G. (1901). XXV. Zur Frage der Geschwindigkeit des Wachsthums und der Auflösung der Krystallflächen. Z. Kristallogr. - Cryst. Mater..

[cit39] Ringe E. (2020). Shapes, Plasmonic Properties, and Reactivity of Magnesium Nanoparticles. J. Phys. Chem. C.

[cit40] Ringe E., Van Duyne R. P., Marks L. D. (2013). Kinetic and Thermodynamic Modified Wulff Constructions for Twinned Nanoparticles. J. Phys. Chem. C.

[cit41] Riedinger A., Ott F. D., Mule A., Mazzotti S., Knüsel P. N., Kress S. J. P., Prins F., Erwin S. C., Norris D. J. (2017). An intrinsic growth instability in isotropic materials leads to quasi-two-dimensional nanoplatelets. Nat. Mater..

[cit42] Sun Y., Mayers B., Herricks T., Xia Y. (2003). Polyol Synthesis of Uniform Silver Nanowires: A Plausible Growth Mechanism and the Supporting Evidence. Nano Lett..

[cit43] Lohse S. E., Burrows N. D., Scarabelli L., Liz-Marzán L. M., Murphy C. J. (2014). Anisotropic Noble Metal Nanocrystal growth: The Role of Halides. Chem. Mater..

[cit44] Wiley B., Herricks T., Sun Y., Xia Y. (2004). Polyol Synthesis of Silver Nanoparticles: Use of Chloride and Oxygen to Promote the Formation of Single-Crystal, Truncated Cubes and Tetrahedrons. Nano Lett..

[cit45] Ha T. H., Koo H.-J., Chung B. H. (2007). Shape-Controlled Syntheses of Gold Nanoprisms and Nanorods Influenced by Specific Adsorption of Halide Ions. J. Phys. Chem. C.

[cit46] Chen Z., Balankura T., Fichthorn K. A., Rioux R. M. (2019). Revisiting the Polyol Synthesis of Silver Nanostructures: Role of Chloride in Nanocube Formation. ACS Nano.

[cit47] Zeng J.-L., Guan Z.-J., Du Y., Nan Z.-A., Lin Y.-M., Wang Q.-M. (2016). Chloride-Promoted Formation of a Bimetallic Nanocluster Au_80_Ag_30_ and the Total Structure Determination. J. Am. Chem. Soc..

[cit48] Xie C., Niu Z., Kim D., Li M., Yang P. (2020). Surface and Interface Control in Nanoparticle Catalysis. Chem. Rev..

[cit49] Jin Y., Zhang C., Dong X.-Y., Zang S.-Q., Mak T. C. W. (2021). Shell engineering to achieve modification and assembly of atomically-precise silver clusters. Chem. Soc. Rev..

[cit50] Whitesides G. M., Grzybowski B. (2002). Self-Assembly at All Scales. Science.

[cit51] Nikoobakht B., El-Sayed M. A. (2003). Preparation and Growth Mechanism of Gold Nanorods (NRs) Using Seed-Mediated Growth Method. Chem. Mater..

[cit52] Polte J., Ahner T. T., Delissen F., Sokolov S., Emmerling F., Thünemann A. F., Kraehnert R. (2010). Mechanism of Gold Nanoparticle Formation in the Classical Citrate Synthesis Method Derived from Coupled In Situ XANES and SAXS Evaluation. J. Am. Chem. Soc..

[cit53] Zeng C., Li T., Das A., Rosi N. L., Jin R. (2013). Chiral Structure of Thiolate-Protected 28-Gold-Atom Nanocluster Determined by X-ray Crystallography. J. Am. Chem. Soc..

[cit54] Kang X., Zhu M. (2019). Transformation of Atomically Precise Nanoclusters by Ligand-Exchange. Chem. Mater..

[cit55] Heaven M. W., Dass A., White P. S., Holt K. M., Murray R. W. (2008). Crystal Structure of the Gold Nanoparticle [N(C_8_H_17_)_4_][Au_25_(SCH_2_CH_2_Ph)_18_]. J. Am. Chem. Soc..

[cit56] Wilcoxon J. P., Provencio P. (2003). Etching and Aging Effects in Nanosize Au Clusters investigated Using High-Resolution Size-Exclusion Chromatography. J. Phys. Chem. B.

[cit57] Yao Q., Yuan X., Fung V., Yu Y., Leong D. T., Jiang D., Xie J. (2017). Understanding seed-mediated growth of gold nanoclusters at molecular level. Nat. Commun..

[cit58] Song Y., Li Y., Zhou M., Li H., Xu T., Zhou C., Ke F., Huo D., Wan Y., Jie J., Xu W. W., Zhu M., Jin R. (2022). Atomic structure of a seed-sized gold nanoprism. Nat. Commun..

[cit59] Nakamoto M., Yamamoto M., Fukusumi M. (2002). Thermolysis of gold(i) thiolate complexes producing novel gold nanoparticles passivated by alkyl groups. Chem. Commun..

[cit60] Mortensen J. J., Larsen A. H., Kuisma M., Ivanov A. V., Taghizadeh A., Peterson A., Haldar A., Dohn A. O., Schäfer C., Jónsson E. Ö., Hermes E. D., Nilsson F. A., Kastlunger G., Levi G., Jónsson H., Häkkinen H., Fojt J., Kangsabanik J., Sødequist J., Lehtomäki J., Heske J., Enkovaara J., Winther K. T., Dulak M., Melander M. M., Ovesen M., Louhivuori M., Walter M., Gjerding M., Lopez-Acevedo O., Erhart P., Warmbier R., Würdemann R., Kaappa S., Latini S., Boland T. M., Bligaard T., Skovhus T., Susi T., Maxson T., Rossi T., Chen X., Schmerwitz Y. L. A., Schiøtz J., Olsen T., Jacobsen K. W., Thygesen K. S. (2024). GPAW: An open Python package for electronic structure calculations. J. Chem. Phys..

[cit61] Perdew J. P., Burke K., Ernzerhof M. (1996). Generalized Gradient Approximation Made Simple. Phys. Rev. Lett..

[cit62] Negishi Y., Nakazaki T., Malola S., Takano S., Niihori Y., Kurashige W., Yamazoe S., Tsukuda T., Häkkinen H. (2015). A Critical Size for Emergence of Nonbulk Electronic and Geometric Structures in Dodecanethiolate-Protected Au Clusters. J. Am. Chem. Soc..

[cit63] Malola S., Kaappa S., Häkkinen H. (2019). Role of Nanocrystal Symmetry in the Crossover Region from Molecular to Metallic Gold Nanoparticles. J. Phys. Chem. C.

[cit64] Malola S., Lehtovaara L., Enkovaara J., Häkkinen H. (2013). Birth of the Localized Surface Plasmon Resonance in Monolayer-Protected Gold Nanoclusters. ACS Nano.

[cit65] (a) CCDC 2562216: Experimental Crystal Structure Determination, 2026, 10.25505/fiz.icsd.cc2s0645

