# Supplementary material for: A series of structurally related hcp-like silver clusters suggests faceted lateral cluster growth
Source: Chem Sci. 2026 Aug 6;17(39):18802–16. doi: 10.1039/d6sc06118d (PMC13488696; doi:10.1039/d6sc06118d)
Supplement: Supplementary file 1 [file SC-017-D6SC06118D-s001.pdf]

Supplementary Information (SI) for Chemical Science.
This journal is © The Royal Society of Chemistry 2026

Supporting Information

A Series of Structurally Related hcp-Like Silver Clusters Suggests Faceted Lateral Cluster Growth

Mike Alexander Kordan[a], Claudio Schrenk[a], Sami Malola[b], Hannu Häkkinen[b,c], Andreas Schnepf[a*]

[a]Institut für Anorganische Chemie, Universität Tübingen, Auf der Morgenstelle 18, 72076 Tübingen, Germany [b]Department of Physics, Nanoscience Center, University of Jyväskylä, 40014 Jyväskylä, Finland [c]Department of Chemistry, Nanoscience Center, University of Jyväskylä, 40014 Jyväskylä, Finland

Table of Contents

1. Experimental 3

2. Crystal Data and Structure Determination of **Ag_{128}**, **Ag_{132}**, **Ag_{146}** and **Ag_{160}** 4

 2.1. Crystallography 4

 2.2. Comparison of Selected Bond Lengths, Angles and Cluster Dimensions 11

 2.3. Continuous Shape Measure Calculations 13

 2.4. Out-of-Plane Undulation Calculations 14

 2.5. Space Filling Models of **Ag_{128}**, **Ag_{132}** and **Ag_{146}** 18

 2.6. Comparison Between the Title Compounds and hcp Lattice 19

 2.7. Further Comparison Between the Title Compounds 20

3. SEM and EDX Measurements 21

 3.1. Ag_{128} 21

 3.2. Other Compounds 25

4. Quantum Chemical Calculations 27

5. On the Colors Used 29

6. References 30

1. Experimental

General procedures. All experiments were conducted under an argon atmosphere using standard Schlenk techniques in a dark room illuminated by a red safelight with a wavelength of 630 nm. During transfer to analytical instruments, samples were cooled by dry ice and shielded from light with aluminum foil. Both rigorous light exclusion and careful temperature control are essential for successful synthesis and analysis.

Chemicals. L-Selectride ($Li[HB(sec\text{-}Bu)_3]$) (1 M in THF) was purchased from Sigma-Aldrich and was used as received. Toluene was purchased from Thermo Scientific and was dried over Na/benzophenone prior to use. THF was purchased from Sigma-Aldrich and was dried over Na/benzophenone twice prior to use. Pr_3PAgCl was prepared according to a modified literature procedure.[1]

Synthesis of $Ag_{128}(PPr_3)_{28}Cl_6$ (Ag_{128}), $Ag_{132}(PPr_3)_{28}Cl_8$ (Ag_{132}), $Ag_{146}(PPr_3)_{30}Cl_6$ (Ag_{146}), and $Ag_{160}(PPr_3)_xCl_y$ (Ag_{160}).

The synthesis procedures for all compounds are quite similar. Therefore, we give an exemplary protocol for **Ag_{128}**, whereby the parameters that led to isolable products are underlined.

3.0 ml of a Pr_3PAgCl solution (0.91 g, 3.0 mmol, 1 M in THF; <u>1.0 mmol to 5.0 mmol</u>) were added in 30 mL toluene (<u>30 mL to 80 mL, THF or toluene or 1:1 THF:toluene</u>) and cooled to –78°C. 2.1 mL of a 1 M solution of $Li[HB(sec\text{-}Bu)_3]$ in THF (2.1 mmol, 0.7 eq.; <u>0.6 eq. to 0.8 eq.</u>) was added, giving a pale-yellow solution. The mixture was allowed to warm to –25°C (<u>–5°C to –30°C</u>) over 2 h (<u>1 h to 5 h</u>). After ripening for 24 h (<u>24 h to 72 h</u>) in a –30°C freezer, the brownish-red solution was filtered from black precipitate and stored at –30°C for 2 months (<u>1 month to 6 months</u>) to give dark red crystals of **Ag_{128}** (150 mg, 35 % yield based on Ag; <u>20-35%</u>). No product mixtures were observed.

The reactions leading to the compounds $Ag_{128}(PPr_3)_{28}Cl_6$ (**Ag_{128}**), $Ag_{132}(PPr_3)_{28}Cl_8$ (**Ag_{132}**), $Ag_{146}(PPr_3)_{30}Cl_6$ (**Ag_{146}**) and $Ag_{160}(PPr_3)_xCl_y$ (**Ag_{160}**) involve the reduction of the precursor Pr3PAgCl. Even though we kept the reaction conditions as similar as possible for routine laboratory experiments (e.g. 150 min reaction time, –25°C end temperature, 0.7 equivalents of L-Selectride), we could not predict the resulting cluster *a priori*. In the hope of identifying any factors that determine the final product, we conducted further reactions with substantially different conditions. However, several factors such as the absolute amount of substance, relative stoichiometry, concentration, end temperature, time to warm up and time to crystallization did not indicate a clear trend. This was further complicated by the fact that over half of the batches yielded crystals not suitable for SCXRD. Recrystallization proved to be

unfruitful. Only the choice of solvent seemed to have a reproducible influence on the reaction outcome: **Ag$_{128}$** regularly formed both in toluene and THF. On the other hand, **Ag$_{132}$** was mainly detected in reactions conducted in THF, while **Ag$_{146}$** exhibited a strong preference towards toluene. Yet even these conditions are not absolute but barely observed trends. In total, dozens of batches with a variety of conditions were examined. Table S1 lists selected reaction conditions and shows that no clear trend can be established.

Table S1. Selected examples of reaction conditions for the synthesis of the metalloid clusters **Ag$_{128}$**, **Ag$_{132}$**, **Ag$_{146}$** and **Ag$_{160}$**.

Reaction №	Solvent	c(Pr_3PAgCl) [mmol/l]	equivalents L-Selectride	T [°C]	Reaction time [min]	Crystallization time	Product
1	THF	50	0.7	−20°C	270	3 months	**Ag$_{128}$**
2	toluene	100	0.7	−20°C	90	4 months	**Ag$_{128}$**
3	toluene	120	0.7	−25°C	150	2 months	**Ag$_{128}$**
4	THF	100	0.6	−25°C	240	2.5 months	**Ag$_{132}$**
5	toluene	25	0.6	−20°C	240	4 months	**Ag$_{132}$**
6	THF	50	0.8	−25°C	200	5 months	**Ag$_{146}$**
7	toluene	250	0.6	−30°C	240	1.5 months	**Ag$_{146}$**
8	THF	100	0.7	−15°C	300	1 month	**Ag$_{160}$**

2. Crystal Data and Structure Determination of **Ag$_{128}$**, **Ag$_{132}$**, **Ag$_{146}$** and **Ag$_{160}$**

2.1. Crystallography

All samples showed a sensitivity to temperature and light, so even the measurement setup was challenging. To select a suitable crystal, a number of crystals from the reaction batch were put in a precooled perfluorinated oil drop and were checked under a dimmed light microscope as fast as possible. Suitable candidates were mounted on the diffractometer at 100 K and 150 K. The data were collected on a Rigaku XtaLAB Synergy-S X-ray diffractometer for single crystal X-ray diffraction equipped with PhotonJet-S source for monochromated MoKα radiation (λ = 0.71073 Å) and an Oxford Cryosystems cryostat. A numerical absorption correction based on Gaussian integration over a multifaceted crystal model was applied using the SCALE3 ABSPACK scaling algorithm. The structure was solved by direct methods and refined against F^2 for all observed reflections. Programs used: SHELXT and SHELXL[2] within the Olex2 program package.[3] To get a reliable refinement, for some of the organic propyl groups the routine FragmentDB[4] was used. Also, the SQUEEZE routine implemented in the PLATON program package[5] was used to identify all solvent-containing voids. The H atom positions in all compounds were refined using a riding model.

Ag_{128}: 1 void containing 1957 Å³ and 622 electrons, fitting to 12 toluene per unit cell.

Special issues concerning **Ag_{132}:**

The structure model presented here is the best model obtained from numerous measurements using small to large and thin to thick deep red crystal blocks. Since the smaller ones did not diffract well below a resolution of 1 Å, we first tried to use CuKα instead of MoKα, which is the more powerful X-ray source in our setup. However, we observed degradation of the crystals within one hour. Using bigger and thicker crystals with MoKα radiation, we found better diffraction, but still not well below 0.87 Å. Several factors may account for this:

- high amount of disordered organic groups in the phosphine
- crystal damage during preparation since they are hard to handle due to high sensitivity to temperature and light. Especially light cannot be excluded due to the necessity of a light microscope to select a good candidate.
- crystal damage during the measurements due to light, which cannot be excluded completely, especially warning lamps, etc.

Given these limitations, the presented measurement was the best measurement by far and could be solved and refined to acceptable values (see Table S3).

Special issues concerning **Ag_{146}:**

The structure model presented here is the best model obtained from numerous measurements using small to big and thin to thick deep red crystal blocks. In this case, also MoKα radiation was used for the best measurement, yet even here degradation processes were observed. Due to those problems, the presented structure model of **Ag_{146}** and especially the organic phosphines could only be refined as a fully restrained shell around the metal core. Due to these constraints, we were able to refine all phosphines, but H atoms could not be added reliably. SQUEEZE was therefore not applied. Accordingly, only the atom connectivity of the cluster core (Ag/P/Cl) is considered reliable. Table S4 contains the summary of these rudimentary data.

Special issues concerning **Ag_{160}:**

The structure model is limited to connectivity of Ag/P/Cl. Even if in some cases the organic shell might be identified during refinement, we were not able to determine reliable positions of the C atoms, which is also due to the poor quality of the measurement. Table S5 contains the summary of these rudimentary data.

Table S2. Supplementary crystallographic information for **Ag_{128}**.

	$Ag_{128}(PPr_3)_{28}Cl_6$ (**Ag_{128}**)
Empirical formula	$C_{252}H_{588}Ag_{128}Cl_6P_{28}$
Formula weight	18506.41
T [K]	149.99(10)
Crystal system	triclinic
Space group	P-1
a [Å]	20.28971(10)
b [Å]	25.69425(13)
c [Å]	27.13270(13)
α [°]	105.0240(4)
β [°]	107.3647(4)
γ [°]	96.8274(4)
V [Å³]	12739.99(12)
Z	1
ρ_{calc} [g/cm³]	2.412
μ [mm^{-1}]	4.930
F(000)	8638.0
Crystal size [mm³]	0.349 × 0.249 × 0.15
Radiation	Mo Kα (λ = 0.71073)
2θ	3.928 to 52.746
Index range	–25 ≤ h ≤ 25, –32 ≤ k ≤ 32, –33 ≤ l ≤ 33
Reflections collected	547789
Independent reflections	52093 [R_{int} = 0.0436, R_{sigma} = 0.0183]
Data/restraints/parameters	52093/420/1817
GooF	1.092
R [I ≥ 2σ(I)]	R_1 = 0.0402, wR_2 = 0.1006
R [all data]	R_1 = 0.0504, wR_2 = 0.1096
Largest peak/hole [e/Å³]	2.01/–1.38
CCDC	2562218

Table S3. Supplementary crystallographic information for **Ag_{132}**.

	$Ag_{132}(PPr_3)_{28}Cl_8$ (**Ag_{132}**)
Empirical formula	$C_{252}H_{588}Ag_{132}Cl_8P_{28}$
Formula weight	19008.79
T [K]	100.00(12)
Crystal system	triclinic
Space group	P-1
a [Å]	25.7964(2)
b [Å]	33.1195(4)
c [Å]	33.6636(3)
α [°]	97.9180(10)
β [°]	105.5020(10)
γ [°]	105.1130(10)
V [Å³]	26082.4(5)
Z	2
ρ_{calc} [g/cm³]	2.420
μ [mm^{-1}]	4.973
F(000)	17720.0
Crystal size [mm³]	0.185 × 0.08 × 0.05
Radiation	Mo Kα (λ = 0.71073)
2θ	3.864 to 50.054
Index range	–30 ≤ h ≤ 30, –39 ≤ k ≤ 39, –40 ≤ l ≤ 38
Reflections collected	403050
Independent reflections	92075 [R_{int} = 0.0679, R_{sigma} = 0.0616]
Data/restraints/parameters	92075/2881/3703
GooF	1.114
R [I ≥ 2σ(I)]	R_1 = 0.1104, wR_2 = 0.3094
R [all data]	R_1 = 0.1642, wR_2 = 0.3523
Largest peak/hole [e/Å³]	4.46/–2.43
CCDC	2562217

Table S4. Supplementary crystallographic information for **Ag_{146}**.

	$Ag_{146}(PPr_3)_{30}Cl_6$ (**Ag_{146}**)
Empirical formula	$Ag_{146}C_{270}Cl_6P_{30}$
T [K]	150
Crystal system	triclinic
Space group	P-1
a [Å]	20.46160(10)
b [Å]	28.5903(2)
c [Å]	48.7577(3)
α [°]	85.0090(10)
β [°]	83.7870(10)
γ [°]	69.7320(10)
V [Å³]	26562.7(3)
Z	2
Crystal size [mm³]	0.255 x 0.174 x 0.061
Radiation	Mo Kα (λ = 0.71073)
2θ	3.864 to 50.054
Index range	–24 ≤ h ≤ 24, –34 ≤ k ≤ 34, –58 ≤ l ≤ 58
Reflections collected	517610
Independent reflections	93688 [R_{int} = 0.0702, R_{sigma} = 0.0480]
CCDC	2562219

Table S5. Supplementary crystallographic information for **Ag_{160}**.

	$Ag_{160}(PPr_3)_{32}Cl_6$ (**Ag_{160}**)[1]
Empirical formula	$Ag_{160}Cl_6P_{32}$
T [K]	150
Crystal system	triclinic
Space group	P-1
a [Å]	19.9640(16)
b [Å]	28.637(2)
c [Å]	29.179(2)
α [°]	114.018(2)
β [°]	109.768(2)
γ [°]	91.053(3)
V [Å³]	14101(2)
Z	1
Crystal size [mm³]	0.256 × 0.088 × 0.045
Radiation	Mo Kα (λ = 0.71073)
2θ	1.584 to 49.5
Index range	–23 ≤ h ≤ 23, –33 ≤ k ≤ 33, –34 ≤ l ≤ 34
Reflections collected	247159
Independent reflections	48283 [R_{int} = 0.1224, R_{sigma} = 0.1013]
CCDC	2562216

[1]A tentative connectivity model containing 32 P and 6 Cl sites was used for crystallographic modelling; because of the limited diffraction data, this ligand composition is not established experimentally.

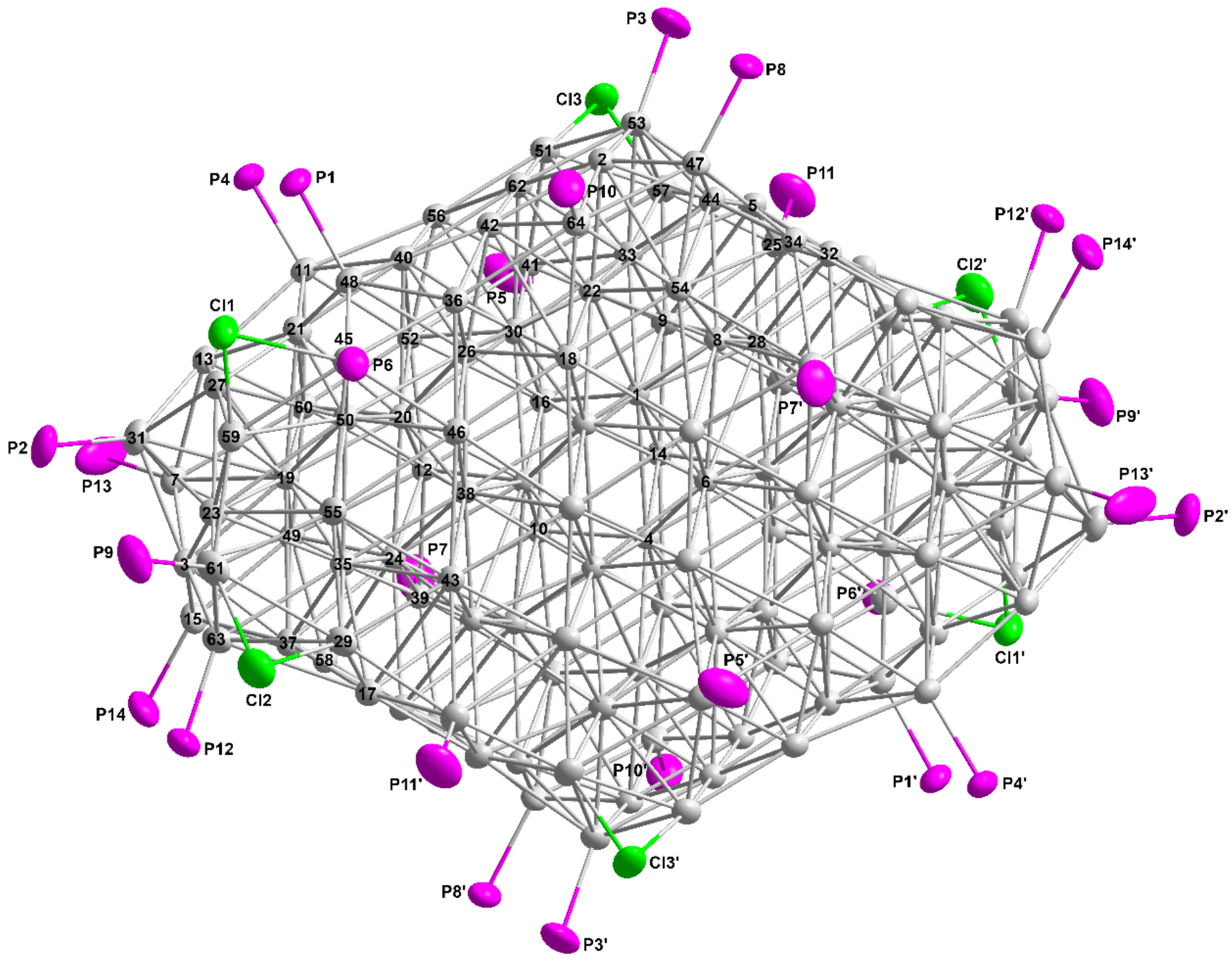

Figure S1. Atom names of **Ag_{128}** excluding C and H. Ag (grey), P (pink), Cl (green); thermal ellipsoids are set at 50 % probability. For Ag, only atom names for the asymmetric unit are given.

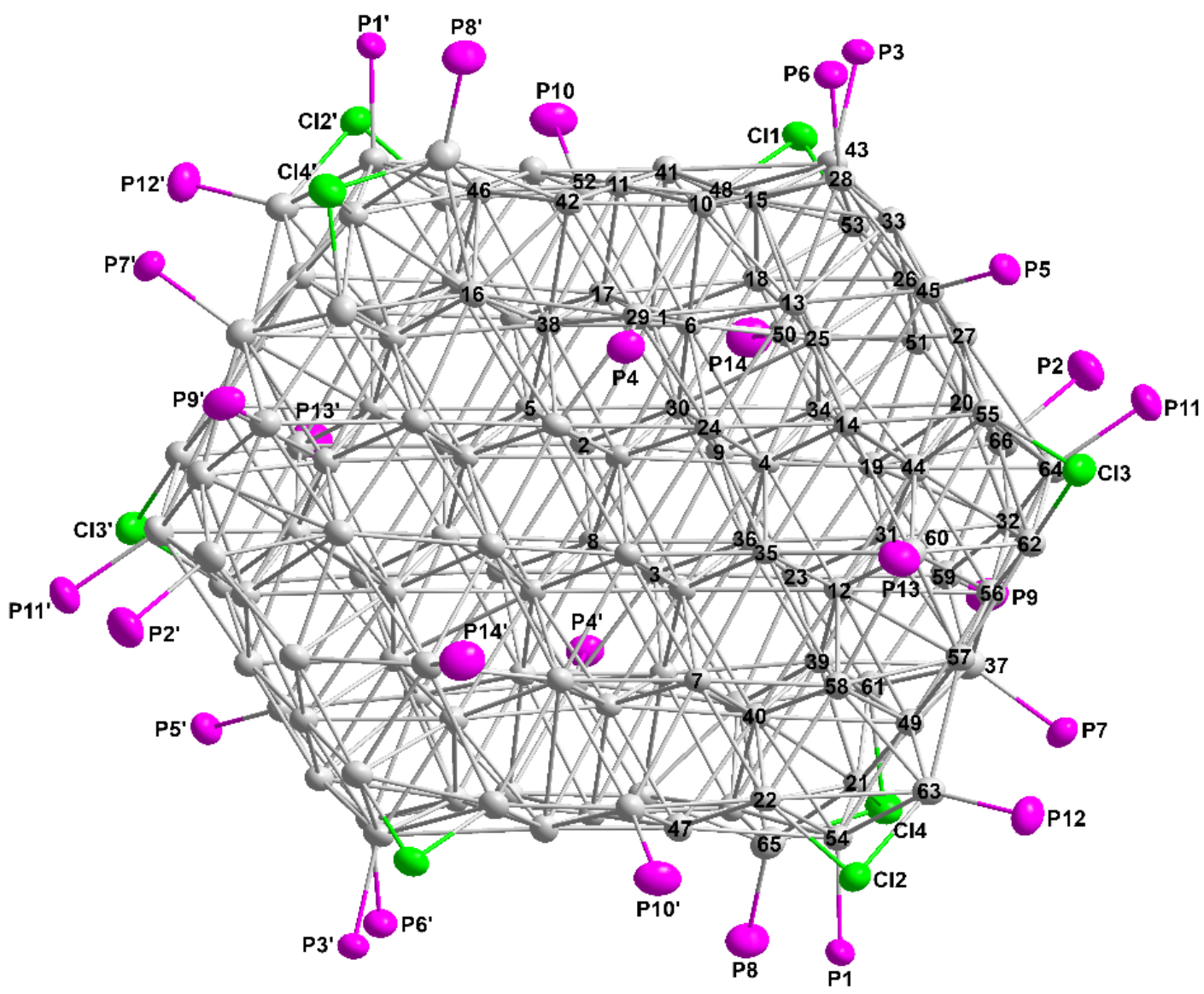

Figure S2. Atom names of **Ag_{132}** excluding C and H. Ag (grey), P (pink), Cl (green); thermal ellipsoids are set at 50 % probability. For Ag, only atom names for the asymmetric unit are given.

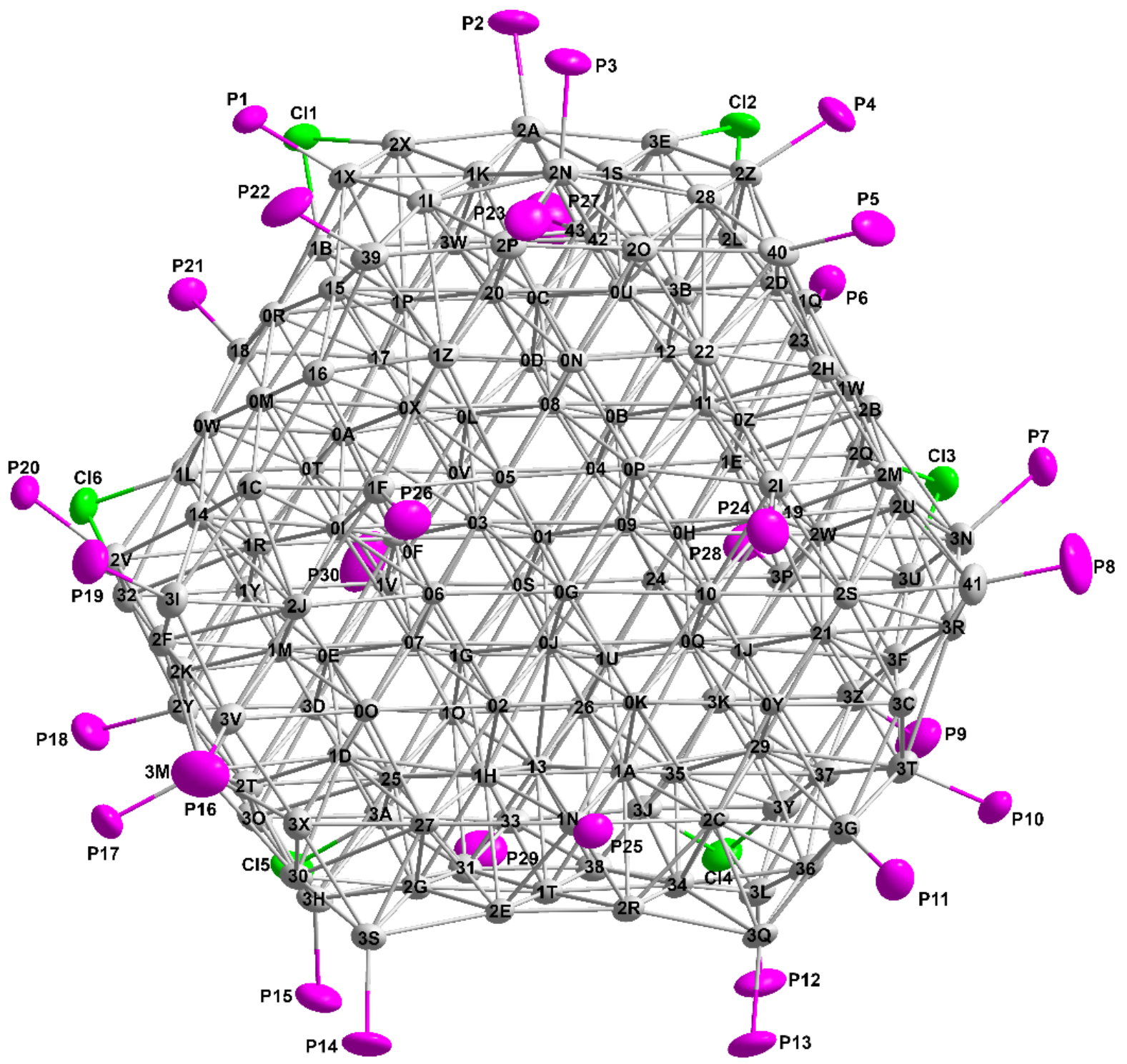

Figure S3. Atom names of $\mathbf{Ag_{146}}$ excluding C and H. Ag (grey), P (pink), Cl (green); thermal ellipsoids are set at 50 % probability.

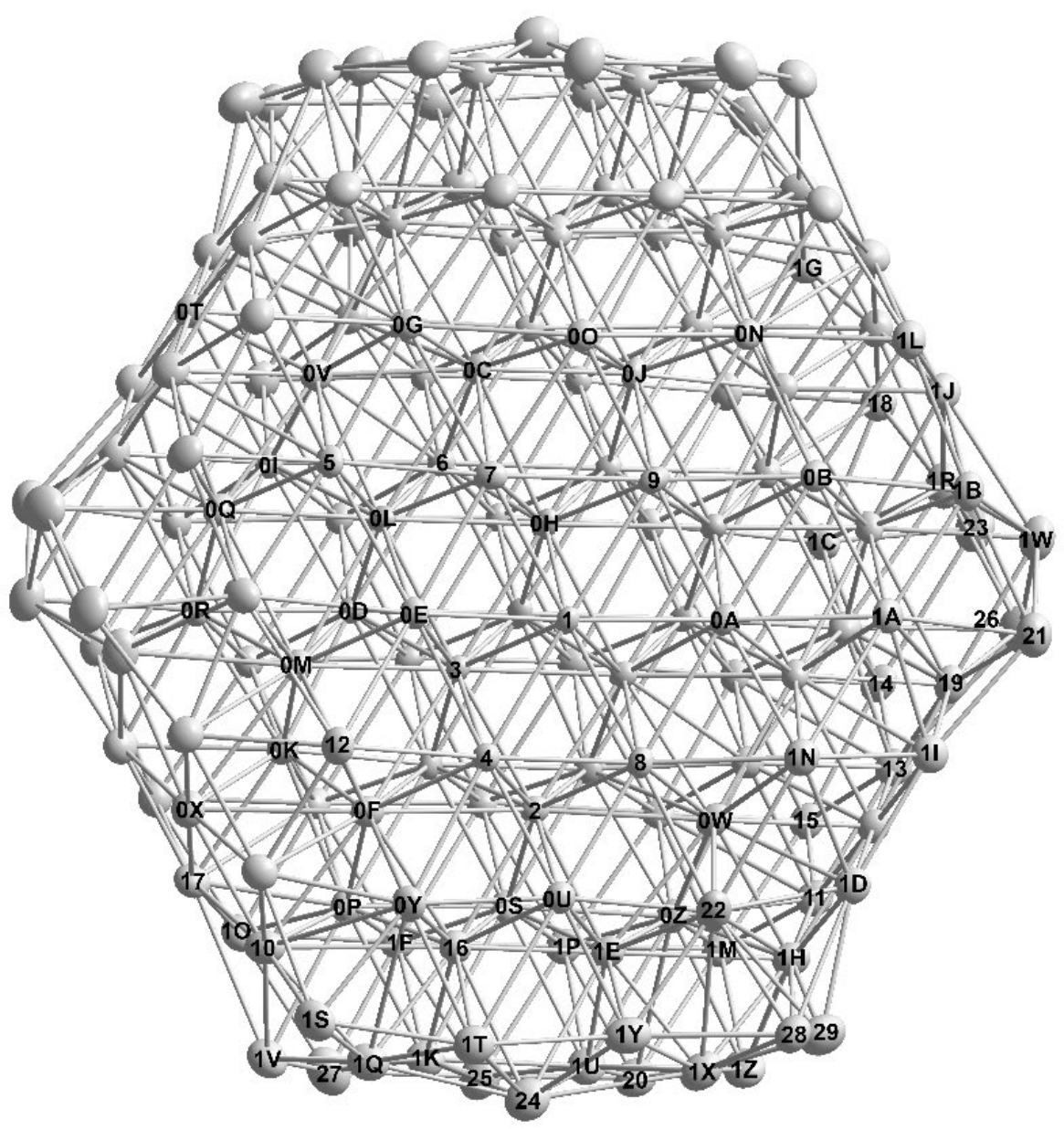

Figure S4. Atom names of $\mathbf{Ag_{160}}$ excluding C, H, P and Cl. Ag (grey); thermal ellipsoids are set at 50 % probability. Only atom names for the asymmetric unit are given.

2.2. Comparison of Selected Bond Lengths, Angles and Cluster Dimensions

Table S6. Dimensions of the silver cores of **Ag_{108}**, **Ag_{128}**, **Ag_{132}**, **Ag_{146}** and **Ag_{160}**.

	Ag_{108}	Ag_{128}	Ag_{132}	Ag_{146}[1]	Ag_{160}[1]
Length [nm]	1.3	1.9	1.9	1.9	1.9
Width [nm]	0.8	1.6	1.3	1.6	1.9
Height [pm]	776	767	763	768	764
Side A [nm]	0.78	0.58	0.97	0.92	0.95
Side B [nm]	0.77	0.93	0.78	0.94	1.10
Side C [nm]	0.80	1.12	0.92	0.93	0.93
Side D [nm]	[2]	[2]	[2]	0.95	[2]
Side E [nm]	[2]	[2]	[2]	0.92	[2]
Side F [nm]	[2]	[2]	[2]	0.95	[2]
∠(A/B)	119.4°	116.8°	123.8°	120.2°	120.1°
∠(B/C)	119.2°	123.3°	116.7°	118.7°	119.3°
∠(C/A) / ∠(C/D)	121.2°	119.4°	118.9°	120.3°	118.6°
∠(D/E)	[2]	[2]	[2]	120.2°	[2]
∠(E/F)	[2]	[2]	[2]	119.0°	[2]
∠(F/A)	[2]	[2]	[2]	119.4°	[2]
Angle sum[3]	719.6°	719.0°	718.8°	717.8°	716.0°

[1]Preliminary results based on incomplete data; for comparison only.

[2]Due to symmetry, sides A and D, B and E, and C and F are equivalent respectively.

[3]For a polygon, the sum of interior angles equals to (n−2) x 180°. For hexagons, this equals to 720°.

Table S7. Average Ag—Ag bond lengths for **Ag_{108}**, **Ag_{128}**, **Ag_{132}** and **Ag_{146}** in pm.

		Ag_{108}	Ag_{128}	Ag_{132}	Ag_{146}[1]
Intralayer	1st	304.5	306.5	304.1	307.1
	2nd	304.2	307.3	313.2	316.2
	3rd	[2]	[2]	[2]	314.1
	4th	[2]	[2]	[2]	305.7
	Average	304.4	306.8	307.0	310.5
Interlayer	1st to 2nd	302.2	305.1	305.9	305.6
	2nd to 3rd	308.2	312.3	306.4	306.2
	3rd to 4th	[2]	[2]	[2]	308.8
	Average	305.0	308.4	306.1	306.8
Total	Average	304.7	307.7	307.0	308.8

[1]Preliminary results based on incomplete data; for comparison only.

[2]Due to symmetry, the 1st and 4th, and 2nd and 3rd layers are equivalent respectively.

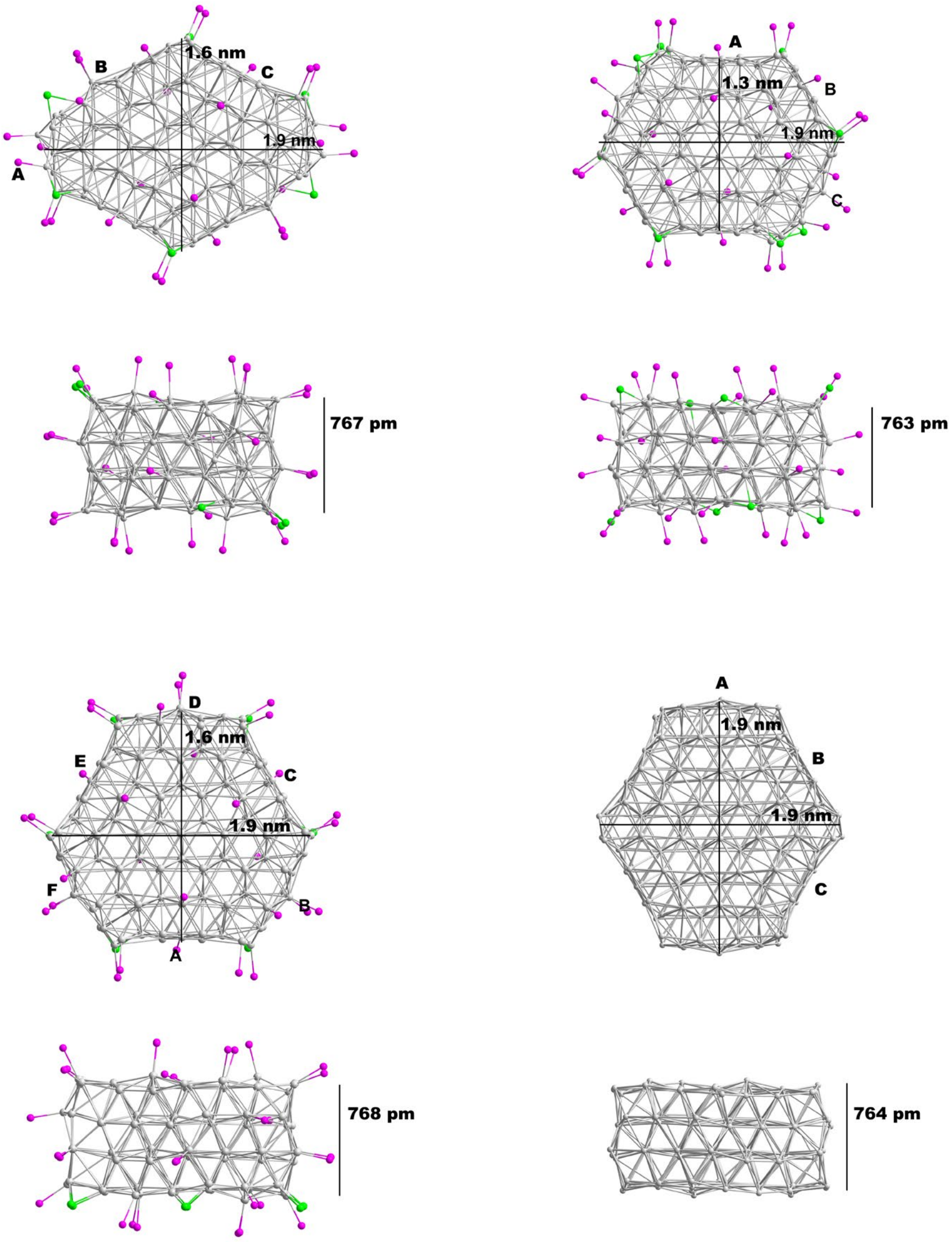

Figure S5. Core dimensions for the metal kernels of $\mathbf{Ag_{128}}$ (top left), $\mathbf{Ag_{132}}$ (top right), $\mathbf{Ag_{146}}$ (bottom left) and $\mathbf{Ag_{160}}$ (bottom right). Sides are named according to Table S6.

Table S8. Average Ag—P/Cl bond lengths, Ag-Cl-Ag angles and dihedral angles between Ag-Cl-Ag moieties and basal facets for $\mathbf{Ag_{108}}$, $\mathbf{Ag_{128}}$, $\mathbf{Ag_{132}}$ and $\mathbf{Ag_{146}}$.

	$\mathbf{Ag_{108}}$	$\mathbf{Ag_{128}}$[1]	$\mathbf{Ag_{132}}$[2]	$\mathbf{Ag_{146}}$
d(Ag—P) [pm]	245.9	246.8	247.7	249.3
d(Ag—Cl) short bonds [pm]	254.8	252.6	257.3 (254.9)	256.0
d(Ag—Cl) long bonds [pm]	266.3	273.2	269.6 (265.9)	260.6
d(Ag—Cl) all bonds [pm]	260.6	262.9	264.1 (260.4)	258.3
Ag-Cl-Ag angle	70.3°	74.0°	81.5° (76.5°)	73.0°
Dihedral angle (Ag-Cl-Ag, outer facet)	47.7°	45.1° (30.1°)	34.2° (40.9°)	44.2°

[1]Values in parentheses include the Ag-Cl-Ag moiety with a dihedral angle near 0°.

[2]Values in parentheses exclude the μ^3-bridging Cl atom. For the μ^3-bridging Cl atom, only one angle is used for calculations.

2.3. Continuous Shape Measure Calculations

The hcp lattice consists of octahedra sharing faces along the c-axis. In the regular, non-distorted case, one can assume them to be of O_h symmetry, i.e. regular octahedra. Therefore, we argue that the degree of deformation for the Ag_6 octahedra found within the metal cores of the title compounds correlates with the overall deviation from hcp packing. In order to quantify this, we performed continuous shape measure (CSM) calculations, derived from the continuous symmetry measures formalism,[6] for every octahedron present within the metal kernel. In this approach, the minimum normalized distance between the observed coordinates and an idealized reference geometry are calculated using the SHAPE 2.1 program.[7]

A CSM value of S = 0 corresponds to a perfect ideal octahedron, whereby larger values of S indicate increasing deviations from the reference structure. A general trend can be inferred from the data: with increasing cluster size, the distortion of the octahedra as well as the spread of calculated distortions diminish. While the latter is true for all compounds, average distortion decreases non-monotonic and rises sharply from **Ag_{132}** to **Ag_{146}**. It is possible that the reason for this lies within the decreasing quality of crystal structure data for **Ag_{146}** and **Ag_{160}**. Another explanation might be that at these size scale, reconstruction's influence on octahedra regularity is more important than cluster size. We interpret this as follows: We expect metalloid clusters to have more "metal-like" structural features the larger they grow, i.e. in this case to consist of less distorted octahedra. As the influence of surface reconstruction becomes less pronounced the larger the cluster is, both extreme outliers and therefore the spread of deviations decrease.

Table S9. Results of CSM calculations for **Ag_{108}**, **Ag_{128}**, **Ag_{132}**, **Ag_{146}** and **Ag_{160}**.

	**Ag_{108}**	**Ag_{128}**	**Ag_{132}**	**Ag_{146}**[1]	**Ag_{160}**[1]
Min S(octahedron)	3.0	3.6	3.8	9.0	6.5
Max S(octahedron)	23.3	16.2	18.2	25.8	15.4
Median S(octahedron)	7.5	6.9	6.9	14.0	9.0
Mean S(octahedron)	8.7	8.0	7.7	14.7	9.3
MAD S(octahedron)	3.1	2.5	2.3	2.3	1.6
Mean S(trigonal prism)	16.8	17.6	16.9	21.6	19.2
Mean S(pentagonal bipyramidal)	24.1	24.5	24.7	26.2	25.1
Mean S(Johnson solid J2)	25.2	25.5	25.9	25.9	25.9

[1]Preliminary results based on incomplete data; for comparison only.

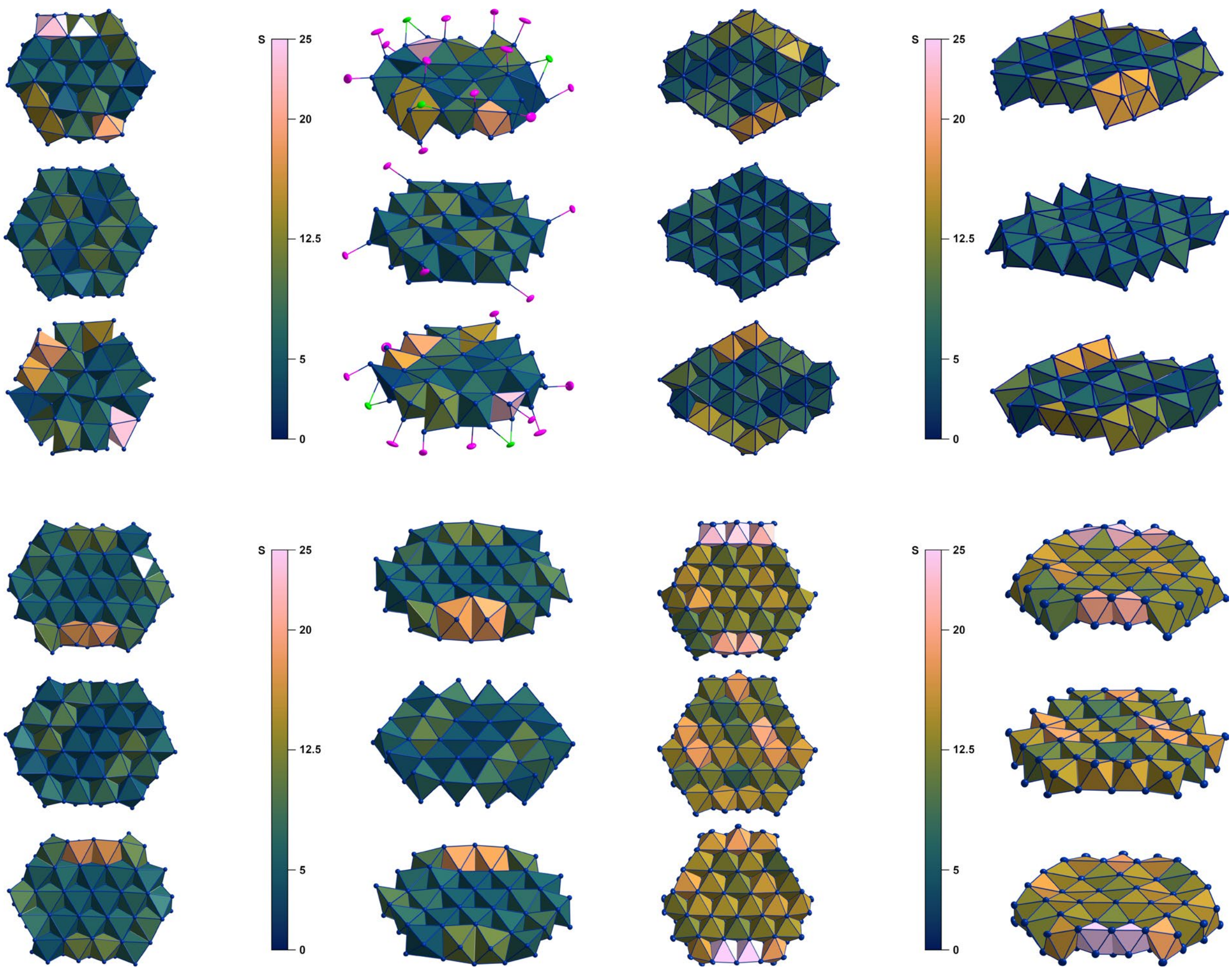

Figure S6. Continuous shape measures S for **Ag_{108}** (top left), **Ag_{128}** (top right), **Ag_{132}** (bottom left) and **Ag_{146}** (bottom right), each split into three layers of octahedra. For the first three entries, only a few strongly distorted octahedra are present, while the latter features an overall more prominent distortion. In comparison to the outer ones, significantly lower S values can be found for the middle octahedra sheets.

2.4. Out-of-Plane Undulation Calculations

For out-of-plane undulation calculations, we adopted a simple procedure: First, within the Diamond 4 program[8] we created a least-squares plane through all 27/32/33/36 respective 37 Ag atoms (**Ag_{108}**/**Ag_{128}**/**Ag_{132}**/**Ag_{146}**) that make up each individual layer. Next, we measured the perpendicular distance of each atom from its layer's least-square plane. The out-of-plane undulation was then quantified using root-mean-square (RMS) deviation, mean absolute deviation (MAD) and peak-to-peak values. Here, RMS is interpreted as the overall roughness/undulation, while the ratio MAD/RMS and peak-to-peak values give insight into whether the undulation is spread uniformly or is dominated by a few extreme outliers, with a MAD/RMS ratio of 1 equating uniformity.

As can be seen from Table S10, the overall undulation decreases from **Ag_{128}** to **Ag_{146}**, consistent with the expectation that larger molecules are structurally more "metal-like" and therefore less corrugated, as reflected by decreasing RMS and MAD values. Interestingly, the comparison between inner and outer layers is less straightforward: the RMS values suggest that both inner and outer Ag layers are undulated to a similar

extent. Yet, for all clusters considered, the outer layers exhibit around 20-50 % higher peak-to-peak distances compared to the inner ones. Additionally, the MAD/RMS ratios are consistently smaller for the outer silver layers. This indicates that for the outer layers, the undulation is dominated by a few strongly displaced Ag atoms, i.e. mainly phosphine-bearing atoms. The Ag atoms of the inner layers instead show a more homogeneous distribution of atomic displacements.

Table S10. Values for the out-of-plane undulations of the Ag atoms within the cluster compounds $\mathbf{Ag_{108}}$, $\mathbf{Ag_{128}}$, $\mathbf{Ag_{132}}$ and $\mathbf{Ag_{146}}$. All values except MAD/RMS ratios are given in pm.

		$\mathbf{Ag_{108}}$	$\mathbf{Ag_{128}}$	$\mathbf{Ag_{132}}$	$\mathbf{Ag_{146}}$
Highest deviation[1]	Outer Layers	–80.5 / 40.8	–88.3 / 75.1	–74.6 / 48.7	–77.9 / 71.9
	Inner Layers	–44.9 / 39.9	–52.0 / 87.7	–44.4 / 38.7	–52.5 / 49.6
	Total	–80.5 / 40.8	–88.3 / 87.7	–74.6 / 48.7	–77.9 / 71.9
RMS	Outer Layers	34.5	35.1	33.0	30.7
	Inner Layers	31.8	36.9	31.6	31.5
	Total	33.2	36.0	32.3	31.1
MAD	Outer Layers	26.1	26.3	26.1	23.3
	Inner Layers	30.6	33.9	29.7	29.2
	Total	28.4	30.1	27.9	26.2
MAD/RMS ratio	Outer Layers	0.76	0.75	0.79	0.76
	Inner Layers	0.96	0.92	0.94	0.93
	Total	0.85	0.83	0.86	0.84
Peak-to-peak	Outer Layers	121.3	163.4	123.3	124.1
	Inner Layers	84.8	139.7	83.1	93.6

[1]A positive sign denotes out-of-plane undulation towards the molecule’s center and vice versa.

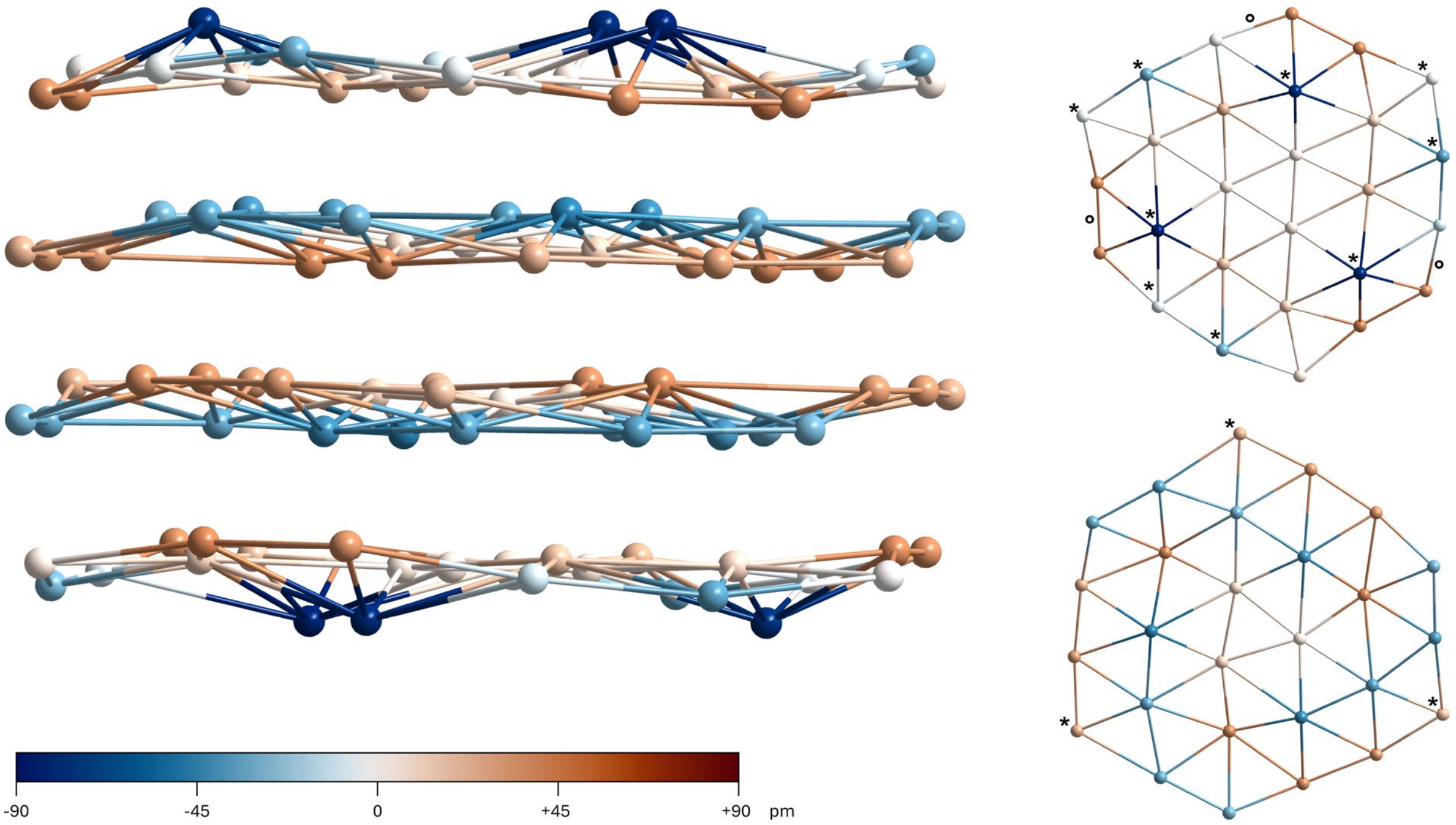

Figure S7. Atomic out-of-plane displacements found within the metal kernel of **Ag_{108}**. Left: side view of the cluster. Right: Top view of the outer (top) and inner (bottom) layers. *Silver atoms bound to P. °Bond between silver atoms bound to μ^2-Cl.

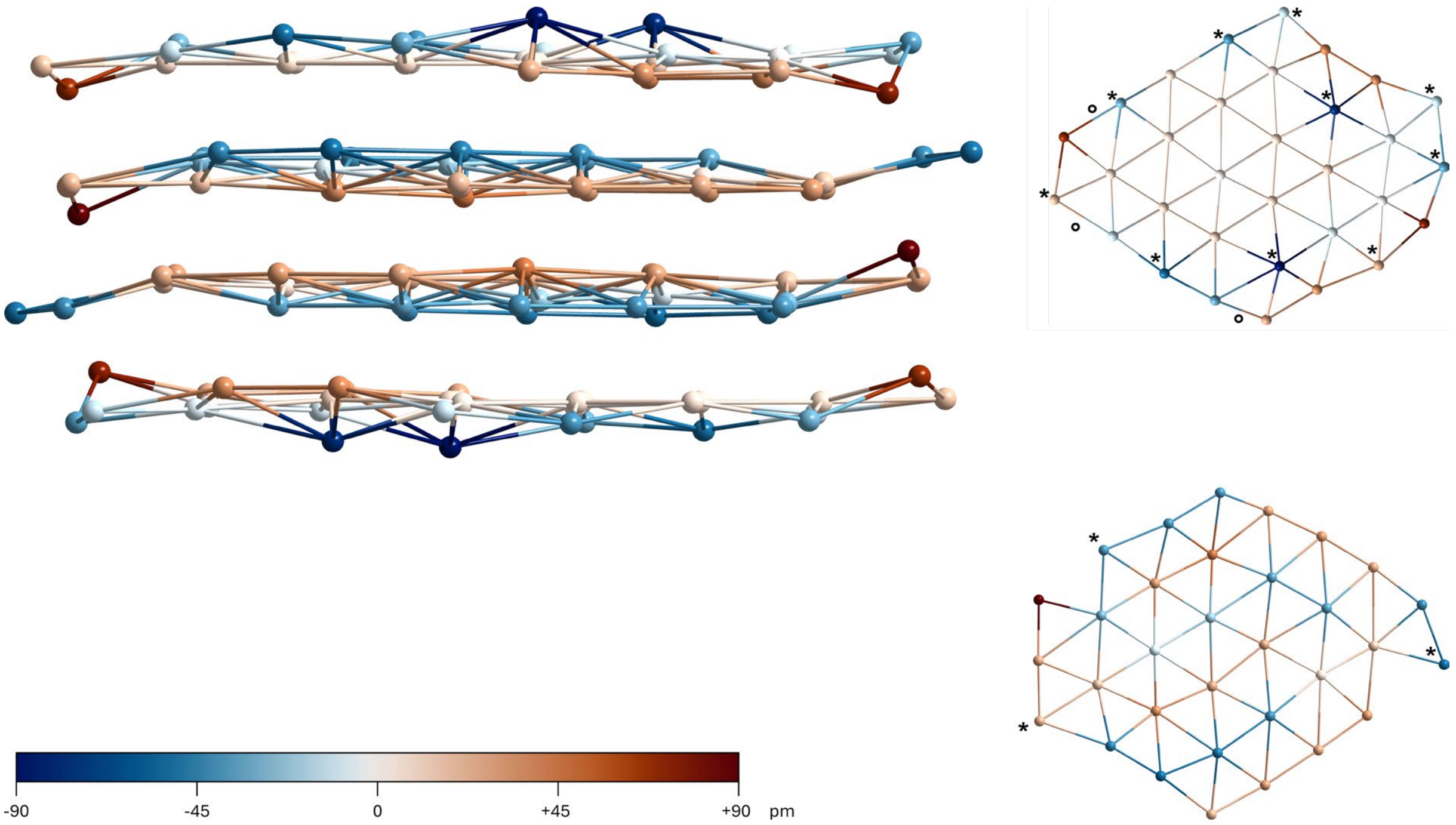

Figure S8. Atomic out-of-plane displacements found within the metal kernel of **Ag_{128}**. Left: side view of the cluster. Right: Top view of the outer (top) and inner (bottom) layers. *Silver atoms bound to P. °Bond between silver atoms bound to μ^2-Cl.

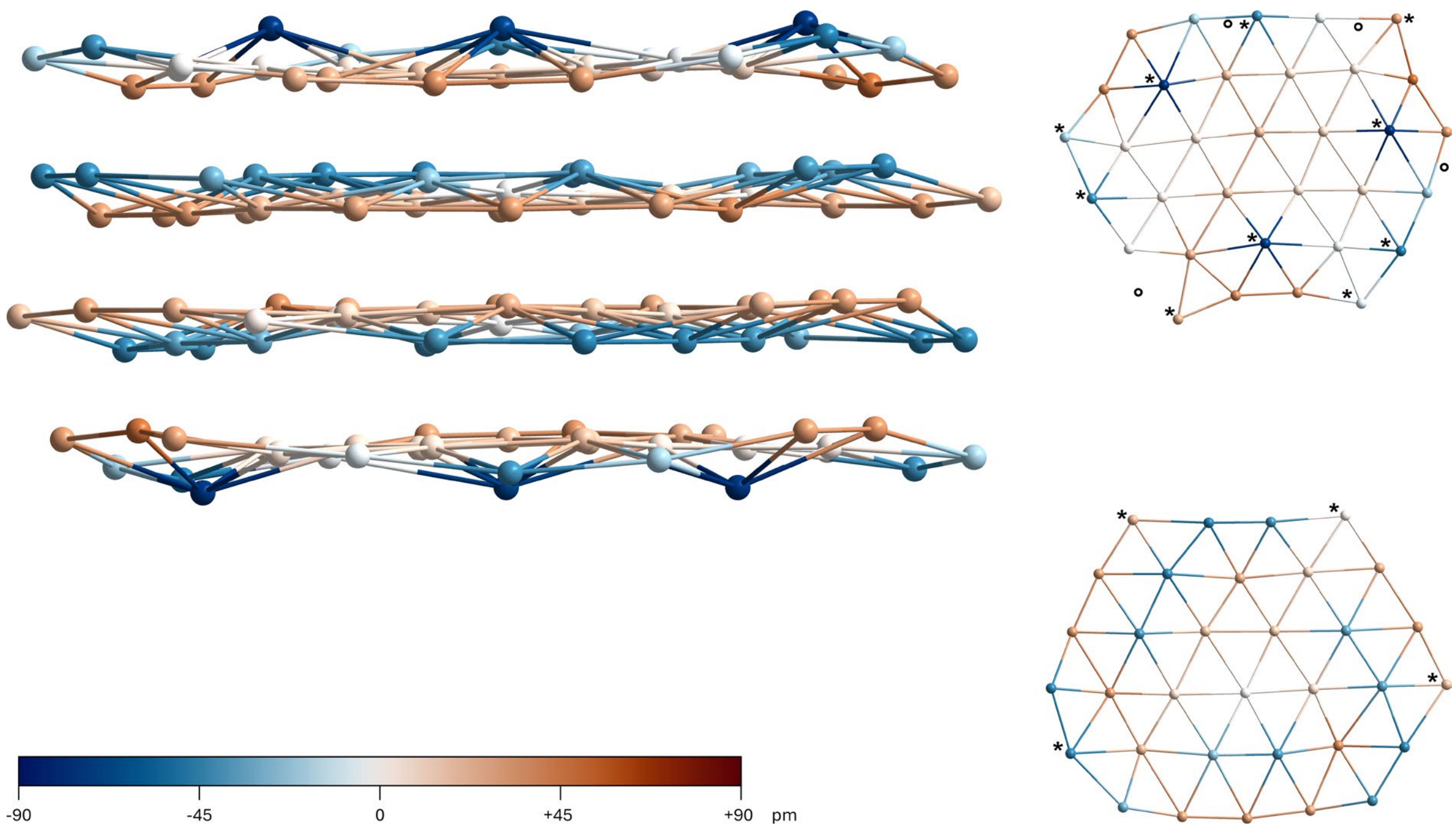

Figure S9. Atomic out-of-plane displacements found within the metal kernel of $\mathbf{Ag_{132}}$. Left: side view of the cluster. Right: Top view of the outer (top) and inner (bottom) layers. *Silver atoms bound to P. °Bond between silver atoms bound to $\mu^{2/3}$-Cl.

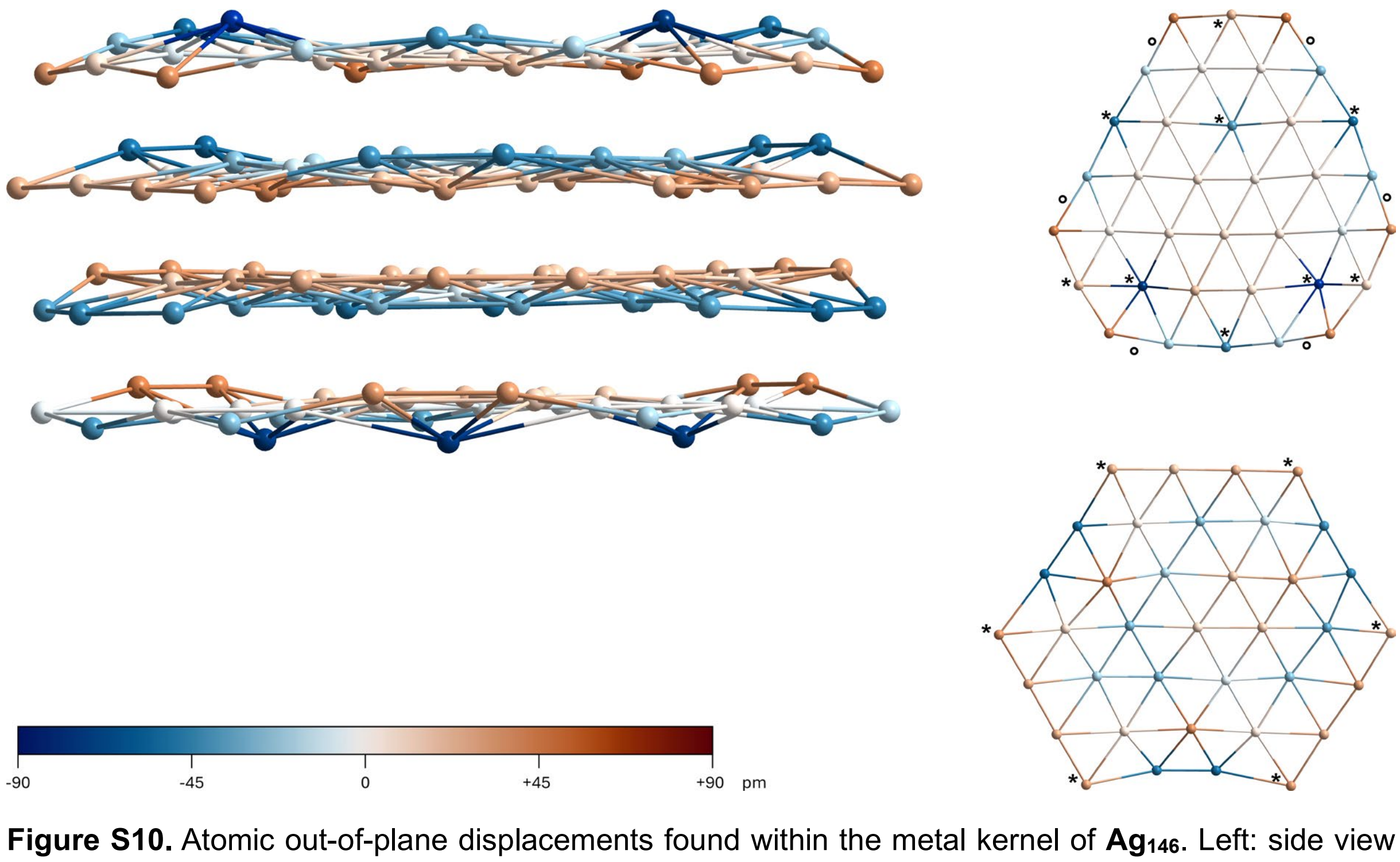

Figure S10. Atomic out-of-plane displacements found within the metal kernel of $\mathbf{Ag_{146}}$. Left: side view of the cluster. Right: Top view of 1st (top) and 2nd (bottom) layer. *Silver atoms bound to P. °Bond between silver atoms bound to μ^{2}-Cl.

2.5. Space-Filling Models of **Ag_{128}**, **Ag_{132}** and **Ag_{146}**

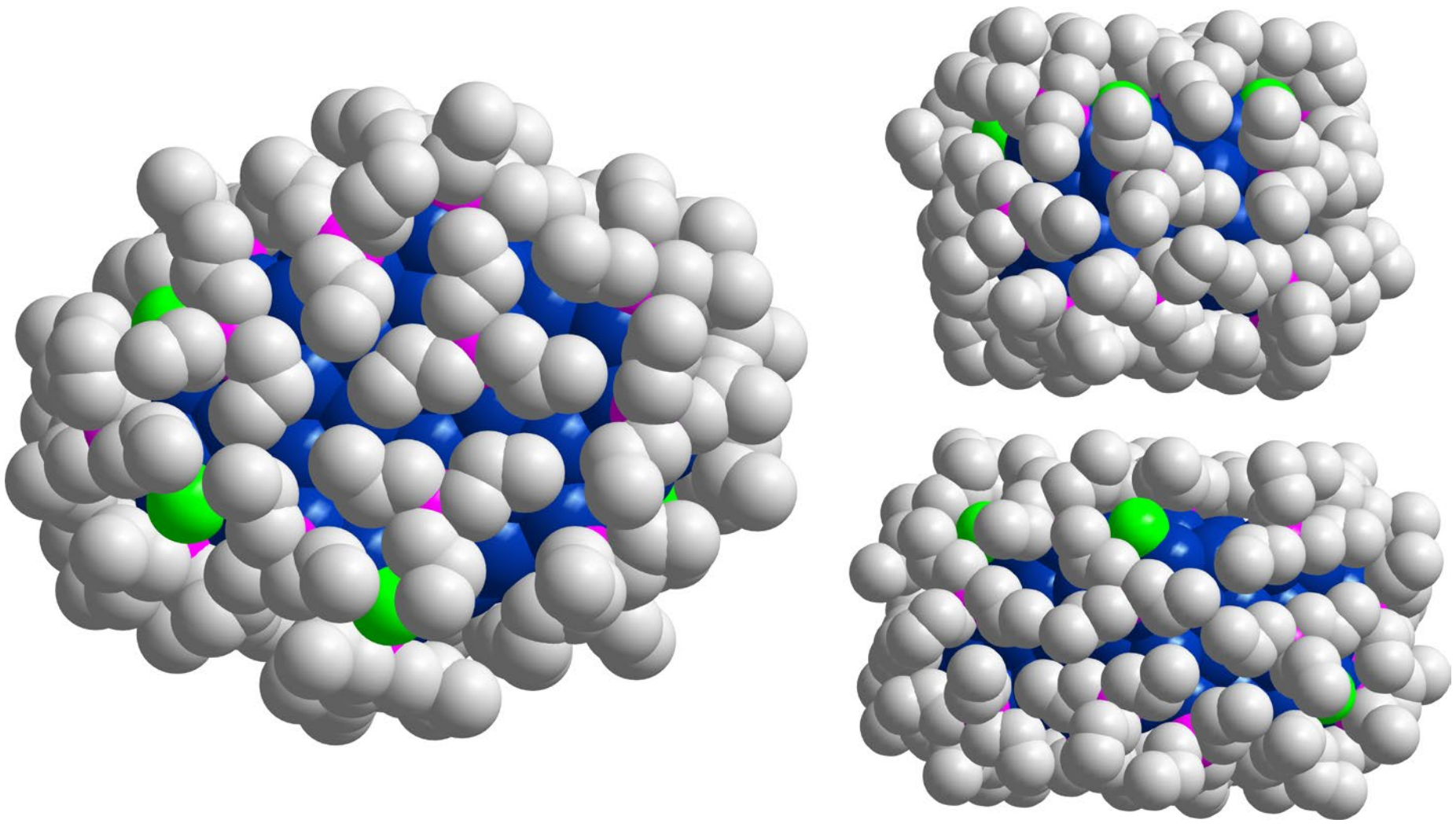

Figure S11. Space-filling model of **Ag_{128}**'s molecular structure. Left: top view. Right: side views. Ag (blue), P (pink), Cl (green), C (grey).

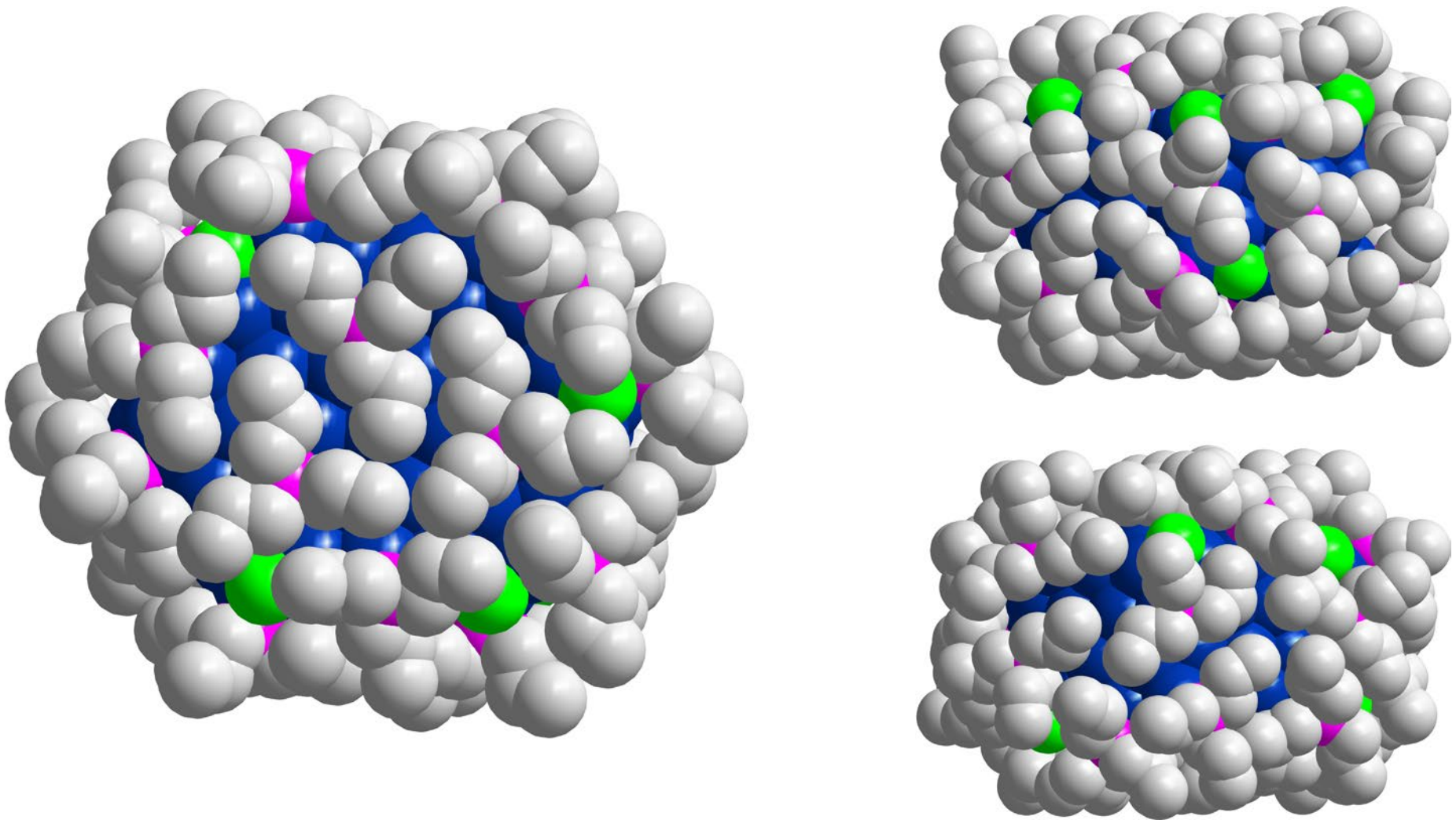

Figure S12. Space-filling model of **Ag_{132}**'s molecular structure. Left: top view. Right: side views. Ag (blue), P (pink), Cl (green), C (grey).

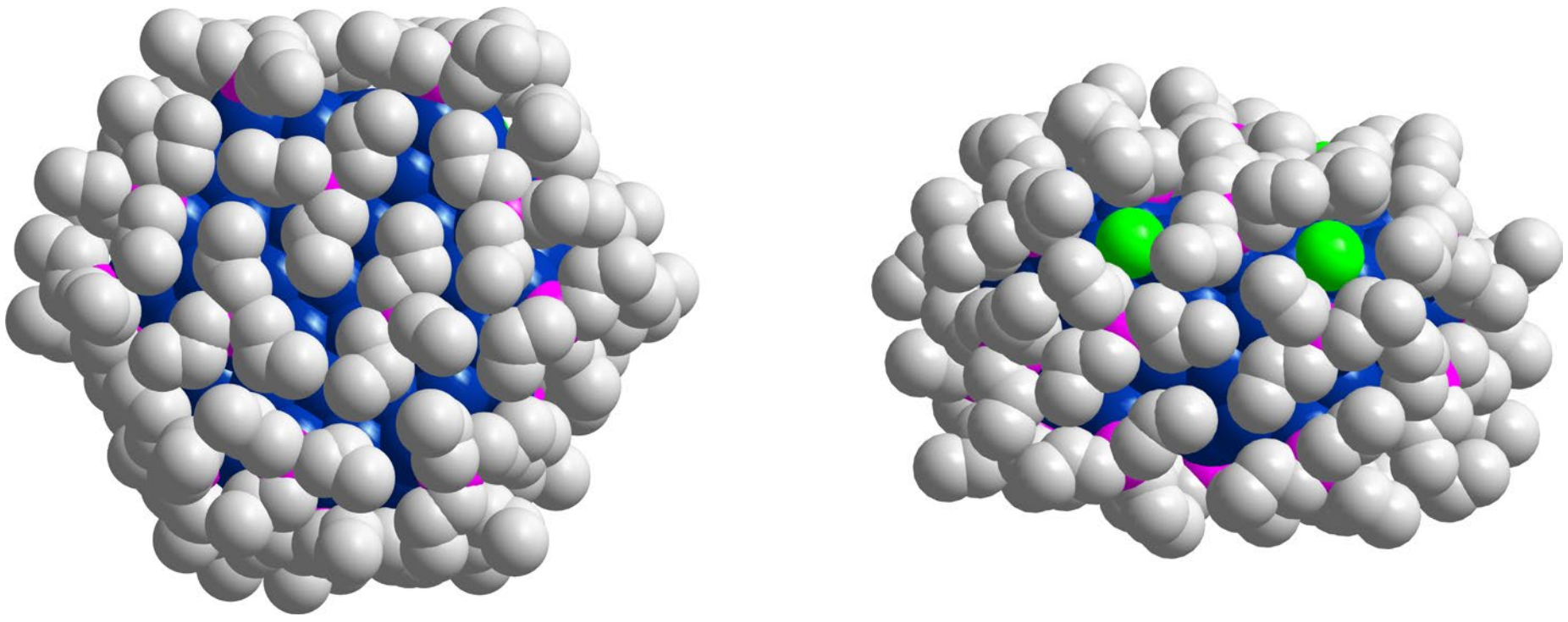

Figure S13. Space-filling model of **Ag_{146}**'s molecular structure. Left: top view. Right: side view. Ag (blue), P (pink), Cl (green), C (grey).

2.6. Comparison Between the Title Compounds and hcp Lattice

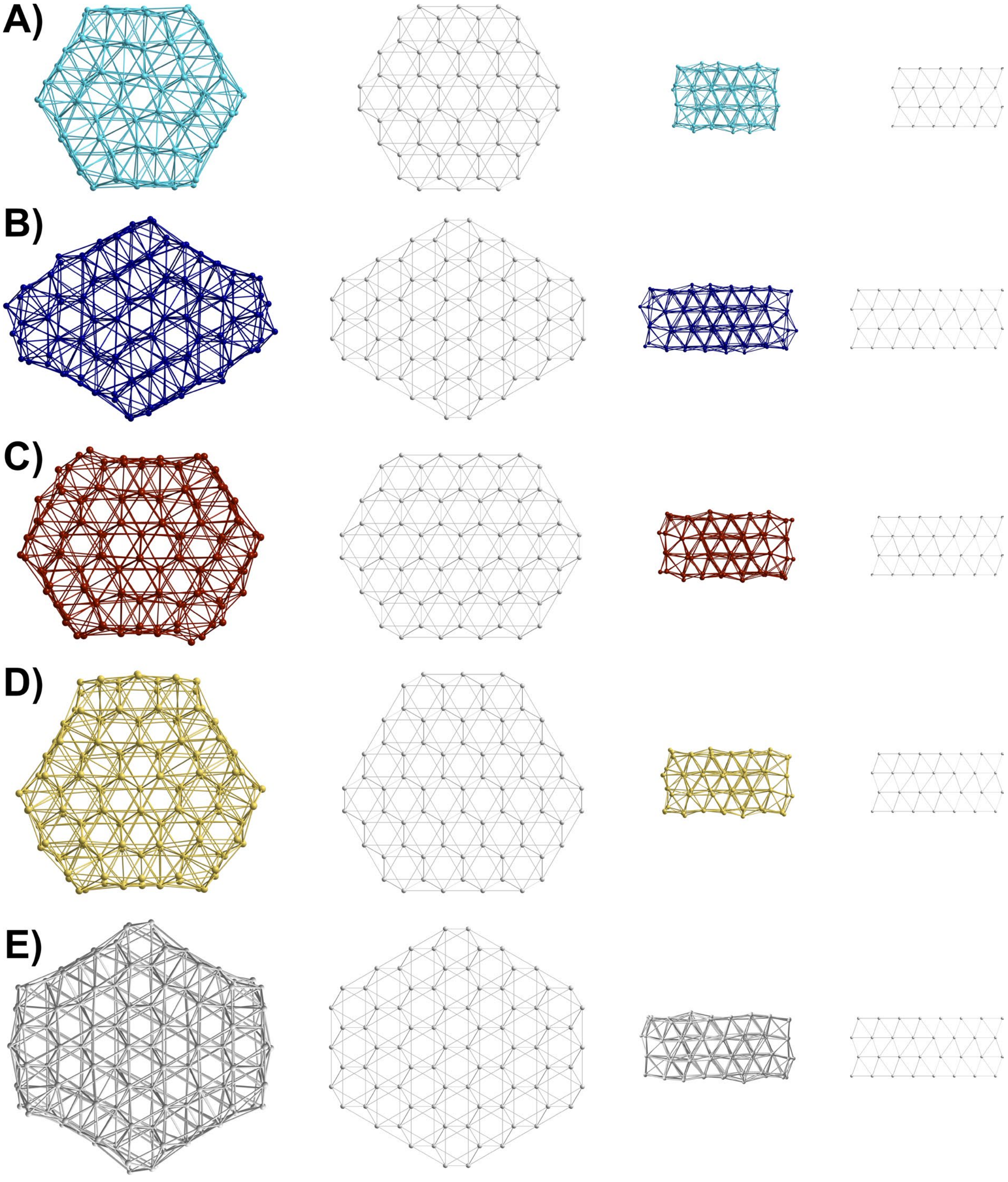

Figure S14. Comparison between the metal kernels of $\mathbf{Ag_{108}}$ (A, cyan), $\mathbf{Ag_{128}}$ (B, blue), $\mathbf{Ag_{132}}$ (C, red), $\mathbf{Ag_{146}}$ (D, yellow), and $\mathbf{Ag_{160}}$ (E, gray) with hcp fragments containing the same number of atoms (fine lines). Even though concave/convex cluster facets and distortion in general lead to deviation from an ideal hcp arrangement, the hexagonal prismatic shape and the atom positions within the title compounds strongly relate to their respective ideal hcp models. After $\mathbf{Ag_{108}}$, the next largest regular hexagonal prism with 4 layers would consist of 192 atoms.

2.7. Further Comparison Between the Title Compounds

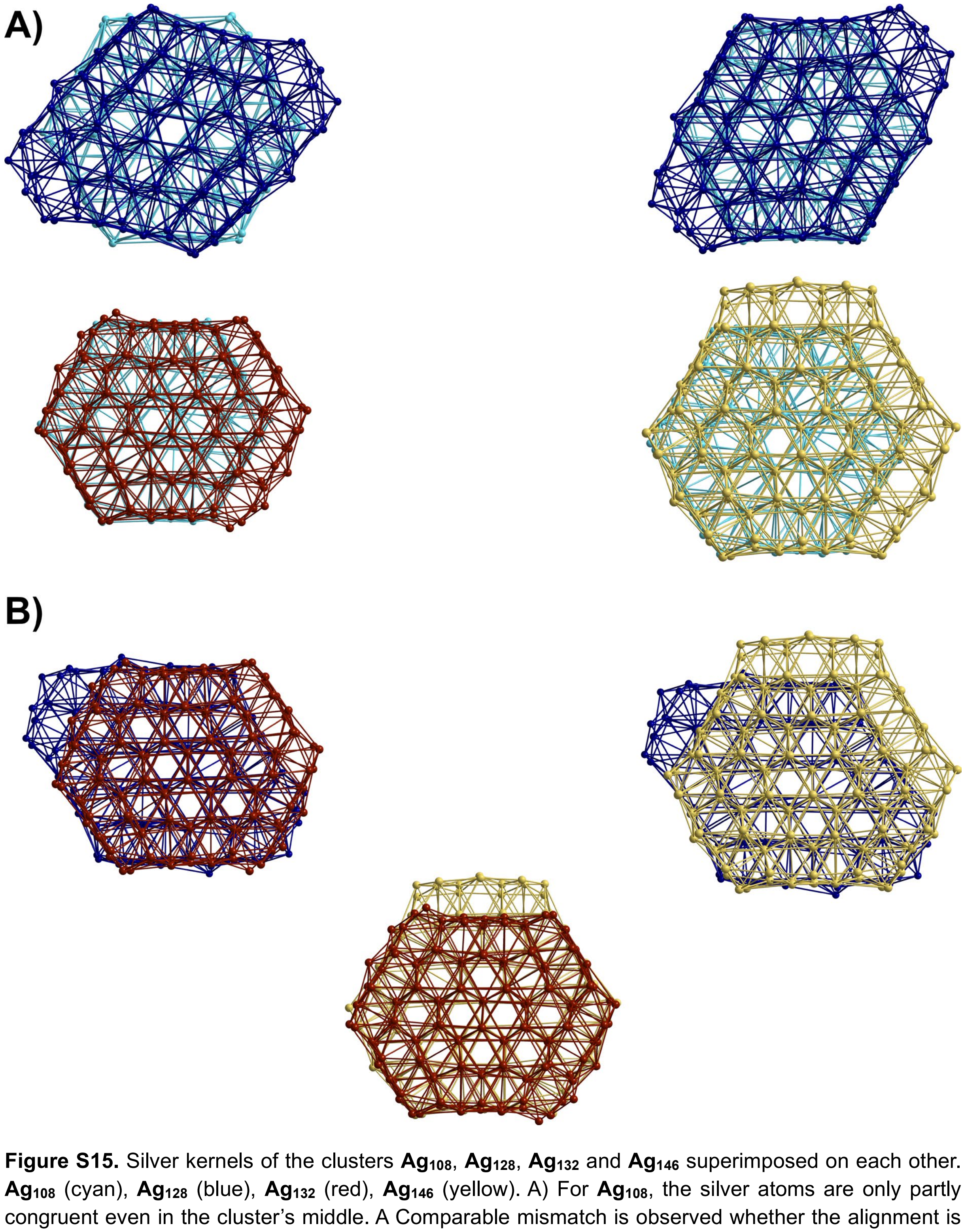

Figure S15. Silver kernels of the clusters $\mathbf{Ag_{108}}$, $\mathbf{Ag_{128}}$, $\mathbf{Ag_{132}}$ and $\mathbf{Ag_{146}}$ superimposed on each other. $\mathbf{Ag_{108}}$ (cyan), $\mathbf{Ag_{128}}$ (blue), $\mathbf{Ag_{132}}$ (red), $\mathbf{Ag_{146}}$ (yellow). A) For $\mathbf{Ag_{108}}$, the silver atoms are only partly congruent even in the cluster's middle. A Comparable mismatch is observed whether the alignment is anchored at the central hexagon or at a cluster edge. B) For the other three clusters, good overall superimposition is found, whereby even Ag—Ag bonds are mainly congruent.

3. SEM and EDX Measurements

Scanning electron microscopy (SEM) and energy-dispersive X-ray spectroscopy (EDX) data were collected on a HITACHI SU 8030 equipped with a Bruker QUANTAX 6G EDX detector at an acceleration voltage of 5.0 to 30.0 kV. For SEM measurements, the acquisition mode is identified in each image: TE (transmission mode), SE (secondary electron mode).

Samples were prepared as follows: single crystals of the respective compound were dissolved in toluene precooled to –40°C. The resulting solution was then filtered, and one drop of the dilute solution was put onto the copper grid. Since preceding experiments did not show differences between pseudo-inert and ambient conditions, drop casting and subsequent storage were performed under ambient conditions.

3.1. Ag_{128}

As can be seen from Figure S16, we were able to identify silver nanoparticles of different shapes and sizes. Nonetheless, for $\mathbf{Ag_{128}}$ all geometrically defined particles had triangles/hexagons as their base shape. The relative flatness of the nanoprisms was also confirmed by the combination of transmitted and secondary electron data (Figure S17).

During measurement, we observed several nanoparticles that appeared to “melt” under the electron beam (Figure S18). Only a fraction of the observed particles showed this behavior, with no correlation between melting and observable factors such as nanoparticle thickness, size, or shape.

We were also interested in whether nanoprism formation is based on thermolysis or photolysis. Therefore, we took two samples of a toluene solution of $\mathbf{Ag_{128}}$ and prepared them as follows: after drop casting, one sample was left at room temperature in the dark room for 3 h. The other one was cooled with dry ice and irradiated by UV light with a wavelength of 254 nm and 365 nm for 90 min respectively before warming up. Comparison shows that for the latter one no comparable nanoprisms were found (Figure S20). Nanoparticles formed during thermolysis did not show significant differences after irradiation; especially nanoprism size, form and concentration did not appear to change (Figure S21).

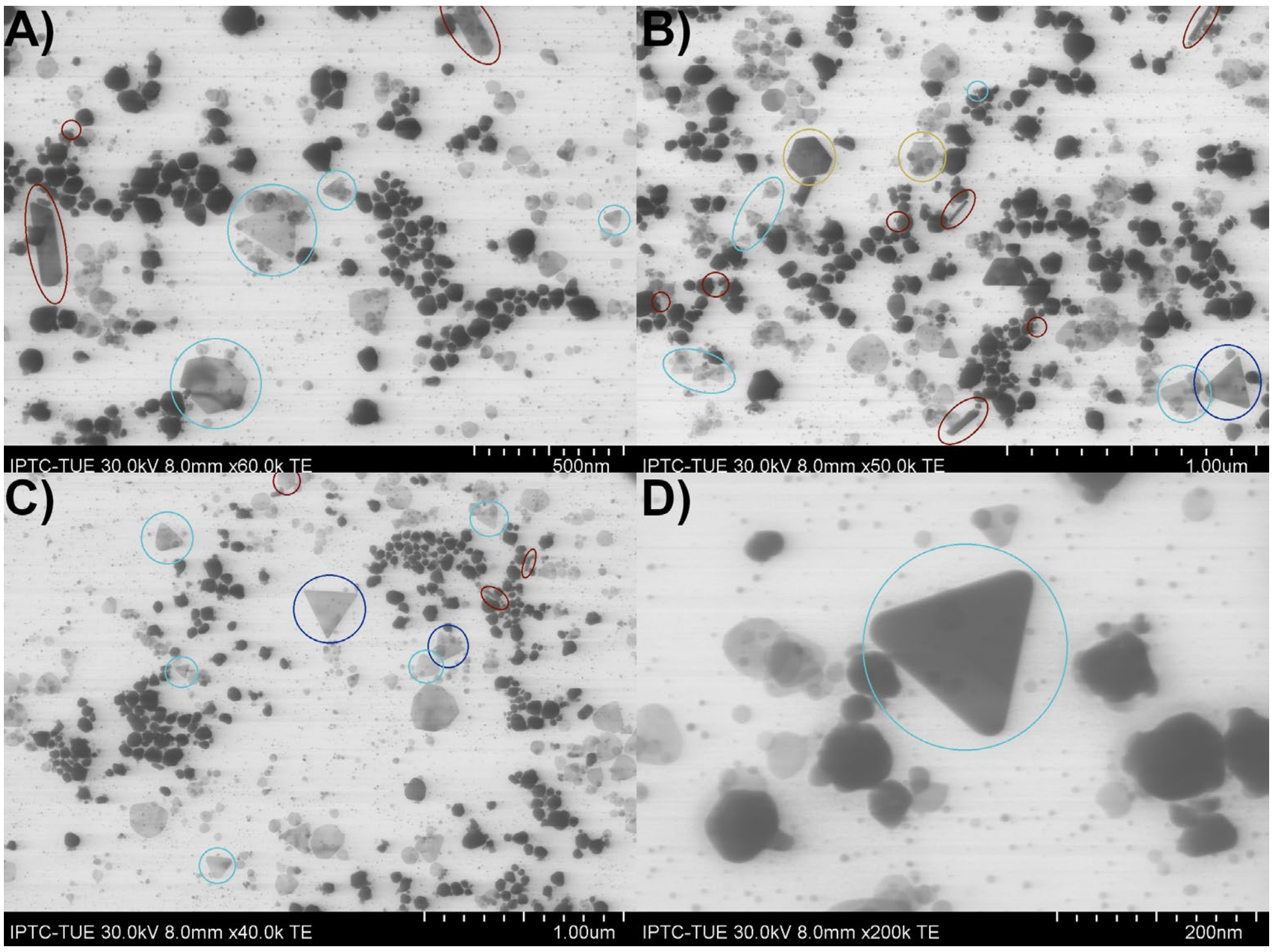

Figure S16. SEM image (STEM mode) of the decomposition product of $\mathbf{Ag_{128}}$. Between undefined particles, nanoprisms with a triangular (sharp vertices: blue, rounded vertices: cyan) or hexagonal (yellow) base are present. Some nanoparticles stand upright (red), giving insights into their profile.

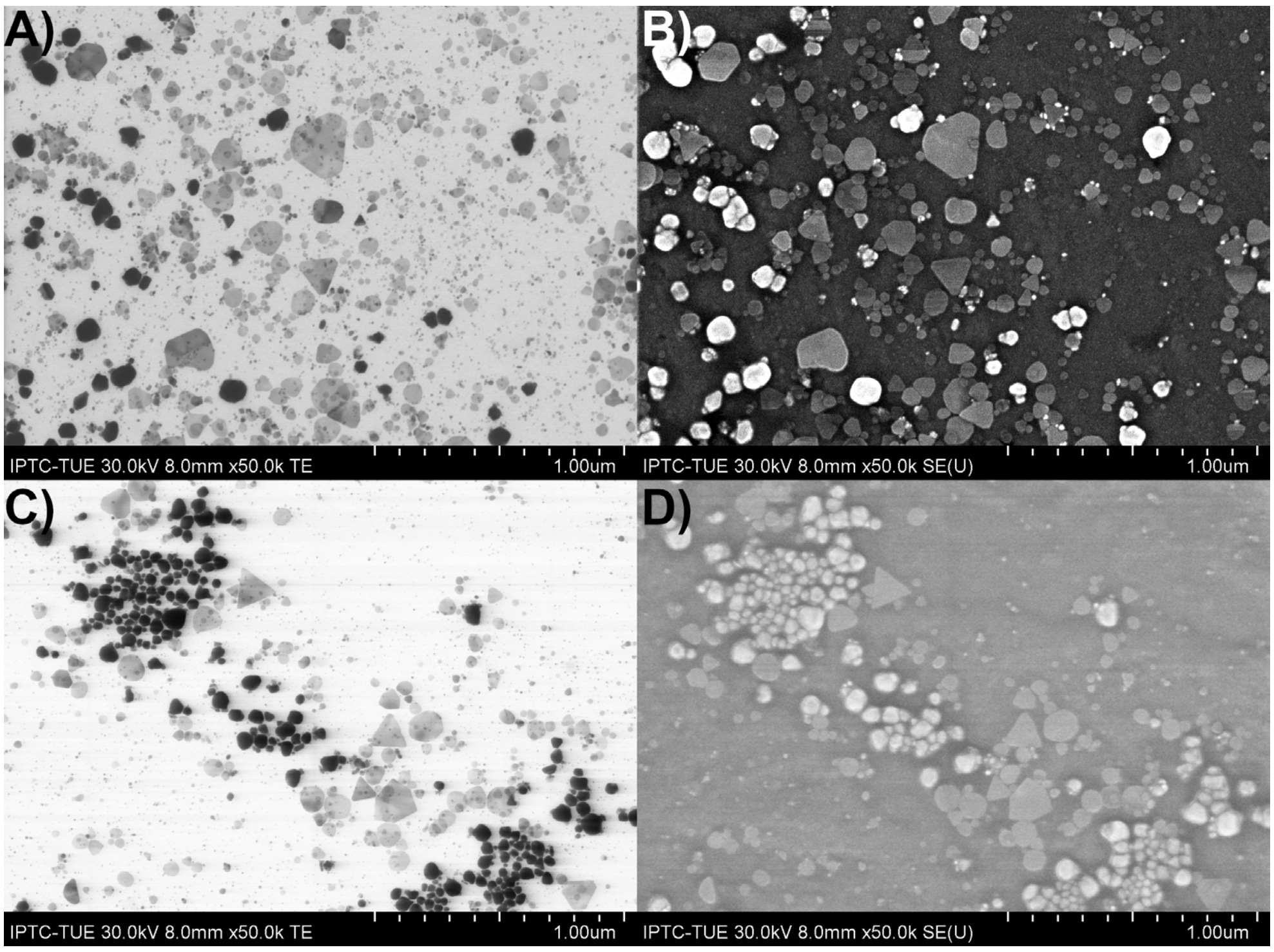

Figure S17. Two randomly selected sample areas. Images have been acquired in transmission (A,C) and secondary electron (B,D) mode. Nanoprisms with defined shapes appear thinner than particles without a defined shape.

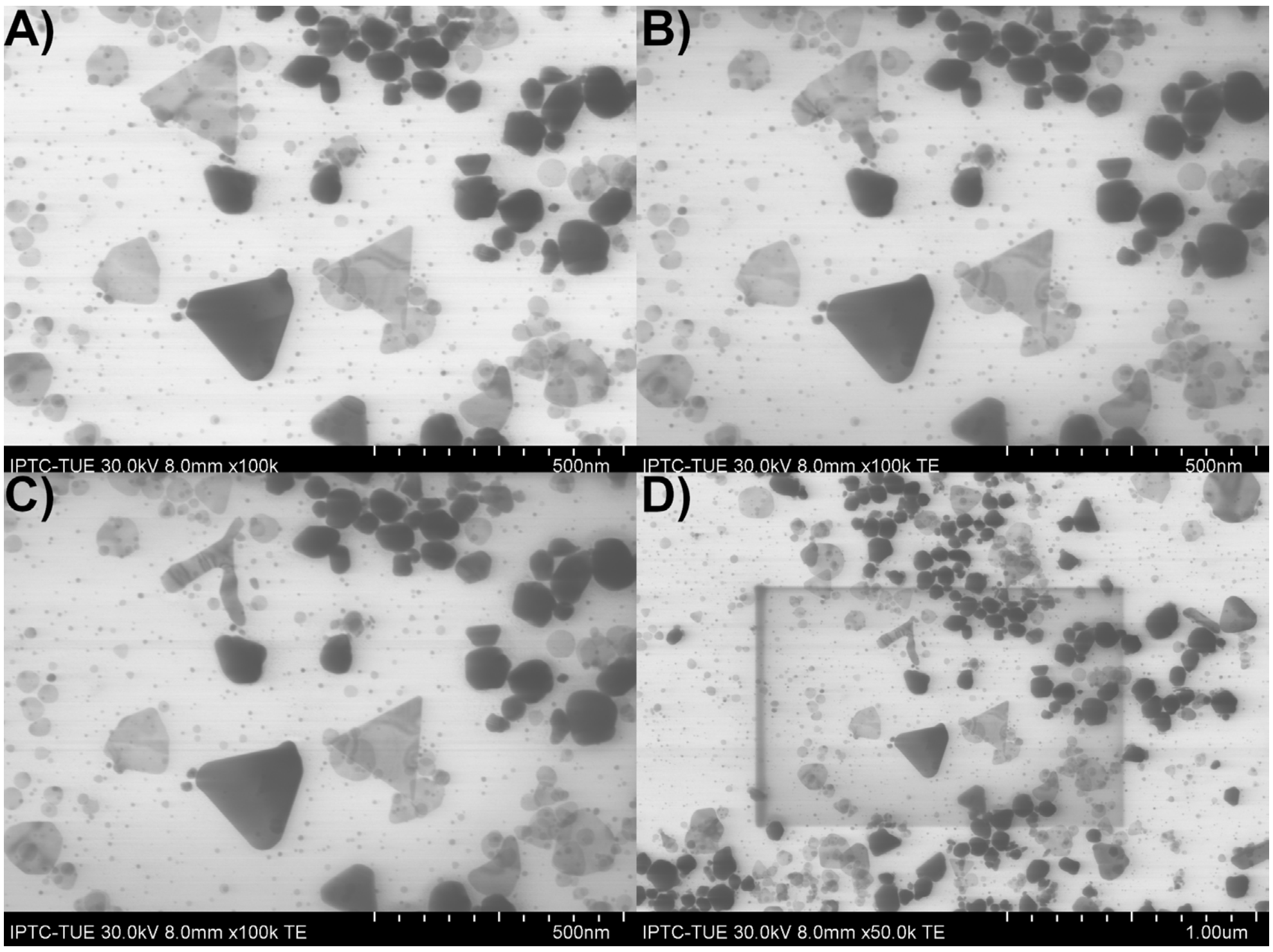

Figure S18. A-C) Images taken 1 min apart each. One nanoprism appears to melt during continuous measurement. Meanwhile, several neighboring nanoparticles do not show this behavior. D) Contamination caused by the electron beam after 3 min of continuous measurement.

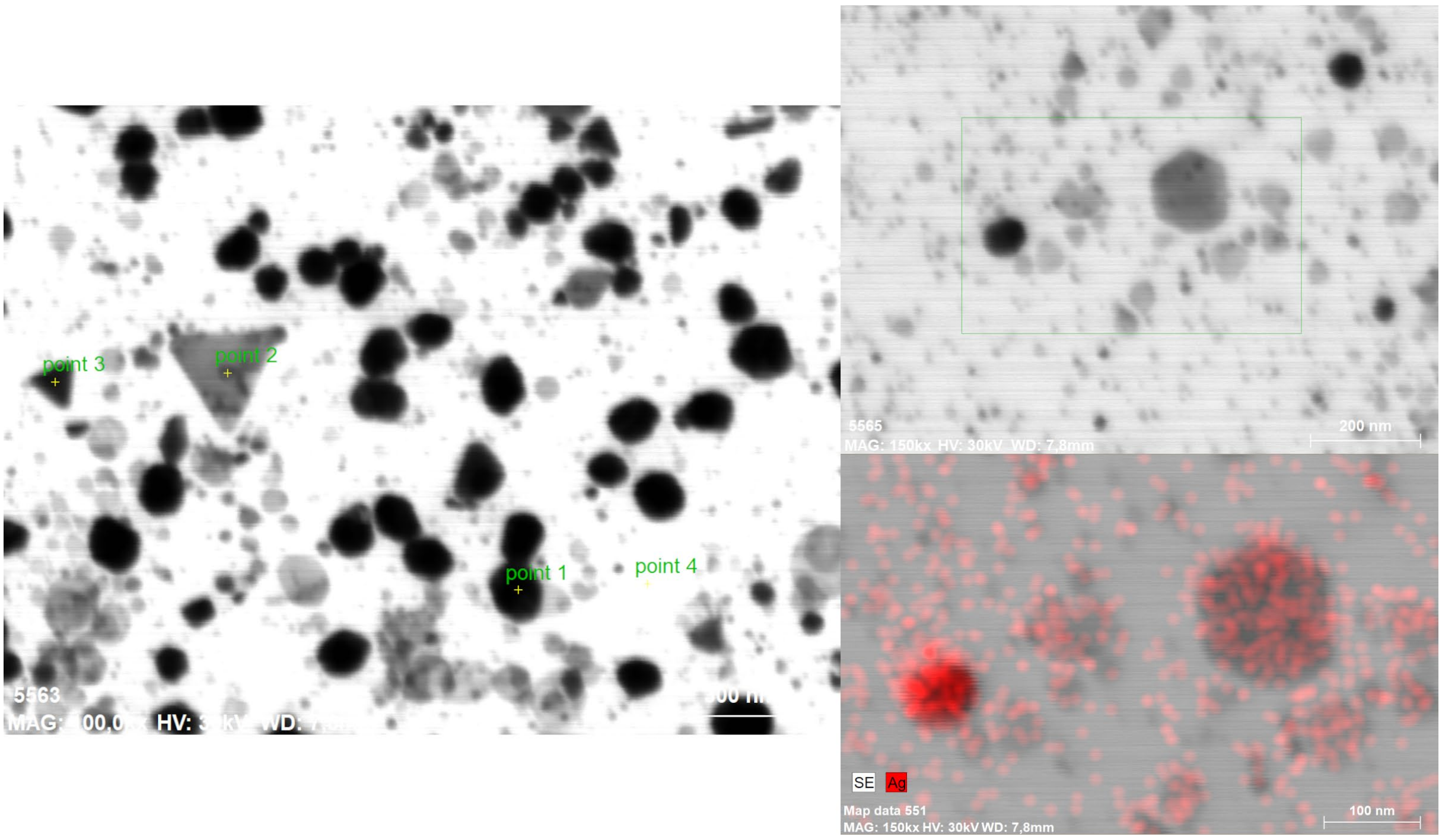

Figure S19. Left: Measurement points for EDX analysis (data are found in Table S11). Right: Measurement area for a map scan (top) and the corresponding Ag signals found (bottom). Both measurements have been performed for several sample areas and are in agreement with each other.

Table S11. Normalized wt% for Ag, P and Cl for measurement points 1-4.

	Ag	P	Cl
Point 1	99	1	0
Point 2	99	1	0
Point 3	99	1	0
Point 4[1]	96	3	2

[1]For point 4, the concentration before normalization (without C) was significantly lower than for points 1-3.

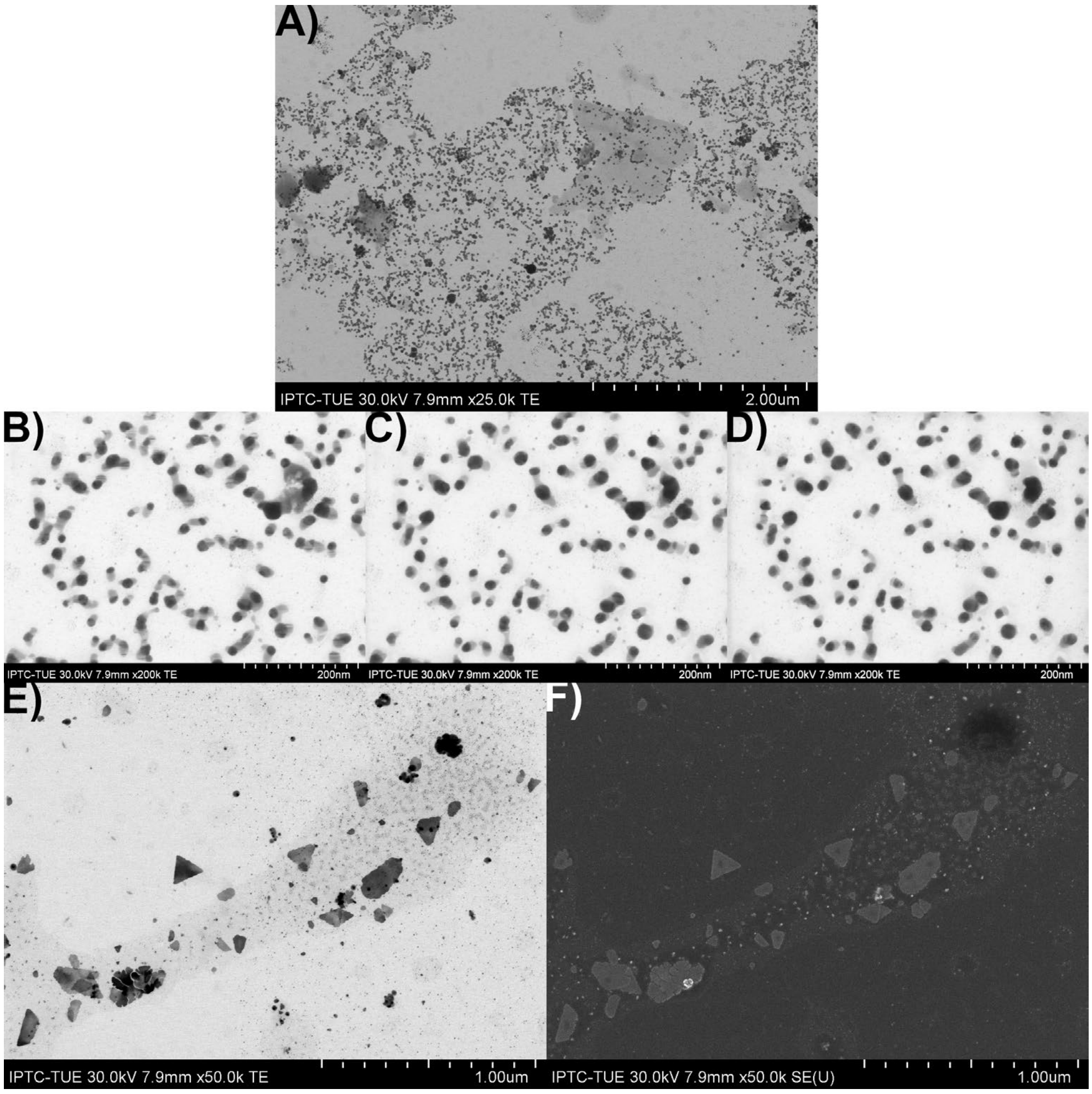

Figure S20. SEM images of a **Ag_{128}** sample irradiated by UV light (254 nm and 365 nm, 90 min each). A) Overview. Particles shown are not nanoprisms but have no defined shape. B-D) Time-lapse of changes under electron beam irradiation. The whole sample is highly dynamic. There is a 2 min gap between images. E-F) Another section examined in transmission and secondary electron mode. Several flat (partly truncated) triangular nanoparticles can be seen. Compared to nanoprisms found for thermolysis (*vide supra*), edges are coarse, intraparticle thickness is not uniform and occurrence is rarer.

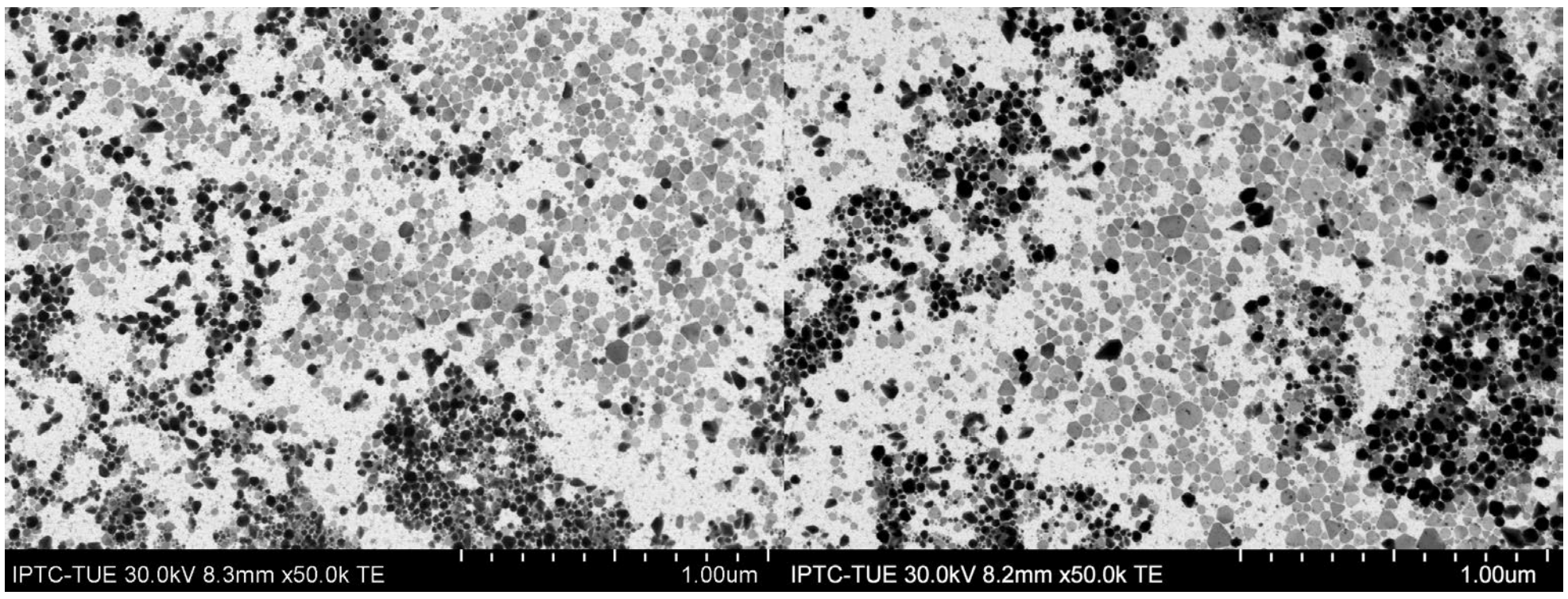

Figure S21. Comparison between nanoparticles formed by thermolysis of **Ag_{128}** before (left) and after (right) subsequent irradiation with UV light (254 nm and 365 nm) for 2 h. No significant changes in nanoprism shape or concentration are observed. Multiple areas have been probed to exclude regional deviations. Note that while the sample is the same in both cases, images before and after irradiation show different areas of the sample.

3.2. Other Compounds

While it seems plausible that the title compounds act as seeds for the emerging nanoprisms, formation of the latter ones does not constitute definitive proof. Syntheses of anisotropic noble metal nanoparticles that do not rely on seeded growth are known.[9] Thus, we wanted to test whether our clusters' properties or rather the experimental conditions themselves cause the formation of nanoprisms. We prepared additional samples for the clusters **Ag_{108}**, **Ag_{132}**, **Ag_{64}** and the precursor (Pr_3P)AgCl. For the larger clusters, we indeed found nanoprisms, while we did not for the latter two compounds (Figure S24).

Interestingly, for **Ag_{108}** we found several icosahedral nanoparticles as well (Figure S22). For **Ag_{132}**, many nanosheets appeared to be round rather than faceted. We noticed that upon electron beam irradiation, the nanoprisms melted even faster than for **Ag_{128}** (*vide supra*). Still, we were able to document this phenomenon (Figure S23). Since here, the average nanoprism size is smaller than for **Ag_{128}** samples, nanoparticle size might be an important factor. It might be possible that the round spots found within the **Ag_{64}** sample therefore used to be defined nanoprisms before their fast decomposition due to their even smaller size.

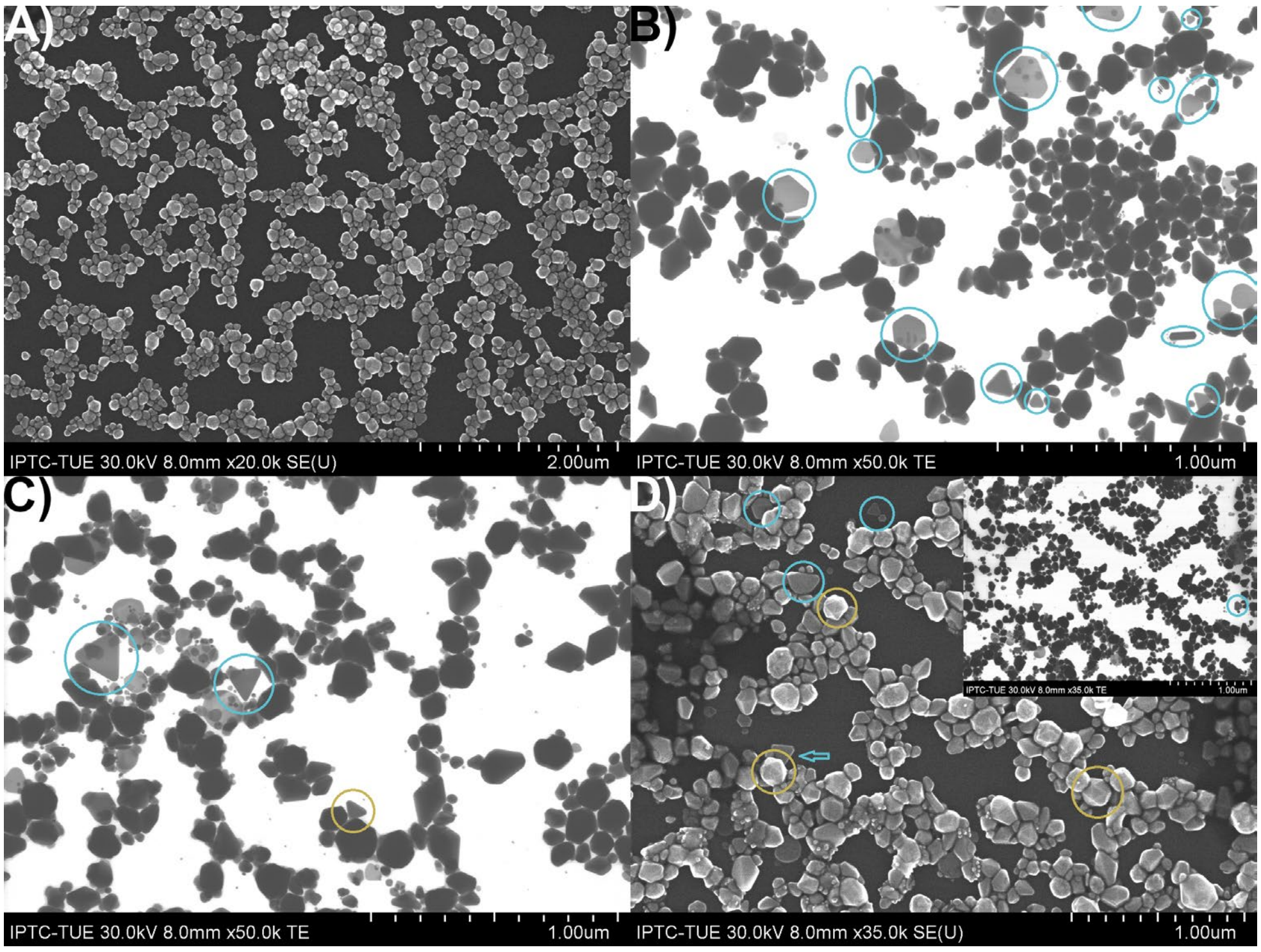

Figure S22. SEM images of a **Ag_{108}** thermolysis sample. Trigonal and hexagonal nanoprisms (cyan) can be found. Yet, the ratio nanoprism:other nanoparticles is significantly lower compared to **Ag_{128}** thermolysis. A) Overview. Agglomeration of nanoparticles into net-like structures can be observed. B-C) In transmission mode, flat nanoprisms can be discerned from bulkier nanoparticles. The latter can feature sharp facets as well. D) Within the sample, icosahedral nanoparticles (yellow) are present as well. Inset: in transmission mode, facets and twin boundaries of bulky nanoparticles cannot be seen.

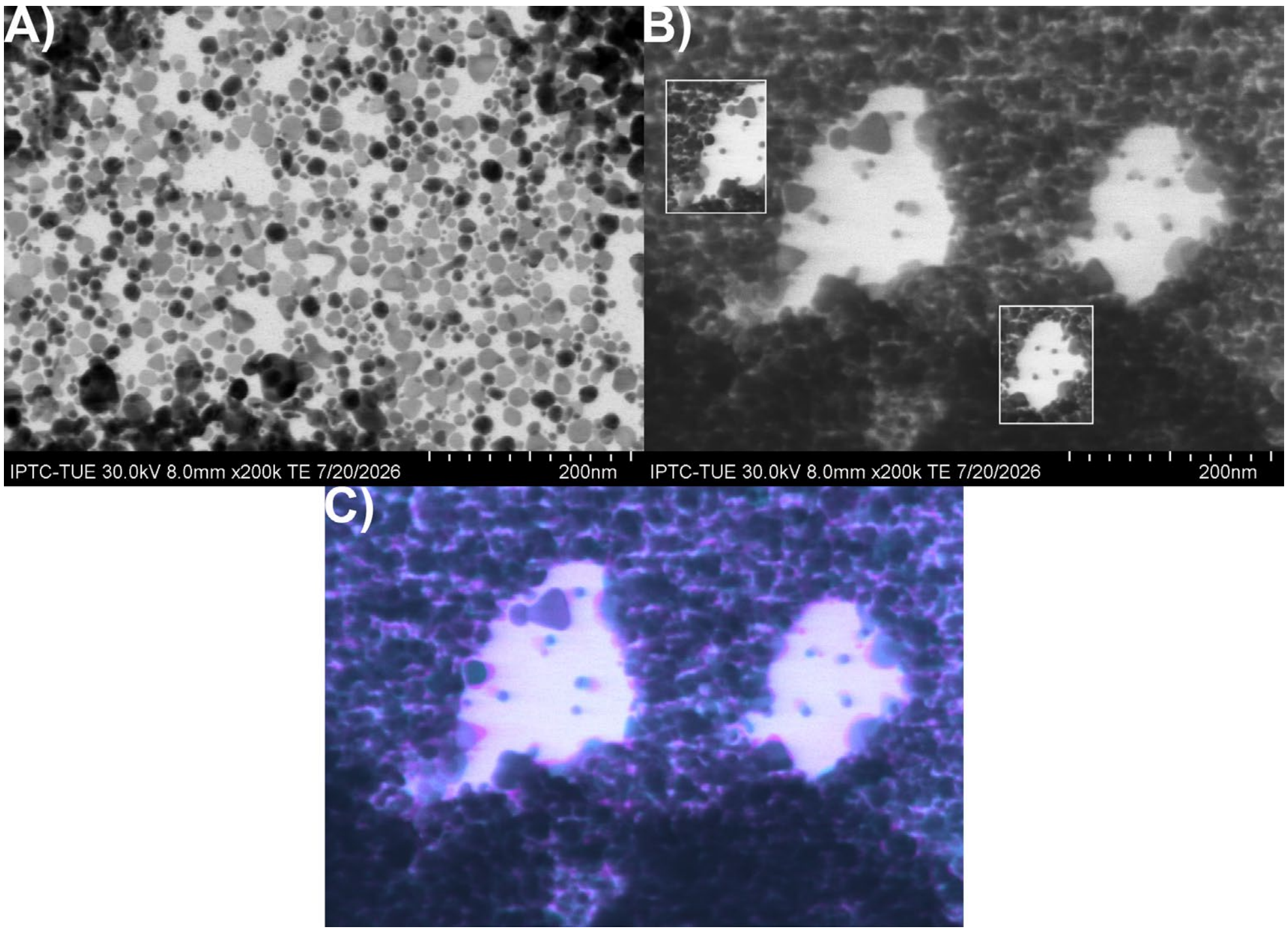

Figure S23. SEM images of a **Ag_{132}** thermolysis sample. A) While nanoprisms with a defined, sharp outline are present, they are accompanied by many rather round nanodiscs. B) Occurrence of round nanoparticles can be explained by beam-induced sample deterioration. Initially, defined nanoprisms are found. Within seconds of irradiation they melt, as shown in the insets. C) Color-coded differential image before (magenta) and after (cyan) nanoparticle deterioration.

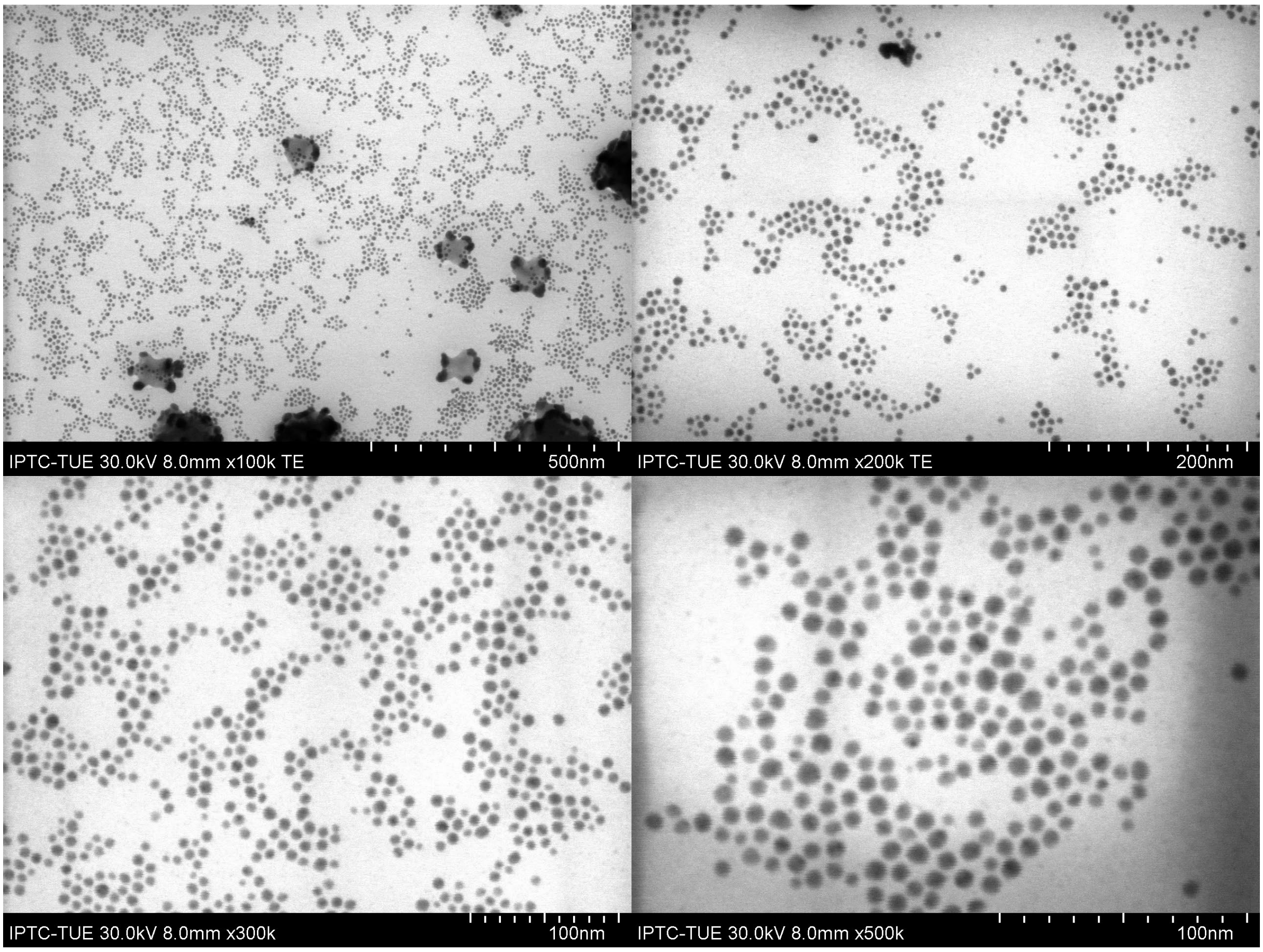

Figure S24. SEM images of a $\mathbf{Ag_{64}}$ thermolysis sample with increasing magnification. Many small, well separated particles without sharp edges/vertices are present. One possibility why no faceted nanoprisms can be found might be that sample decomposition (i.e. melting) is too fast due to their small size.

4. Quantum Chemical Calculations

The calculations were done using density functional theory as implemented in the code package GPAW using real-space grids with grid spacing 0.25 Å[10] and Perdew-Burke-Ernzerhof exchange-correlation functional.[11] Starting structures for ligand-protected $\mathbf{Ag_{108}}$, $\mathbf{Ag_{128}}$ and $\mathbf{Ag_{132}}$ clusters were taken directly from experimental crystal structures and for $\mathbf{Ag_{146}}$ and $\mathbf{Ag_{160}}$ from the partially resolved experimental structures that were completed to generate full model clusters, e.g. by adding the missing atoms of the ligand layer. Structures were first optimized until the net forces on all atoms were below 0.05 eV/Å and then for the optimized structures the density of electron states were analyzed using grid spacing. We also calculated and analyzed the density of states for pure metal clusters in the exact same structures as the metal cores of the protected clusters. For pure metal clusters without ligands the count of free valence electrons was chosen to be the same as in the fully protected parent cluster by matching the total charge to the number of electron withdrawing ligands in the original cluster.

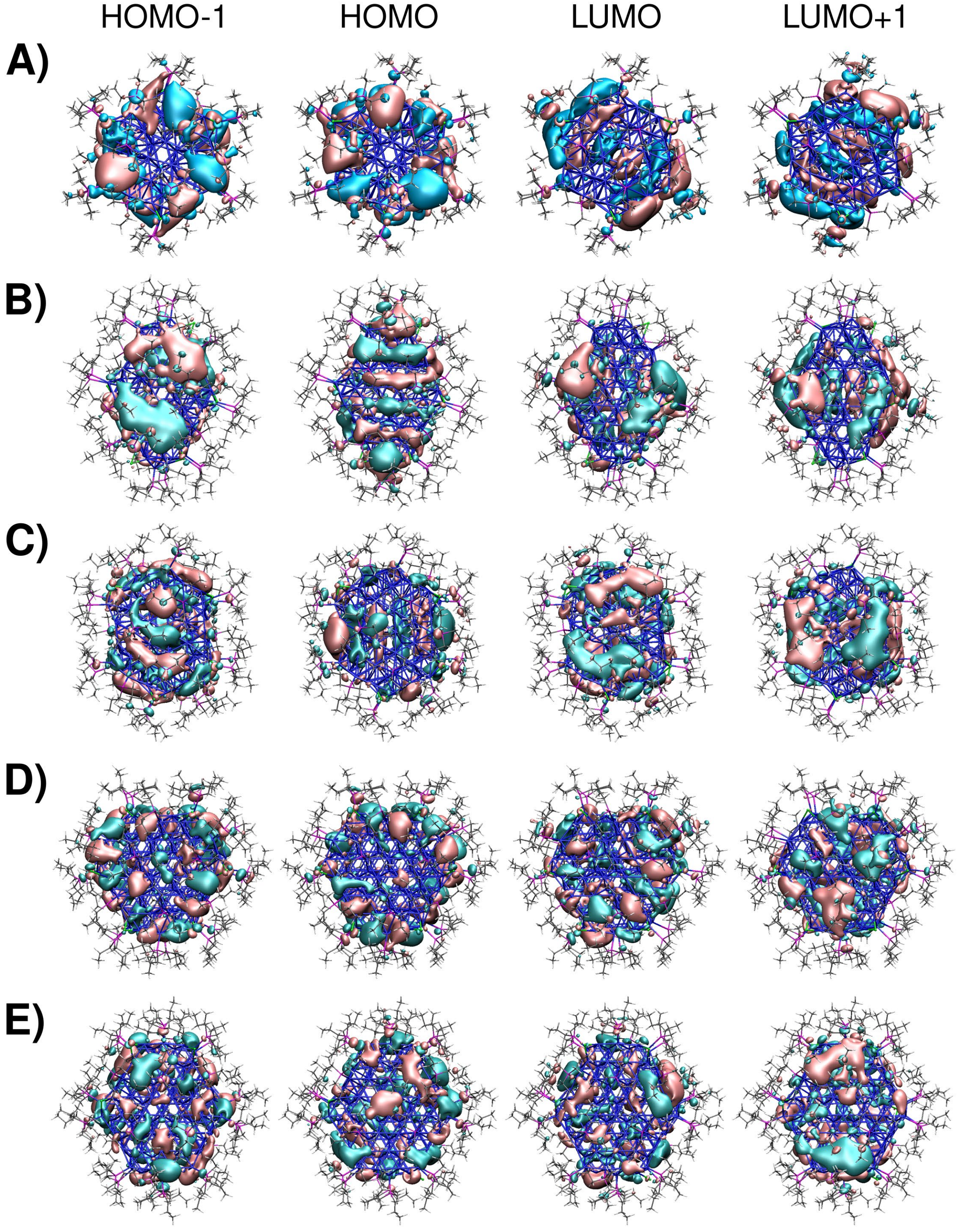

Figure S25. Frontier cluster orbitals of A) $\mathbf{Ag_{108}}$, B) $\mathbf{Ag_{128}}$, C) $\mathbf{Ag_{132}}$, D) $\mathbf{Ag_{146}}$ and E) $\mathbf{Ag_{160}}$.

5. On the Colors Used

We used the uniform color maps Batlow, Roma and Vik[12] for most of our figures. These color maps are designed to remain interpretable for readers with color-vision deficiency and in greyscale. Furthermore, their use reduces visual distortions, which is especially important for gradient-type figures.[13]

6. References

[1] (a) M. Diecke, C. Schrenk and A. Schnepf, *Angew. Chem. Int. Ed.*, 2020, **59**, 14418–14422. (b) M. R. Churchill, J. Donahue and F.-J. Rotella, *Inorg. Chem.*, 1976, **15**, 2752–2758.

[2] (a) G. M. Sheldrick, *Acta Crystallogr.*, Sect. *A*, 2008, **64**, 112-122. (b) G. M. Sheldrick, *Acta Crystallogr.*, Sect. *C*, 2015, **71**, 3–8.

[3] O. V. Dolomanov, L. J. Bourhis, R. J. Gildea, J. A. K. Howard and H. Puschmann, *J. Appl. Crystallogr.,* 2009, **42**, 339–341.

[4] (a) D. Kratzert, J. J. Holstein and I. Krossing, *J. Appl. Crystallogr.*, 2015, **48**, 933-938. (b) D. Kratzert and I. Krossing, *J. Appl. Crystallogr.*, 2018, **51**, 928–934.

[5] A. L. Spek, *Acta Crystallogr., Sect. D*, 2009, **65**, 148–155.

[6] M. Pinsky and D. Avnir, *Inorg. Chem.*, 1998, **37**, 5575–5582.

[7] M. Llunell, D. Casanova, J. Cirera, P. Alemany and S. Alvarez, SHAPE. Program for the Stereochemical Analysis of Molecular Fragments by Means of Continuous Shape Measures and Associated Tools, Version 2.1, Universitat de Barcelona, Barcelona, 2013.

[8] Diamond - Crystal and Molecular Structure Visualization, Crystal Impact - Dr. H. Putz & Dr. K. Brandenburg GbR, Kreuzherrenstr. 102, 53227 Bonn, Germany, https://www.crystalimpact.de/diamond.

[9] (a) Y. Sun and Y. Xia, *Science*, 2002, **298**, 2176–2179. (b) C. Lofton and W. Sigmund, *Adv. Funct. Mater.*, 2005, **15**, 1197–1208.

[10] J. J. Mortensen, A. H. Larsen, M. Kuisma, A. V. Ivanov, A. Taghizadeh, A. Peterson, A. Haldar, A. O. Dohn, C. Schäfer, E. Ö. Jónsson, E. D. Hermes, F. A. Nilsson, G. Kastlunger, G. Levi, H. Jónsson, H. Häkkinen, J. Fojt, J. Kangsabanik, J. Sødequist, J. Lehtomäki, J. Heske, J. Enkovaara, K. T. Winther, M. Dulak, M. M. Melander, M. Ovesen, M. Louhivuori, M. Walter, M. Gjerding, O. Lopez-Acevedo, P. Erhart, R. Warmbier, R. Würdemann, S. Kaappa, S. Latini, T. M. Boland, T. Bligaard, T. Skovhus, T. Susi, T. Maxson, T. Rossi, X. Chen, Y. L. A. Schmerwitz, J. Schiøtz, T. Olsen, K. W. Jacobsen and K. S. Thygesen, *J. Chem. Phys.*, 2024, **160**, 092503.

[11] J. P. Perdew, K. Burke and M. Ernzerhof, *Phys. Rev. Lett.*, 1996, **77**, 3865–3868.

[12] F. Crameri, *Zenodo* 2018, https://doi.org/10.5281/zenodo.1243862.

[13] F. Crameri, G. E. Shephard and P. J. Heron, *Nat. Commun.*, 2020, **11**, 5444.
